# Targeting myeloid chemotaxis to reverse prostate cancer therapy resistance

**DOI:** 10.1038/s41586-023-06696-z

**Published:** 2023-10-16

**Authors:** Christina Guo, Adam Sharp, Bora Gurel, Mateus Crespo, Ines Figueiredo, Suneil Jain, Ursula Vogl, Jan Rekowski, Mahtab Rouhifard, Lewis Gallagher, Wei Yuan, Suzanne Carreira, Khobe Chandran, Alec Paschalis, Ilaria Colombo, Anastasios Stathis, Claudia Bertan, George Seed, Jane Goodall, Florence Raynaud, Ruth Ruddle, Karen E. Swales, Jason Malia, Denisa Bogdan, Crescens Tiu, Reece Caldwell, Caterina Aversa, Ana Ferreira, Antje Neeb, Nina Tunariu, Daniel Westaby, Juliet Carmichael, Maria Dolores Fenor de la Maza, Christina Yap, Ruth Matthews, Hannah Badham, Toby Prout, Alison Turner, Mona Parmar, Holly Tovey, Ruth Riisnaes, Penny Flohr, Jesus Gil, David Waugh, Shaun Decordova, Anna Schlag, Bianca Calì, Andrea Alimonti, Johann S. de Bono

**Affiliations:** 1 The Institute of Cancer Research, London, UK; 2 The Royal Marsden NHS Foundation Trust, London, UK; 3 Northern Ireland Cancer Centre, Belfast, UK; 4 Patrick G Johnston Centre for Cancer Research, Queen’s University Belfast, Belfast, UK; 5 Oncology Institute of Southern Switzerland, Ente Ospedaliero Cantonale (EOC), Bellinzona, Switzerland; 6 Faculty of Biomedical Sciences, Università della Svizzera Italiana (USI), Lugano, Switzerland; 7 MRC London Institute of Medical Sciences (LMS), London, UK; 8 Institute of Clinical Sciences (ICS), Faculty of Medicine, Imperial College London, London, UK; 9 Centre for Cancer Biology, University of South Australia, Adelaide, South Australia Australia; 10 Institute of Oncology Research, Bellinzona, Switzerland; 11 Department of Health Sciences and Technology, Eidgenössische Technische Hochschule Zürich (ETH), Zurich, Switzerland; 12 Department of Medicine, Veneto Institute of Molecular Medicine, University of Padova, Padua, Italy

**Keywords:** Prostate cancer, Drug development

## Abstract

Inflammation is a hallmark of cancer^1^. In patients with cancer, peripheral blood myeloid expansion, indicated by a high neutrophil-to-lymphocyte ratio, associates with shorter survival and treatment resistance across malignancies and therapeutic modalities^2–5^. Whether myeloid inflammation drives progression of prostate cancer in humans remain unclear. Here we show that inhibition of myeloid chemotaxis can reduce tumour-elicited myeloid inflammation and reverse therapy resistance in a subset of patients with metastatic castration-resistant prostate cancer (CRPC). We show that a higher blood neutrophil-to-lymphocyte ratio reflects tumour myeloid infiltration and tumour expression of senescence-associated mRNA species, including those that encode myeloid-chemoattracting CXCR2 ligands. To determine whether myeloid cells fuel resistance to androgen receptor signalling inhibitors, and whether inhibiting CXCR2 to block myeloid chemotaxis reverses this, we conducted an investigator-initiated, proof-of-concept clinical trial of a CXCR2 inhibitor (AZD5069) plus enzalutamide in patients with metastatic CRPC that is resistant to androgen receptor signalling inhibitors. This combination was well tolerated without dose-limiting toxicity and it decreased circulating neutrophil levels, reduced intratumour CD11b^+^HLA-DR^lo^CD15^+^CD14^−^ myeloid cell infiltration and imparted durable clinical benefit with biochemical and radiological responses in a subset of patients with metastatic CRPC. This study provides clinical evidence that senescence-associated myeloid inflammation can fuel metastatic CRPC progression and resistance to androgen receptor blockade. Targeting myeloid chemotaxis merits broader evaluation in other cancers.

## Abstract (web summary)

A translational study demonstrates the role of myeloid inflammatory cells in driving disease progression and treatment resistance in prostate cancer and shows that these cells can be targeted therapeutically.

## Main

Inflammation is a hallmark of cancer^1^. Although T cell-mediated antitumour immunity has been harnessed for the management of some malignancies, myeloid inflammation, reflected by a high neutrophil-to-lymphocyte ratio (NLR) and neutrophilia, is associated with worse overall survival and therapeutic resistance across many malignancies^2–5^.

Metastatic prostate cancer is a common cause of male cancer mortality^6^. Existing treatments mainly target tumour cells directly. Treatments aimed at eliciting T cell-mediated immunity have, to date, shown insufficient clinical efficacy, possibly due to relatively low T cell infiltration in CRPC and the presence of immunosuppressive cues^7–9^. Prostate cancer is frequently infiltrated by myeloid inflammatory cells, including CD11b^+^HLA-DR^lo^CD15^+^CD14^−^ cells (also termed polymorphonuclear myeloid-derived suppressor cells (PMN-MDSCs) or tumour-associated neutrophils) as well as heterogeneous, alternatively differentiated myeloid cells^10–15^. Several groups have shown, using prostate cancer mouse models, that intratumour myeloid cells can drive paracrine oncogenic signalling, senescence evasion and immunosuppression^10–12,14–17^. This is supported by observations of increases in intratumour CD11b^+^HLA-DR^lo^CD15^+^CD14^−^ cells with progression from treatment-naive to metastatic CRPC (mCRPC)^10^. Moreover, overexpression of a myeloid-specific signature on whole-blood mRNA profiling and high NLR predict shorter overall survival in patients with mCRPC^2,3,18,19^. High NLR also associates with resistance to androgen receptor signalling inhibitors (ARSIs)^3^.

Preclinical studies indicate that recruitment of myeloid cells into prostate tumours is at least in part driven by tumour-derived chemokines binding to CXCR2 (refs. ^10,12,14,15^). CXCR2 ligands have been shown to be upregulated in response to tumour-suppressor loss and oncogenic activation^12,15,20–22^. Some of the mainstays of prostate cancer treatment—radiotherapy and androgen deprivation therapy—can also trigger upregulation of CXCR2 ligands, which constitute a part of the senescence-associated secretory phenotype (SASP)^10,12,14,23^. In models of different tumours, including prostate cancer, CXCR2 inhibition blocks the recruitment of tumour-infiltrating PMN-MDSCs leading to tumour suppression^10,12,14,24,25^. However, the extent to which this axis drives myeloid recruitment into the periphery and then into the tumour, and whether these cells contribute to human CRPC progression, are not clear. Critically, whether CXCR2 inhibition can overcome therapeutic resistance and confer clinical benefit to patients with mCRPC has not been evaluated. We reasoned that CXCR2 chemokines released by human prostate cancer cells sculpt systemic myeloid inflammation, and that targeting CXCR2 would decrease deleterious myeloid inflammation, reverse ARSI resistance and impart clinical benefit in some patients with mCRPC.

## NLR reflects tumour myeloid infiltration

**Fig. 1:**
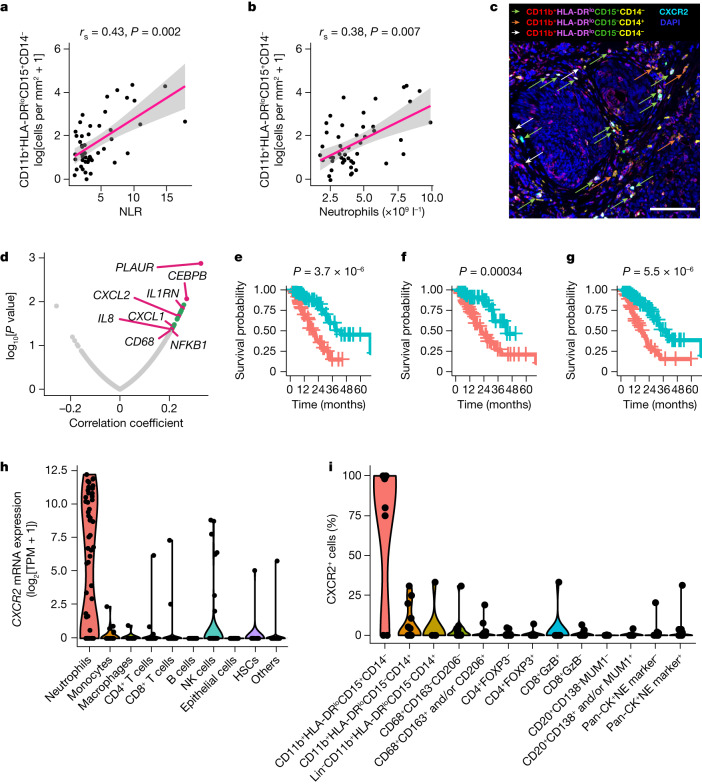
Prostate tumour cells generate CXCR2 chemokines associated with tumour and peripheral myeloid inflammation. **a**,**b**, Scatter plots of log-transformed intratumour CD11b^+^HLA-DR^lo^CD15^+^CD14^−^ cell density versus NLR (**a**) and neutrophil count (**b**) in patients with mCRPC (cohort 1, *n* = 48). Shown are estimated linear regression lines (pink) with 95% confidence intervals (grey), correlation coefficients, and *P* values from the two-sided Spearman’s rank correlation analyses. **c**, Micrograph showing a six-colour IF panel example of a human mCRPC biopsy stained for CD11b, HLA-DR, CD15, CD14 and CXCR2 and with DAPI, with arrows depicting different myeloid subsets. Scale bar, 100 μm. Entire slides were scanned and analysable slide areas were quantified for **a**,**b**. **d**, Volcano plot of the top 20 immune transcripts (green and pink) expressed in mCRPC biopsy bulk transcriptomes (RMH cohort, *n* = 95) that most positively associated with NLR. Pink, SASP genes and CXCR2 chemokines. **e**–**g**, Kaplan–Meier plots of overall survival from the time of CRPC biopsy based on gene expression of CXCL1 (**e**), CXCL2 (**f**) and CXCL8 (**g**) in CRPC bulk transcriptomes from the SU2C–PCF (*n* = 141) cohort. Gene expression cutoff was determined using the optimized Maxstat method. Blue line, low expression; red line, high expression. *P* values were calculated using the log-rank test. **h**, Violin plot of *CXCR2* mRNA expression from single-cell RNA-seq data from 15 advanced prostate cancer biopsies (14 patients). TPM, transcripts per million; NK, natural killer; HSCs, haematopoietic stem cells. **i**, Violin plots by proportion of intratumour immune cell and tumour cells staining for CXCR2 protein in human mCRPC biopsies (*n* = 14). NE, neuroendocrine. Source Data

**Extended Data Fig. 1:**
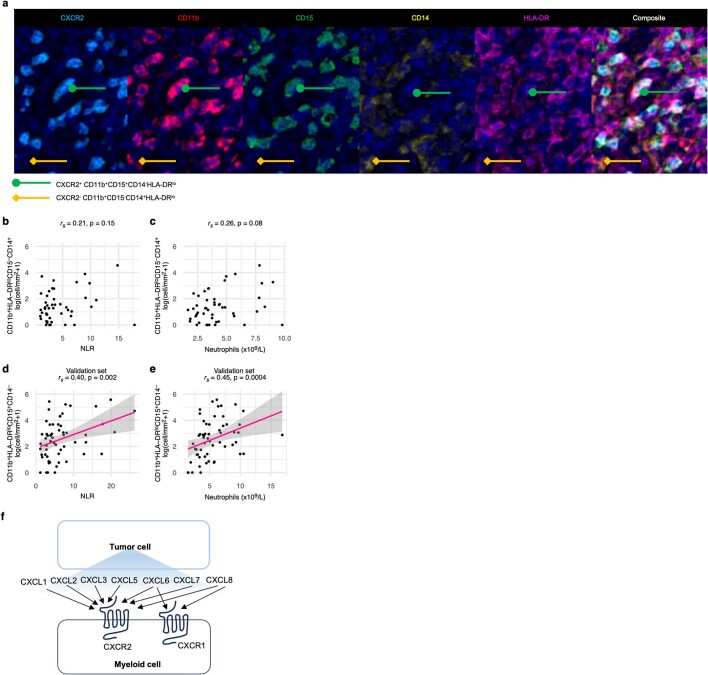
Associations between CRPC myeloid cell density and NLR. **a**, Example images from the six-colour IF panel for identifying myeloid cells (example of staining of appendix) of CXCR2, CD15, CD11b, CD14, HLA-DR, and DAPI. Green pointer: CD11b^+^HLA-DR^lo^CD15^+^CD14^−^, yellow arrow: CD11b^+^HLA-DR^lo^CD15^−^CD14^+^. Scale bar = 20 µm. **b**,**c**, Scatter plot of peripheral blood NLR (**d**) and neutrophil count (x10^9^/L) (**e**) versus log-transformed intratumor CD11b^+^HLA-DR^lo^CD15^−^CD14^+^ cell density (cells/mm^2^) in cohort 1 (n = 48). **d**,**e**, Scatter plot of peripheral blood NLR (**b**) and neutrophil count (x10^9^/L) (**c**) versus intratumor CD11b^+^HLA-DR^lo^CD15^+^CD14^−^ myeloid cell density (cells/mm^2^) in the validation cohort (n = 57). For **a**–**e**, correlation coefficients and p-values from the two-sided Spearman’s rank correlation analyses are shown. For **d**,**e**, estimated linear regression lines (pink) with 95% confidence interval (grey band). **f**, diagram of known CXCR1 and CXCR2 ligands. Source data are presented in the source data file. Source Data

As circulating and intratumour myeloid inflammation associate with worse prostate cancer outcomes^3,10^, we first evaluated associations between the circulating and tumour myeloid compartments. We analysed mCRPC biopsies from 48 patients (cohort 1) treated at 3 hospitals (Royal Marsden (RMH), Oncology Institute of Southern Switzerland and Belfast City Hospital). Most of these patients had previously received at least one ARSI (97.9%) and at least one taxane (95.8%; Supplementary Table 1). The density of myeloid inflammatory cells was quantified by six-colour immunofluorescence (IF) for CD11b, CD15, CD14, HLA-DR, DAPI (nuclear stain) and CXCR2 (Extended Data Fig. 1a). Intratumour CD11b^+^HLA-DR^lo^CD15^+^CD14^−^ myeloid cell density positively associated with contemporaneously collected peripheral blood NLR and neutrophil counts (Fig. 1a–c). There was no significant association between CD11b^+^HLA-DR^lo^CD15^−^CD14^+^ (also termed monocytic) myeloid cell density and peripheral blood NLR or neutrophil count (Extended Data Fig. 1b,c). We validated this positive association between CD11b^+^HLA-DR^lo^CD15^+^CD14^−^ myeloid cell density and peripheral blood NLR, as well as neutrophil count, in a further cohort of 57 patients with mCRPC treated at the RMH (validation cohort), where most patients had received at least one ARSI (91.2%) and at least one taxane (93.0%; Extended Data Fig. 1d,e and Supplementary Table 1). The positive associations between NLR, and circulating neutrophil counts, and tumour biopsy myeloid cell infiltration were statistically significant irrespective of the biopsy sites in both cohorts (Supplementary Table 2).

## CXCR2 ligands and myeloid inflammation

Given the association between peripheral and intratumour myeloid compartments, we reasoned that intratumour CD11b^+^HLA-DR^lo^CD15^+^CD14^−^ cell infiltration is driven by tumour-derived chemokines. First, we carried out transcriptome analyses associating peripheral blood NLR and neutrophil count with the expression of pan-immune genes (770 gene list from the nCounter PanCancer Immune Profiling Panel^26^) in bulk RNA-sequencing (RNA-seq) data from contemporaneously collected mCRPC biopsies from 95 patients treated at the RMH^27^ who had received at least one taxane and one ARSI. These mCRPC biopsy RNA-seq analyses showed that eight senescence- and myeloid-associated transcripts, including *CXCL1*, *CXCL2*, *CXCL8* (*IL8*), *IL1RN* (*IL1RA*), *CD68*, *PLAUR*, *NFKB1* and *CEBPB*, were among the top 20 genes most positively associated with NLR. CXCL1, CXCL2 and CXCL8 are ligands for CXCR2, implicated in the SASP and myeloid chemotaxis^21,28,29^. C/EBPβ and NF-κB have been shown to modulate the transcription of the CXCR2 ligands CXCL1, CXCL2 and CXCL8 (Fig. 1d and Extended Data Fig. 1f; refs. ^21,30–32^). Next, we determined whether the genes encoding these ligands associate with intratumour myeloid inflammation. The expression of *CXCL1*, *CXCL2*, *IL1RN* and *PLAUR* positively associated with two previously published myeloid gene signatures^10,33^ in the Stand Up To Cancer–Prostate Cancer Foundation (SU2C–PCF)^34^ and RMH CRPC RNA-seq datasets^27^. The associations between *CEBPB* (RMH) and *CXCL8* (SU2C–PCF) with both myeloid gene signatures were observed in one of the two datasets (Supplementary Table 3).

**Extended Data Fig. 2:**
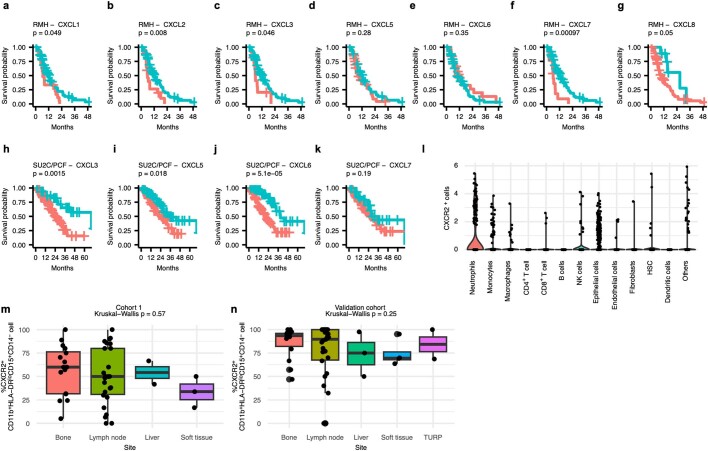
Clinical relevance of targeting the CXCR2 axis on myeloid cells in CRPC. **a**–**k**, Kaplan-Meier curves showing survival of mCRPC patients from the time of CRPC biopsy based on gene expression of CXCR2 chemokines in the RMH cohort (n = 94) (**a**–**g**) and SU2C/PCF cohort (n = 141) (**h**–**k**). Gene expression cut-offs were determined using the Maxstat method. p-values were calculated using the log-rank test. For **(a–k)**, blue line represents low expression, red line represents high expression. **l**, Violin plot of CXCR2 mRNA expression on single cells from single-cell RNASeq data from 11 primary prostate tumour samples. **m**,**n**, Proportion of CD11b^+^HLA-DR^lo^CD15^+^CD14^−^ myeloid cells expressing CXCR2 by biopsy site in cohort 1 (n = 48) (**m**) and validation cohort (n = 57) (**n**). For m, n, data are presented individually and as boxplots where the middle line is the median, the lower and upper hinges are the first and third quartiles, the upper whisker extends from the hinge to the largest value no further than 1.5 × inter-quartile range (IQR) from the hinge and the lower whisker extends from the hinge to the smallest value at most 1.5 × IQR from the hinge. Kruskal-Wallis p-values comparing the percentage of CXCR2^+^ myeloid cells across biopsy sites are shown. Source data for **a**–**k**, **m**,**n** are presented in the source data file. Source Data

To elucidate the clinical relevance of the CXCR2 axis in mCRPC, we carried out survival analyses for the CXCR2 ligands using these two RNA-seq datasets. Higher expression levels of *CXCL1*, *CXCL2*, *CXCL3*, *CXCL5*, *CXCL6* and *CXCL8* were negatively prognostic for overall survival from the time of CRPC biopsy in the SU2C–PCF cohorts. *CXCL1*, *CXCL2*, *CXCL3* and *CXCL7* were also negatively prognostic in the RMH cohort (Fig. 1e–g and Extended Data Fig. 2a–k). Overall, these data indicate that in patients with mCRPC, high peripheral blood NLR and neutrophilia associate with tumour cell expression of senescence-related transcripts, including those for CXCR2 chemokine ligands that can chemoattract myeloid cells into tumour, with the expression of these chemokines being negatively prognostic.

## Myeloid cells in CRPC express CXCR2

We next investigated CXCR2 expression on tumour and immune cells in human prostate cancer. We interrogated two publicly available single-cell RNA-seq datasets of CRPC samples from 14 patients^35^ and localized prostate cancer samples from 11 patients^36^. CXCR2 was specifically expressed and at high levels by cells clustered as neutrophils, with minimal tumour cell expression (Fig. 1h and Extended Data Fig. 2l). We next evaluated mCRPC biopsies from 14 patients with CRPC (a subset of cohort 1) who had received at least one ARSI to determine CXCR2 protein expression on tumour cells and immune cells of the following phenotypes: CD11b^+^HLA-DR^lo^CD15^+^CD14^−^ myeloid cells, CD11b^+^HLA-DR^lo^CD15^−^CD14^+^ myeloid cells, lineage-marker-negative (Lin^−^) CD11b^+^HLA-DR^lo^CD15^−^CD14^−^ myeloid cells, CD68^+^CD206^−^CD163^−^ macrophages, CD68^+^ macrophages expressing CD206 and/or CD163, CD4^+^FOXP3^+^ regulatory T cells, CD4^+^FOXP3^−^ T helper cells, T cells positive for CD8 and granzyme B (GzB), CD8^+^GzB^−^ T cells, CD20^+^CD138^−^MUM1^−^ B cells, CD20^+^ B cells expressing MUM1 and/or CD138, pan-CK^+^ cells without biomarkers associated with a neuroendocrine phenotype (synaptophysin (Syn), CD56 and chromogranin (CgA)) and pan-CK^+^ tumour cells expressing at least one of the biomarkers associated with a neuroendocrine phenotype (Supplementary Figs. 1 and 2).

Consistent with the single-cell RNA-seq results, the data on the expression of membranous CXCR2 protein showed that it was expressed by most CD11b^+^HLA-DR^lo^CD15^+^CD14^−^ myeloid cells, and to a lesser extent by other myeloid subsets, but was largely undetectable on non-myeloid cells (Fig. 1i). Interrogation of cohort 1 (*n* = 48 patients) and the validation cohort (*n *= 57 patients) also showed that CXCR2 was expressed by most CD11b^+^HLA-DR^lo^CD15^+^CD14^−^ cells in CRPC biopsies, and the frequency of expression did not differ significantly across metastatic sites (Extended Data Fig. 2m,n). The selective and frequent expression of CXCR2 by CD11b^+^HLA-DR^lo^CD15^+^CD14^−^ myeloid cells in CRPC biopsies makes it an attractive therapeutic target.

## Clinical trial of CXCR2 inhibitor plus ARSI

**Fig. 2:**
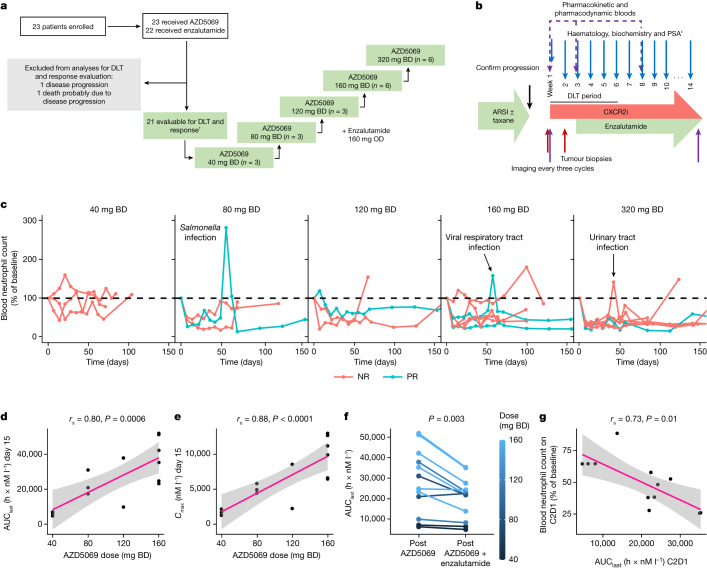
CXCR2 blockade leads to dose-dependent, on-target neutropaenia. **a**, Patient disposition per Consolidated Standards of Reporting Trials guidelines. ^†^Two patients were replaced per protocol after coming off study before completing the DLT period for a reason other than a DLT, and therefore were not evaluable for the primary endpoint or response. **b**, Clinical trial schema. Patients had confirmed disease progression on androgen deprivation therapy and at least one ARSI. Week count relative to the commencement of AZD5069 administration is shown. Cohorts 1–4 started AZD5069 2 weeks before enzalutamide; cohort 5 started drugs concurrently. ^‡^PSA test was carried out on day 1 of each cycle. **c**, By-patient, serial, peripheral blood neutrophil counts for each dose level of AZD5069. All available data points up to day 150 are shown. NR, patient classed as a non-responder; PR, patient classed as a partial responder. **d**, Scatterplot of AZD5069 dose versus AUC_last_ (h × nM l^−1^) for AZD5069 monotherapy on day 15 of AZD5069 administration at 40 to 160 mg BD (*n* = 14). **e**, Scatterplot of AZD5069 dose and peak concentration (*C*_max_ (nM l^−1^)) on day 15 of AZD5059 administration in patients treated with AZD5069 at 40 to 160 mg BD (*n* = 14). **f**, AZD5069 plasma concentration (AUC_last_ (h × nM l^−1^)) at steady state for AZD5069 monotherapy (after 14 days of monotherapy) versus combination therapy (after 28 days of combined administration of AZD5069 and enzalutamide; *n* = 12 pairs). Two-sided paired Wilcoxon signed-rank test *P* value is shown. Line colour indicates AZD5069 dose. **g**, Scatterplot of AZD5069 plasma concentration on cycle 2 day 1 (C2D1) (*x* axis) and  blood neutrophil count on C2D1 as a percentage of the value at baseline. For **d**,**e**,**g**, estimated linear regression lines (pink) with 95% confidence interval (grey band), and correlation coefficients and *P* values from the two-sided Spearman’s rank correlation analyses are shown.

We next administered a CXCR2 inhibitor (CXCR2i) to patients with mCRPC progressing after at least one ARSI in an investigator-initiated clinical trial, to generate proof-of-mechanism data showing that CXCR2i can reduce peripheral and tumour myeloid infiltration, as well as to pursue proof-of-concept evidence that inhibition of myeloid infiltration can reverse clinical ARSI resistance. This was a dose-finding, phase 1 trial of a selective CXCR2i, AZD5069 (40 mg twice daily (BD) to 320 mg BD), combined with enzalutamide (160 mg once daily (OD)) in patients with ARSI-resistant mCRPC using a 3 + 3 design. The primary trial objective was to evaluate the safety and tolerability of the combination. Key secondary objectives were to evaluate pharmacokinetic–pharmacodynamic parameters and antitumour activity. Administration of AZD5069 was commenced 2 weeks before that of enzalutamide in all but the 320 mg BD cohort to identify potential pharmacokinetic interactions (Fig. 2a,b). Between November 2017 and November 2022, we enrolled 23 patients with metastatic castration-resistant prostate adenocarcinoma (Extended Data Table 1). All patients were evaluable for safety, having received at least one dose of either study drug. Twenty-one patients were evaluable for dose-limiting toxicities (DLTs). Two patients were not evaluable for the primary endpoint and were replaced per protocol after coming off study for clinical progression and not toxicity after 1 and 6 days on trial before completing the DLT period.

We did not observe any DLT. The most common treatment-emergent adverse events were uncomplicated, dose-dependent, on-target neutropaenia (83%, grade ≥ 3: 48%), fatigue (30%, no grade ≥ 3), nausea (22%, no grade ≥ 3), anaemia (17%, grade ≥ 3: 4%), leukopaenia (13%, no grade ≥ 3), headache (13%, no grade ≥ 3), constipation (9%, no grade ≥ 3) and thrombocytopaenia (9%, no grade ≥ 3; Extended Data Table 2). Three patients had infections, deemed unrelated to the investigational agents by the safety review committee, and mounted appropriate, transient, neutrophilic responses, supporting the reversible mechanism of AZD5069 in inhibiting myeloid chemotaxis, rather than impairing marrow function or causing leukocyte destruction. Infections (viral respiratory tract infection and *Salmonella* gastroenteritis) were self-limiting in two patients; urinary tract infection in the third patient with urinary tract outflow obstruction resolved after oral antibiotics (Fig. 2c). There was no permanent treatment discontinuation due to treatment-emergent adverse events. One patient (80 mg BD dose) underwent permanent dose reduction of AZD5069 for grade 4 neutropaenia. One patient died after 6 days on study probably owing to rapidly progressing disease, but no postmortem was carried out so relatedness to the investigational agents could not be unequivocally excluded.

## CXCR2i reduced myeloid cell counts

**Extended Data Fig. 3:**
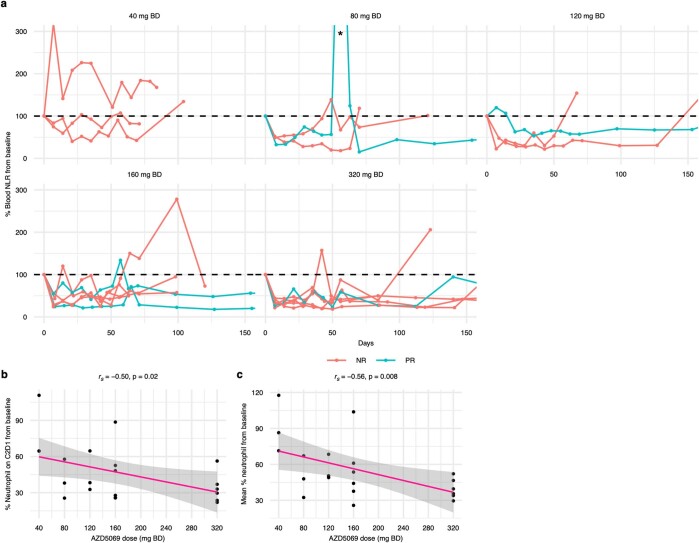
CXCR2 inhibition led to dose-dependent decreases in blood neutrophils. **a**, By-patient, serial, peripheral blood NLR for each dose level of AZD5069. Aqua line: responder, Pink line: non-responder. *Outlier with NLR peak of 789% from baseline on cycle 2 day 15 in the setting of salmonella infection. **b**, Scatterplot of AZD5069 dose administered twice daily versus percentage peripheral blood neutrophil counts on cycle 2 day 1 compared with baseline. **c**, Scatterplot showing of AZD5069 dose versus mean percentage blood neutrophil count on treatment compared with baseline. For **b**,**c**, estimated linear regression lines (pink) with 95% confidence interval (grey band), and correlation coefficients and p-values from the two-sided Spearman’s rank correlation analyses are shown.

AZD5069 exposure represented by the area under the concentration-time curve from time zero to time of the last quantifiable concentration (AUC_last_) and maximum concentration (*C*_max_) at steady state increased in a dose-dependent manner (Fig. 2d,e), but AZD5069 exposure (AUC_last_) decreased after the addition of enzalutamide, particularly at the higher dose levels, probably owing to enzalutamide increasing hepatic clearance of AZ5069 by CYP3A4 induction (Fig. 2f). Nonetheless, AZD5069 decreased blood neutrophil counts and NLRs in a dose-dependent manner (Fig. 2c and Extended Data Fig. 3). The association between the degree of neutropaenia and AZD5069 exposure on cycle 2 day 1 also confirms this pharmacokinetic-pharmacodynamic relationship (Fig. 2g). To elucidate the impact of CXCR2i on intratumour myeloid cell infiltration and tumour immune landscape, we carried out hyperplex IF for immune and tumour cell markers on paired tumour biopsies obtained from the same disease site within 1 week prior to and approximately 2 weeks after starting treatment. Biopsies were taken from 18 patients (17 pairs). Thirteen pairs of biopsies were analysable by hyperplex IF as four pairs of samples did not contain sufficient tumour, were too blood stained, or were crushed (Supplementary Table 4).

**Fig. 3:**
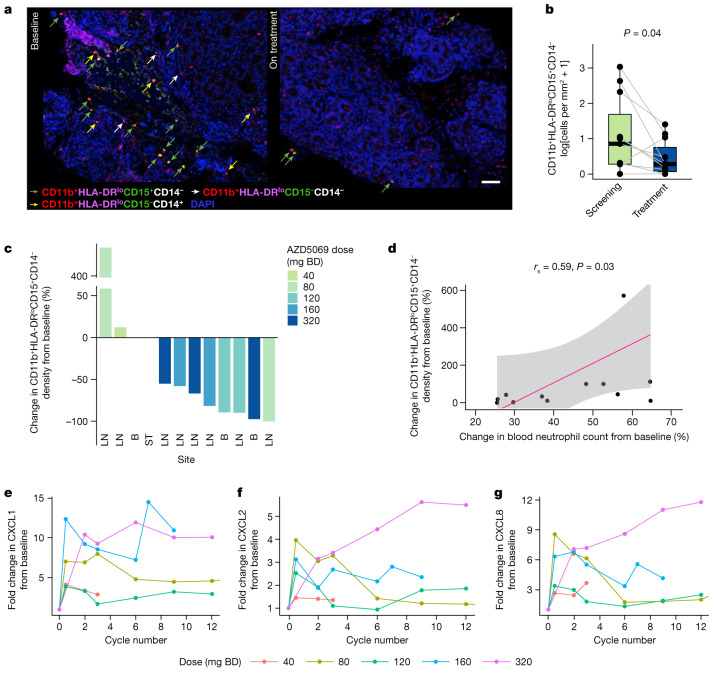
CXCR2 blockade reduces myeloid infiltration in some patients with CRPC. **a**, Example of a pair of CRPC biopsies showing myeloid cell changes before and after starting treatment. Green arrow: CD11b^+^HLA-DR^lo^CD15^+^CD14^−^ cells; yellow arrow: CD11b^+^HLA-DR^lo^CD15^−^CD14^+^ cells; white arrows: CD11b^+^HLA-DR^lo^CD15^−^CD14^−^ cells. Nuclei were counterstained with DAPI. Scale bar, 100 µm. **b**, Comparison of CD11b^+^HLA-DR^lo^CD15^+^CD14^−^ myeloid cell densities (log-transformed cells per mm^2^) in mCRPC biopsies pre-treatment and on treatment in patients with blood neutrophil decrease of >30% (>40 mg BD dose levels; *n* = 11 pairs). Data are presented individually and as boxplots in which the middle horizontal line is the median, the lower and upper hinges are the first and third quartiles, and the upper and lower whiskers extend from the hinge to the minimum and maximum values. Grey lines link results from paired same-patient samples. Two-sided paired Wilcoxon signed-rank test *P* value is shown. **c**, Waterfall plot of percentage change in the density of CD11b^+^HLA-DR^lo^CD15^+^CD14^−^ myeloid cells in mCRPC biopsies before and after CXCR2i. The biopsy sites are annotated as LN for lymph node, B for bone, and ST for soft tissue. **d**, Scatter plot of the percentage of blood neutrophils on cycle 2 day 1 compared with baseline and the percentage of intratumour CD11b^+^HLA-DR^lo^CD15^+^CD14^−^ cell density after CXCR2i compared with baseline (for **c**,**d**, *n* = 13, but note that one outlier for which myeloid cell density increased from a baseline of zero (fold change = infinity) is not shown on the graph). An estimated linear regression line (pink) with 95% confidence interval (grey band), and correlation coefficients and *P* values from the two-sided Spearman’s rank correlation analyses, are shown. **e**–**g**, By-dose-level, mean fold change in circulating CXCL1 (*n* = 14 patients), CXCL2 (*n* = 20 patients) and CXCL8 (*n* = 20 patients) levels on study compared with baseline, pre-treatment levels. Data for patients from whom samples were not collected, or whose samples failed quality control for enzyme-linked immunosorbent assay, are not included. Line colour indicates AZD5069 dose.

**Extended Data Fig. 4:**
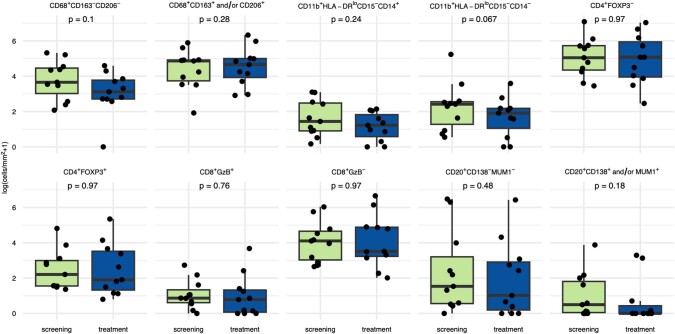
Immune cell densities in CRPC biopsies with CXCR2 inhibition. Intratumor immune cells densities (log-transformed density (cells/mm^2^)) in mCRPC biopsies taken during pre- (green) and on-treatment (blue), evaluating the immune cell subsets shown in all patients where on-target neutropenia (>30% mean decrease in neutrophils) was observed (n = 11 pairs). Data are represented by immune cell subset, individually, and as boxplots where the middle line is the median, the lower and upper hinges are the first and third quartiles, the upper whisker extends from the hinge to the largest value no further than 1.5 × interquartile range (IQR) from the hinge and the lower whisker extends from the hinge to the smallest value at most 1.5 × IQR from the hinge. Two-sided paired Wilcoxon signed-rank test p*-*values are shown.

CXCR2i reduced the density of CD11b^+^HLA-DR^lo^CD15^+^CD14^−^ myeloid cells in CRPC biopsies of most patients treated with AZD5069 >40 mg BD, doses sufficient to achieve on-target reduction in blood neutrophil counts of at least 30% (Fig. 3a–c). As low HLA-DR is not always used when phenotyping granulocytic myeloid cells, we also examined the impact of CXCR2i on CD11b^+^CD15^+^CD14^−^ myeloid cells and observed a consistent change. Notably, the change in CD11b^+^HLA-DR^lo^CD15^+^CD14^−^ myeloid cell density reflected AZD5069 exposure and blood neutropaenia (Fig. 3d). CXCR2i did not significantly alter the density of other immune cells, which rarely express CXCR2 (Extended Data Fig. 4).

**Extended Data Fig. 5:**
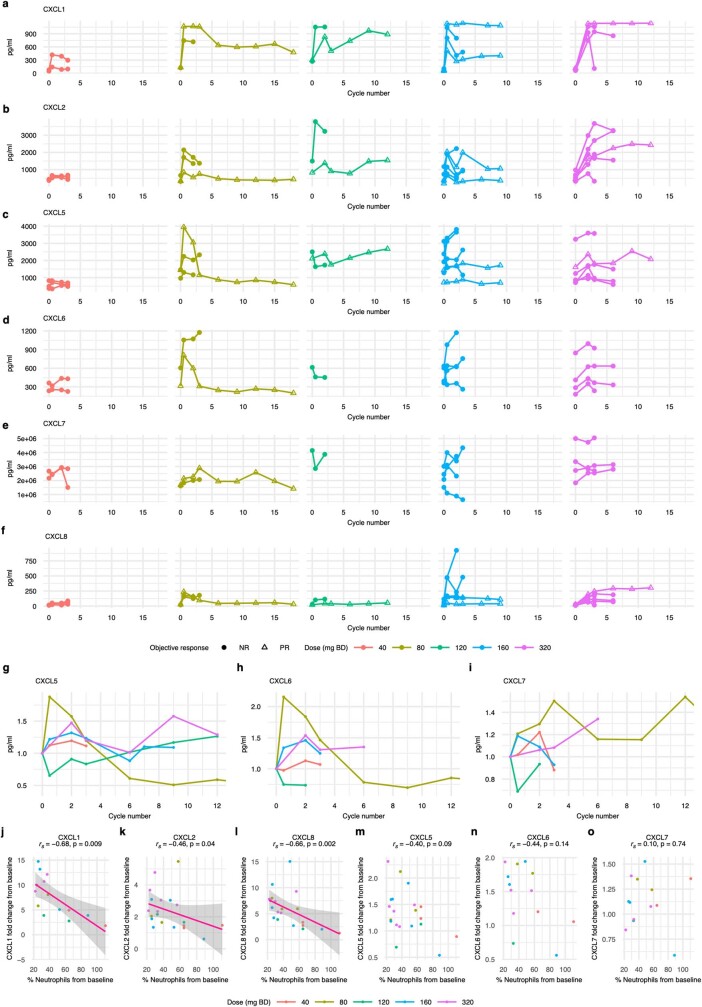
Blood CXCR1 and CXCR2 cytokines after CXCR2i and enzalutamide. **a**–**f**, Individual patient circulating levels of CXCL1 (n = 14), CXCL2 (n = 20), CXCL5 (n = 20), CXCL6 (n = 13), CXCL7 (n = 13), CXCL8 (n = 20) by dose level. Missing patients did not have samples collected or samples that failed quality control. **g**,**h**,**i**, By-dose level, mean fold change in circulating CXCL5 (**g**, n = 20), CXCL6 (**h**, n = 13), and CXCL7 (**i**, n = 13) levels compared with baseline over time. Samples were taken at baseline, on day 1 of each cycle and day 15 of the first cycle. **j**,**k**,**l**, Scatterplot of CXCL1 (**j**, n = 14), CXCL2 (**k**, n = 20), CXCL8 (**l**, n = 20) fold change from baseline versus percent blood neutrophil count from baseline on cycle 2 day 1. Estimated linear regression lines (pink) with 95% confidence interval (grey band), and correlation coefficients and p-values from the two-sided Spearman’s rank correlation analyses are shown. **m**,**n**,**o**, Scatterplot of CXCL5 (n = 20) (**m**), CXCL6 (n = 13) (**n**), CXCL7 (n = 13) (**o**) fold change from baseline versus percent blood neutrophil count from baseline on cycle 2 day 1. Correlation coefficient and p-value from the two-sided Spearman’s rank correlation test are shown. Colour of the lines and dots represent AZD5069 dose. NR = non-responder, PR = partial responder. BD = twice daily.

To determine whether CXCR2i led to compensatory ligand upregulation, we serially measured the levels of circulating CXCL1, CXCL2, CXCL5, CXCL6, CXCL7 and CXCL8. CXCL3 was not measured. Notably, the levels of CXCL1, CXCL2 and CXCL8, the only CXCR2 ligands whose gene expression correlated with NLR in our initial analysis (Fig. 1d), increased in a dose-dependent manner after treatment. The degree of neutropaenia on cycle 2 day 1 correlated with the degree of CXCL1, CXCL2 and CXCL8 upregulation at the same time point (Fig. 3e–g and Extended Data Fig. 5). CXCL5, CXCL6 and CXCL7 levels did not consistently increase after CXCR2i or associate with neutropaenia (Extended Data Fig. 5). These findings indicate that CXCR2i can affect granulocytic myeloid cell infiltration; however, compensatory upregulation of ligands that can bind to CXCR1 and/or CXCR2 is a potential resistance mechanism.

## CXCR2i can reverse CRPC ARSI resistance

**Fig. 4:**
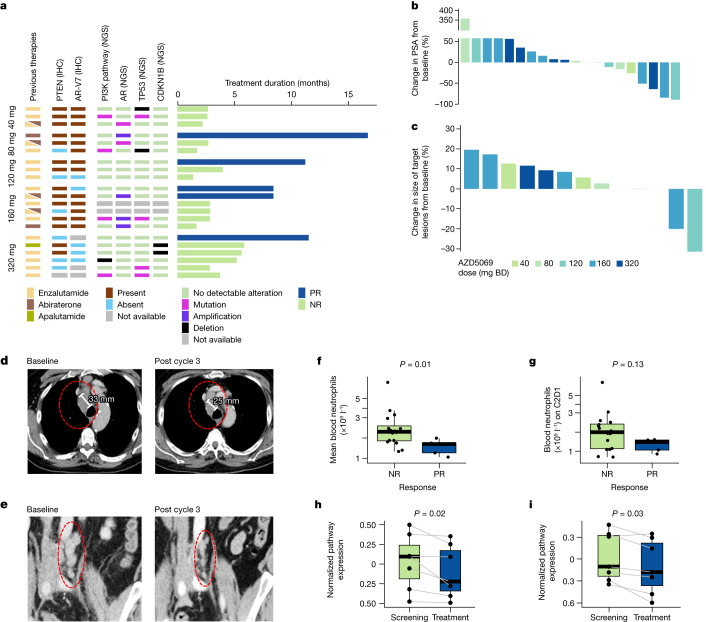
CXCR blockade can reverse ARSI resistance in patients with mCRPC. **a**, Treatment duration of response-evaluable patients grouped by AZD5069 dose (*n* = 21). Blue, patient classed as a partial responder; green, patient classed as a non-responder. Legend and coloured tiles indicate previous ARSI: enzalutamide (yellow), abiraterone (brown), apalutamide (dark green); AR-V7 protein status: nuclear Histo-score (HS) ≥ 10 (brown), nuclear HS < 10 (blue); PTEN protein status: nuclear or cytoplasmic HS ≥ 10 (brown), nuclear and cytoplasmic HS < 10 (blue), not available (grey); *TP53*, *AR* and *PTEN–PI3K* pathway genes, and *CDKN1B* genomic aberration status: no detectable alteration (green), pathogenic mutation (magenta), amplification (purple) and deletion (black) in baseline biopsies or cell-free DNA. **b**, Best PSA responses (*n* = 20). One patient was not evaluable for PSA response owing to early clinical disease progression. **c**, Best radiologic response in patients with measurable disease (*n* = 13). **d**,**e**, Example computerized tomography scan images of measurable disease taken pre-treatment and on treatment in two patients classed as partial responders treated at AZD5069 160 mg BD (**d**) and AZD5069 120 mg BD (**e**). White bars in **d** demarcate the short axis of a lymph node metastasis. **f**,**g**, Boxplots of mean blood neutrophil counts on treatment (**f**) and cycle 2 day 1 (C2D1) neutrophil counts (**g**) in patients classed as partial responders (*n* = 5) versus those classed as non-responders (*n* = 16). In **f**,**g**, data are presented individually and as boxplots in which the middle horizontal line is the median, the lower and upper hinges are the first and third quartiles, and the whisker extends from the hinge to the largest and smallest values no further than 1.5 × interquartile range (IQR) from the hinge. Two-sided Mann–Whitney *U*-test *P* values are shown. **h**,**i**, Expression of AR activity (**h**) and AR-V7 mRNA signatures (**i**) in same-patient pre- and on-treatment tumour biopsies (*n* = 7 pairs) with myeloid count decrease. In **h**,**i**, data are presented individually and as boxplots for which the middle line is the median, the lower and upper hinges are the first and third quartiles, and the upper and lower whiskers extend from the hinge to the maximum and minimum values. Grey lines link same-patient, paired samples. Two-sided paired Wilcoxon signed-rank test *P* values are shown.

Five (24%) of 21 patients had an objective partial response using the prespecified response criteria of: ≥30% decrease in measurable disease according to the Response Evaluation Criteria in Solid Tumors v.1.1, prostate-specific antigen (PSA) decline ≥50% confirmed ≥4 weeks later, and/or conversion of circulating tumour cell (CTC) count from ≥5 per 7.5 ml of blood at baseline to <5 per 7.5 ml of blood at nadir (Fig. 4a–c). Patients needed to be on treatment for at least 12 weeks to be considered a responder. Thirteen patients had assessable measurable disease by Response Evaluation Criteria in Solid Tumors v.1.1. Eight patients had a baseline CTC count of ≥5 per 7.5 ml of blood. All patients classed as partial responders had radiologic progression-free survival of more than 6 months. Four patients classed as partial responders had confirmed disease progression by Prostate Cancer Working Group 2 criteria on enzalutamide, and one had progressed on abiraterone alone, prior to treatment with AZD5069. Three of four patients classed as partial responders, for whom pre-treatment tissue biopsy was available for androgen receptor splice variant 7 (AR-V7) immunohistochemistry (IHC) including the patient who received only abiraterone previously, had tumour expressing AR-V7 protein. AR-V7 is a reported biomarker of enzalutamide resistance^37^.

**Extended Data Fig. 6:**
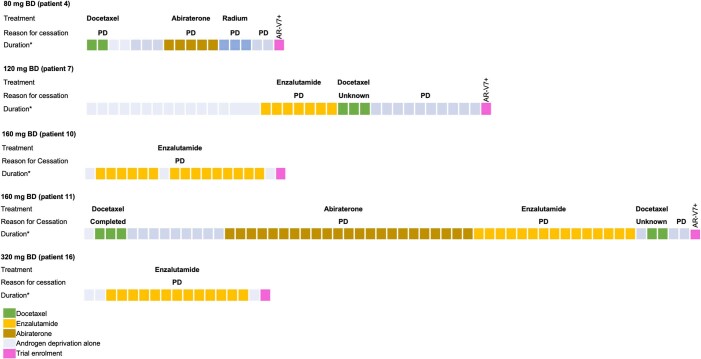
Prior treatment history of responders. Systemic therapies administered after CRPC diagnosis in responders. Patients are ordered as per Fig. 4a. Each rectangle represents a two-month interval. All patients received androgen deprivation therapy throughout this period. Dose represents AZD5069 dose. AR-V7+ indicates the presence of AR-V7 protein expression in the pre-treatment CRPC biopsy. BD = twice daily.

The patient classed as a partial responder treated with AZD5069 at 320 mg BD had Gleason 9 mCRPC (AR-V7 IHC not available) with progressing high-volume bone metastases on enzalutamide before trial entry (Extended Data Fig. 6). PSA doubling time was 1 month. We observed a 64% PSA reduction (129 ng ml^−1^ to 47 ng ml^−1^) after four cycles. Whole-body magnetic resonance imaging showed an increase in apparent diffusion coefficient in the bone metastases, indicating response, after three cycles, but the patient developed new bone metastases after 8 months (Fig. 4a,b).

**Extended Data Fig. 7:**
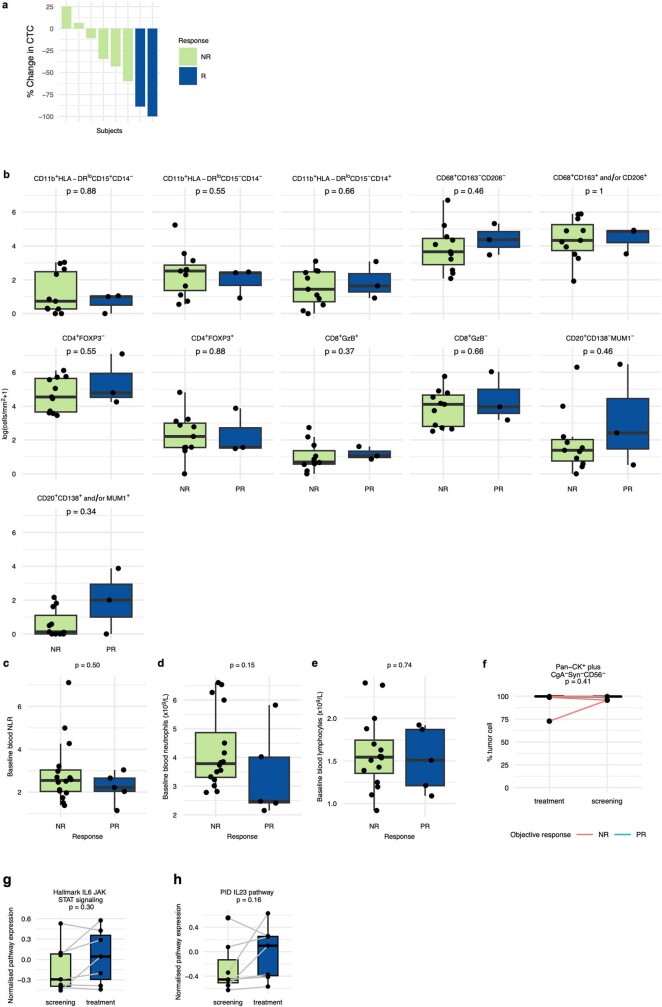
Biomarker and bulk RNAseq analyses. **a**, Waterfall plot showing maximum percentage CTC count decline from baseline in patients with CTC count ≥5 cells/7.5 ml at baseline. The two responding (R) patients with CTC count conversion from ≥5/7.5 ml of blood to <5 cells/7.5 ml of blood are shown in blue; patients also need to be on treatment for at least three cycles to be considered a responder. Non-responders (NR) are in green. **b**, Baseline immune cell densities (log-transformed (cells/mm^2^)) in mCRPC biopsies of responders (n = 3) versus non-responders at baseline (n = 11). Missing patients did not have analysable paired tumour sample for analysis by IF assays. **c**,**d**,**e**, Baseline blood NLR (**c**), neutrophil (x10^9^/L) (**d**), and lymphocyte (x10^9^/L) (**e**) in responders (n = 5) versus non-responders (n = 16). In **b**–**e**, data are presented individually and as boxplots where the middle horizontal line is the median, the lower and upper hinges are the first and third quartiles, the upper whisker extend from the hinge to the largest value no further than 1.5 × interquartile range (IQR) from the hinge and the lower whisker extends from the hinge to the smallest value at most 1.5 × IQR from the hinge. Two-sided Mann-Whitney U test p-values are shown. **f**, Chromogranin (CgA)^−^synaptophysin (Syn)^−^CD56^−^ pan-CK^+^ cells as a proportion of all pan-CK^+^ cells in paired tumour biopsies (n = 11 pairs). Lines link paired samples and colour denotes response. NR = non-responder (red), PR = partial responder (green). Two-sided paired Wilcoxon signed-rank test p-value is shown. **g**,**h**, Boxplot of gene expression of the Hallmark IL-6-JAK2-STAT3 signalling gene signature (**g**) and PID IL-23 signature (**h**) in RNA profiling data from paired CRPC biopsies with CD11b^+^HLA-DR^lo^CD15^+^CD14^−^ cell decrease (n = 7 pairs). In **g**,**h**, data are presented individually and with boxplots where the middle line is the median, the lower and upper hinges are the first and third quartiles, and the upper and lower whiskers extend from the hinge to the largest value no further than 1.5 × IQR from the hinge and the lower whisker extends from the hinge to the smallest value at most 1.5 × IQR from the hinge. Lines link paired, same-patient samples. Two-sided paired Wilcoxon signed-rank test p-values are shown.

Two patients classed as partial responders were treated with AZD5069 at 160 mg BD. The first had de novo metastatic prostate cancer with bone and lymph node metastases (Gleason score unknown) that progressed after docetaxel in the hormone-sensitive setting, and then progressed on abiraterone, enzalutamide, and docetaxel sequentially in the castration-resistant setting prior to trial entry (Extended Data Fig. 6). Baseline biopsy was AR-V7^+^. Before trial entry, PSA doubled every 2 months, and a 41% increase in nodal disease occurred over 4 months. PSA fell by 84% (344 ng ml^−1^ to 55 ng ml^−1^) and nodal disease decreased by 20% at nadir (3–4 months; Fig. 4c,d). Radiologic progression-free survival was 8 months. The second patient classed as a responder had de novo metastatic prostate cancer (Gleason 9) with bone-only metastases, received docetaxel for hormone-sensitive prostate cancer, and then progressed in multiple bone metastases while on enzalutamide shortly before trial entry. Baseline biopsy was AR-V7^−^. PSA doubling time was 4 months. CTC count initially increased from 11 per 7.5 ml of blood to a peak of 39 per 7.5 ml of blood during cycle 1, and then transiently decreased to 0 per 7.5 ml of blood during cycle 4. The patient had a radiologic progression-free survival of 8 months; however, there was no PSA decrease (Fig. 4a, Extended Data Fig. 7a and Supplementary Table 5).

The patient classed as a partial responder treated with AZD5069 at 120 mg BD had mCRPC (Gleason 8) that progressed on enzalutamide, administered for 14 months, 2 years before trial entry and then after docetaxel both in the castration-resistant setting. The baseline biopsy was AR-V7^+^. At trial entry, the patient had progressing bone metastases, and new supra and infra-diaphragmatic lymphadenopathy compressing the inferior vena cava, pelvic veins and lymphatics, causing lower limb and abdominal wall lymphoedema. PSA fell by 89% (98 ng ml^−1^ to 11 ng ml^−1^) after 5 cycles and measurable disease reduced by 31%, with clinical improvement of lymphoedema; radiologic and clinical responses lasting 11 months (Fig. 4e).

The patient classed as a partial responder treated with AZD5069 at 80 mg BD had a relatively high drug exposure, with AUC_last_ and *C*_max_ comparable to those of patients treated at 160 mg BD, and grade 4 neutropaenia necessitating dose reduction. This patient had Gleason 8 mCRPC with bone metastases, which progressed on docetaxel, abiraterone and radium-223. The patient had new bone metastases and a PSA doubling time of 4 months. CTC count decreased from 9 per 7.5 ml at baseline to 1 per 7.5 ml of blood at cycle 2; CTC conversion was maintained for seven cycles. The PSA response criterion was not met and there was no measurable disease. This patient remained on trial for 16 months. On the basis of the response, toxicity and pharmacokinetic–pharmacodynamic associations, we recommended both the 160 mg BD and 320 mg BD dose levels for phase 2 evaluation; however, the expansion study was terminated owing to discontinued production of AZD5069.

To explore potential biomarkers of response, we compared the baseline molecular and immunologic profiles of patients classed as partial responders with those of patients classed as non-responders. This analysis showed no significant difference in baseline NLR, neutrophil count, lymphocyte count or the density of tumour myeloid, T and B cells, although only three patients classed as responders had biopsies for IF (Extended Data Fig. 7b–e). Patients classed as partial responders had lower on-treatment mean blood neutrophil counts compared with patients classed as non-responders (Fig. 4f,g). Varying degrees of increasing neutrophil counts were observed in patients classed as responders at progression (Fig. 2c and Extended Data Fig. 3).

We also examined CXCR2 expression on tumour cells, although this was rare in this cohort selected for adenocarcinoma tumour histology. In two of three of the patients classed as partial responders, CXCR2 protein was not detected on tumour cells at baseline. In one patient classed as a partial responder, CXCR2 was detected on 2.1% of pan-CK^+^CgA^−^Syn^−^CD56^−^ cells and 1.8% of pan-CK^+^ cells expressing at least one of the neuroendocrine phenotype markers. There was no significant change in the proportion of neuroendocrine phenotype marker-positive tumour cells overall or in the patients classed as partial responders (Extended Data Fig. 7f).

Given previous reports of CXCR2 chemokine upregulation in PTEN-deleted prostate tumours^10,11,20,23^, we interrogated pre-treatment tumour PTEN protein expression (5 patients classed as partial responders, 15 patients classed as non-responders) and pathway gene alteration using a previously described next-generation sequencing (NGS) panel^38^ (5 patients classed as partial responders, 14 patients classed as non-responders). One patient classed as a responder had PTEN protein loss and eight patients classed as non-responders had PTEN protein loss or pathway-activating genomic alterations (Fig. 4a). We next evaluated genomic aberrations in cell cycle and senescence machinery genes (*CDKN1A*, *CDKN1B*, *CDKN2A*, *CDKN2B*, *CDKN2D, RB1* and *TP53*) using the aforementioned NGS panel on the basis of the hypothesis that myeloid-targeted approaches may depend on cell arrest and associated senescence programs in tumour cells for response. Alterations of these genes were not detected in the patients classed as partial responders, whereas 8/14 patients classed as non-responders had tumours with deep deletion or pathogenic mutation of *TP53* and *CDKN1B* (p27; Supplementary Table 6). These data indicate that the benefit of CXCR2 inhibition in mCRPC is not limited to tumours in which the PTEN–PI3K pathway is activated, but whether deleterious alterations in *TP53* or cyclin-dependent kinase (CDK) inhibitors confer resistance warrants further evaluation.

## Impact of myeloid chemotaxis inhibition on tumour

To explore how CXCR2 inhibition of myeloid cell chemotaxis impacted mCRPC biology, we carried out capture-based RNA profiling (HTG EdgeSeq) from paired pre- and on-treatment mCRPC biopsies (seven pairs of biopsies had sufficient tumour content) from the patients with reduced intratumour CD11b^+^HLA-DR^lo^CD15^+^CD14^−^ myeloid cell density after treatment. We focused on the following: AR activity signatures^39^; AR-V7 signatures^40^; the IL-23 pathway signature^41^; and IL-6–JAK2–STAT3 signalling signatures^42^. CXCR2 blockade associated with downregulated AR activity and AR-V7 signatures, but did not associate with significant changes in the other pathways examined (Fig. 4h,i and Extended Data Fig. 7g,h). The degree of AR activity and AR-V7 signature downregulation was the most marked in the patient classed as a responder. AR activity and AR-V7 signature expression did not change significantly in patients in whom the density of intratumour CD11b^+^HLA-DR^lo^CD15^−^CD14^−^ cells did not decrease. Overall, these data support previous reports of myeloid infiltrates impacting oncogenic AR signalling.

## Discussion

Myeloid inflammatory cells have been reported to play a key role in cancer therapy resistance and progression in preclinical models^10–12,14,15^, but this has not been demonstrated in patients, with the drivers of intratumour myeloid infiltration remaining incompletely understood. This translational study demonstrates the relationship between peripheral and intratumour granulocytic myeloid compartments in patients with mCRPC, and indicates that a tumour-elicited and senescence-associated secretome drives CXCR2-expressing myeloid cell chemotaxis into tumour to fuel disease progression and treatment resistance. This is in keeping with preclinical studies showing that tumour cells, whether triggered by oncogenic signalling or treatment, sometimes in the context of senescence, can upregulate ligands that chemoattract myeloid cells^10,12,14,15,21^. The associations between NLR and neutrophil counts and high intratumour CD11b^+^HLA-DR^lo^CD15^+^CD14^−^ myeloid infiltration suggest that this simple blood test reflects a high level of intratumour myeloid cell infiltration into mCRPC. To our knowledge, this is the first clinical trial to show that CXCR2 blockade can reverse ARSI resistance to impart durable, clinical benefit in a subset of patients with CRPC. These data support the pathogenic role of myeloid inflammation in human prostate cancer and may explain why, across many cancer types, neutrophilia predicts worse outcome.

The responses in this study are probably mediated by tumour-extrinsic mechanisms given that few tumour cells expressed significant levels of CXCR2 protein, with the recruited patients having adenocarcinoma histology for which CXCR2 expression is rare^43^. The durable responses were unlikely to be due to re-response to enzalutamide monotherapy because patients either were progressing on enzalutamide immediately before trial entry or, if there was a treatment gap between trial entry and last ARSI, had tumours that expressed AR-V7, which associates with ARSI resistance^37^. The association between the degree of neutropaenia following CXCR2 blockade—a pharmacodynamic biomarker correlating with intratumour myeloid count decreases—and response suggests that myeloid cell clearance is critical to response. The downregulation of AR activity and AR-V7 signatures in tumours in which CD11b^+^HLA-DR^lo^CD15^−^CD14^−^ cell density decreased after treatment with CXCR2i is consistent with preclinical work showing that PMN-MDSCs drive AR signalling through IL-23, although PMN-MDSCs can make other paracrine factors including IL-6 and NRG1 (refs. ^10,17^). This array of ligands and metabolites can, however, impact several oncogenic and immunomodulatory pathways^10,12,14,44^, and the observed downregulation of AR target genes could be indirect.

Several patients classed as non-responders had deleterious alterations of *TP53* and *CDKN1B*, alterations not detected in patients classed as partial responders; p53 and p27 loss may allow tumours to bypass cell cycle arrest^45,46^. Larger studies are needed to explore whether the wild-type status of tumour-suppressor genes associated with senescence including those expressing TP53 and CDK inhibitors predicts response to this therapeutic strategy. Elucidating resistance to this therapeutic strategy is also critically important; this may be due to incomplete intratumour myeloid cell clearance, or myeloid cell re-accumulation due to compensatory chemokine ligand upregulation, as was indicated by serial assessments of CXCR2 chemokines in our study, with some of the upregulated chemokines potentially binding not only CXCR2 but also CXCR1 (ref. ^47^). Understanding of these interactions, and the post-translational modifications that impact binding affinity and signalling, is now needed to elucidate the necessity of targeting multiple chemokine receptors to maximally block myeloid chemotaxis into tumours^48,49^.

In conclusion, we provide evidence that CXCR2 blockade inhibits senescence-associated, tumour-elicited myeloid inflammation, with this blockade reversing ARSI resistance to confer durable antitumour activity in a subset of patients with mCRPC. Myeloid targeting warrants evaluation in larger cohorts of patients with prostate cancer and in earlier disease stages. Due consideration should be given to potential resistance mechanisms including redundant and compensatory chemokine–receptor interactions. The importance of this SASP biology to resistance to other senescence-inducing treatments also needs to be evaluated across treatments and cancer types.

## Methods

### Trial participants

We conducted an international, phase 1, multi-centre, single-arm, open-label trial (ClinicalTrials.gov identifier: NCT03177187, EudraCT: 2016-003141-28) at three centres in Europe (RMH (UK), Belfast City Hospital (UK), Oncology Institute of Southern Switzerland (Switzerland)). Eligible patients were consenting patients aged ≥18 years, who had mCRPC with histologically confirmed prostate adenocarcinoma histology, documented cancer progression at the time of trial entry by Response Evaluation Criteria in Solid Tumors (v.1.1) and/or Prostate Cancer Working Group 2 criteria, and PSA of ≥10 ng ml^−1^ at screening. Patients needed to have disease progression while on androgen deprivation therapy (orchiectomy and/or ongoing luteinizing hormone-releasing hormone (LHRH) agonist treatment), and confirmed disease progression on at least one of enzalutamide, darolutamide, apalutamide or abiraterone, having received at least 12 weeks of treatment of each. Before treatment with other prostate cancer treatments was permitted: patients needed to be undergoing androgen deprivation therapy with serum testosterone <50 ng dl^−1^ (<2.0 nM); patients needed to be Eastern Cooperative Oncology Group performance status of 0 or 1, and have adequate haematologic, renal, liver, and coagulation function; patients also needed to be willing to undergo pre- and on-treatment mCRPC biopsies, when safe and feasible.

Patients were excluded if their prostate cancer was predominantly small cell or neuroendocrine differentiated. Patients were excluded if they had any of the following: surgery, chemotherapy or other anticancer therapy (with the exception of an ARSI and gonadotropin hormone-releasing hormone analogue therapy) within 4 weeks before trial entry; limited field radiotherapy within 2 weeks or wide-field radiotherapy within 4 weeks of trial entry; hypoaldosteronism or hypopituitarism; history of seizures or predisposing factors to seizures; known central nervous system metastasis; malabsorption syndrome; prolonged QT interval > 470 milliseconds; clinically important rhythm, conduction, or ECG abnormality; predisposing factor to QT prolongation; coronary intervention, myocardial infarction, angina, or congestive cardiac failure (New York Heart Association ≥grade 2) in the past 6 months; uncontrolled hypotension or hypertension; clinically important history of liver disease (for example, Child–Pugh B or C, viral or other hepatitis, current alcohol abuse, or cirrhosis); malignancy other than prostate cancer within the past 5 years; immunocompromising disorder; thromboembolic event within the past 12 months; active or uncontrolled autoimmune disorder requiring steroids. Full eligibility criteria are described in the study protocol (Supplementary Information).

### Study oversight

The study was conducted in accordance with the provisions of the Declaration of Helsinki and Good Clinical Practice guidelines. Regulatory approvals were obtained from the Medicines Healthcare products Regulatory Agency, Swiss Swissmedic and the institutional research ethics committee (REC; the London-Surrey Borders REC (UK sites) and Comitato Etico Cantonale Bellinzona (Switzerland)). Written informed consent was obtained from all participants. No participant compensation was provided. A safety review committee evaluated the safety and tolerability at regular intervals after recruitment of three patients to a schedule. All protocol amendments were approved by the trial sponsor, Medicines Healthcare products Regulatory Agency, Swissmedic and local UK and Swiss RECs. The study was sponsored and monitored by The Institute of Cancer Research (ICR), UK. The study was registered on ClinicalTrials.gov before commencement.

### Study design

In this investigator-initiated, international, open-label, phase 1 study, we evaluated five escalating doses of orally administered AZD5069 (40 mg BD, 80 mg BD, 120 mg BD, 160 mg BD and 320 mg BD) in combination with standard, fixed-dose, orally administered enzalutamide (160 mg OD), over 28-day cycles, until disease progression, intolerance or withdrawal of consent. During the first cycle (42 days), AZD5069 was commenced 2 weeks before enzalutamide in the first four cohorts, primarily to evaluate any pharmacokinetic interactions between the two drugs. The starting dose of AZD5069 was determined on the basis of preclinical pharmacokinetics results as well as pharmacodynamic, pharmacokinetic and safety results from previous studies in humans in which the main side effect observed was dose-dependent, reversible neutropaenia in blood, without significant increase in infection rate in patients with airway disease, or healthy individuals.

The dose-escalation phase used a rule-based 3 + 3 design, with an initial three patients enrolled per dose level. If none of the first three patients experienced a DLT, dose escalation proceeded to the next dose level. If one instance of DLT was observed in three patients, up to six patients were treated at that dose level. If fewer than two of six patients at any dose level experienced a DLT, dose escalation continued to the next level. If at least two out of up to six patients experienced a DLT, dose escalation stopped and this dose level was defined as the maximum administered dose. Once this maximum administered dose was defined, the maximum tolerated dose (MTD) was confirmed at the dose level below the maximum administered dose. At least six evaluable patients were required to establish the MTD at a specific dose level. Only doses at which no more than one of six patients had a DLT could be defined as the MTD. Four potential dose-escalation cohorts with increasing AZD5069 doses (40 mg BD, 80 mg BD, 120 mg BD and 160 mg BD) were planned initially. The study protocol was amended on 16 December 2020 to explore a fifth dose level of AZD5069, 320 mg BD, with the option to de-escalate to 240 mg BD (dose level 4B) if dose level five was intolerable, and the study drugs were administered concurrently. This amendment occurred after previous dose levels were deemed safe and because a decrease in AZD5069 exposure was observed after adding enzalutamide. Intra-patient dose escalation was not permitted. Start of dosing between the first and second patient enrolled to each dose level was staggered by 1 week. Once the MTD was determined in the phase 1 study, the recommended phase 2 dose was determined on the basis of available data, including but not limited to safety and response.

### Study procedures

#### Safety monitoring

Adverse events were monitored at least weekly during cycles 1 and 2, and then once per cycle from cycle 3 onwards, and graded using the National Cancer Institute Common Terminology Criteria for Adverse Events (NCI-CTCAE) v.4.0, until 28 days after the discontinuation of study treatment or until the resolution of a persistent drug-related adverse event. DLTs were defined as described in the study protocol. Notably, febrile neutropaenia (neutrophil count <0.5 × 10^9^ l^−1^ and fever >38.3 °C or fever ≥38 °C for >1 h), infection with grade 4 neutropaenia, and grade 4 neutropaenia for 7 days or more were defined as DLTs. Prophylaxis with growth factor support or antipyretics were not permitted. Investigators determined whether an adverse event was related to the study drugs. If a patient experienced clinically significant and/or unacceptable toxicity, including a DLT, not attributable to the disease or disease-related processes, dosing was interrupted or the dose was reduced and supportive therapy was administered as required. If the toxicity resolved or reverted to ≤CTCAE v.4.0 grade 1 within 14 days of onset, treatment with the combination of enzalutamide and AZD5069 could be restarted following agreement with the sponsor.

In patients who experienced grade 3 or greater toxicity related to enzalutamide in the opinion of the investigator that could not be ameliorated by the use of adequate medical intervention, enzalutamide dosing was interrupted until the toxicity improved to grade 1 or lower severity, and a dose reduction to enzalutamide 120 mg daily was permitted. No further dose reduction for enzalutamide was permitted. During dose interruption due to an adverse event deemed related to AZD5069, treatment could be recommenced at the same dose if the toxicity resolved or reverted to CTCAE v.4.0 grade ≤1 in less than eight days; however, if the toxicity took 8 to 14 days to resolve or revert to grade ≤1, AZD5069 could be restarted at a lower dose (one dose level lower than the last dose received) as per specification in the protocol. Enzalutamide dose remained unchanged. For all other events, if the toxicity did not resolve to CTCAE v.4.0 grade ≤1 after more than 14 days, then treatment was discontinued and the patient was observed until resolution of the toxicity. Patients were taken off study if either study drug had to be permanently discontinued.

#### Tumour response assessment

Radiologic tumour responses were measured by computerized tomography of the thorax, abdomen and pelvis, bone scan, and, where indicated, whole-body magnetic resonance imaging, at baseline, once every 3 cycles, and then at the end of treatment if this was more than 8 weeks since the last scan. PSA and CTC count were measured at baseline, and then on day 1 of every cycle, and at the end of treatment. CTC counts were analysed using previously described methods^38^.

#### Pharmacokinetic and pharmacodynamic studies

Blood samples for pharmacokinetic analyses of AZD5069 and enzalutamide were collected and analysed from all patients in the first four dose levels at the commencement of AZD5069 (cycle 1 day −14), cycle 1 day 1 (after 2 weeks of AZD5069 monotherapy) and cycle 2 day 1 (after 4 weeks of AZD5069 and enzalutamide combination). Specific pharmacokinetic collection time points are listed in the study protocol. Pharmacokinetic parameters were calculated using non-compartmental analysis (Phoenix v.8.1, Certara). Fresh tumour needle core biopsies from matched disease sites (lymph node, bone and soft tissue) were collected at baseline (within 1 week of treatment commencement) and approximately 2 weeks after starting AZD5069 in patients when this was deemed safe and feasible. Tumour biopsies were obtained from 18 patients under computerized tomography or ultrasound guidance by an experienced interventional radiologist (N.T.). Three patients did not undergo biopsies because this was not deemed safe or the patient declined. One patient had only a baseline tumour biopsy. Two patients underwent on-treatment biopsies after 4 weeks. Immediately after biopsy, tumour samples were immersed in 10% neutral-buffered formalin for 24 hours. Samples were processed through paraffin wax for histologic examination. Three 3 μm sections of formalin-fixed paraffin-embedded (FFPE) tumour biopsy were stained with haematoxylin and eosin to confirm the presence of tumour by a pathologist (B.G.). Tumour samples with insufficient tumour content or significant crushed artefact were excluded from analyses (B.G.). Baseline FFPE samples were analysed by IHC, and pre- and post-treatment FFPE biopsies were analysed by IF and targeted RNA profiling using HTG EdgeSeq. All samples that failed initial analyses by IF were restained at least once. A list of tumour samples and their methods of analyses are detailed in Supplementary Table 4.

### Outcomes

The primary endpoints were to identify the DLTs, estimate the MTD and identify the recommended phase 2 dose of AZD5069 administered in combination with enzalutamide at 160 mg OD. Secondary endpoints were:(i) the rate of objective response, with objective response defined as confirmed soft tissue objective response by Response Evaluation Criteria in Solid Tumors v.1.1 in those with measurable disease, and/or PSA decline ≥50% confirmed 4 weeks or later, and/or CTC count conversion from ≥5 per 7.5 ml of blood at baseline to <5 per 7.5 ml of blood at nadir; (ii) pharmacokinetic parameters, including maximum concentration, area under the concentration–time curve; and (iii) pharmacodynamic changes including identifying patients whose blood NLR, neutrophil, and intratumour myeloid cell density decrease. Exploratory endpoints included blood cytokine levels and evaluation of tumour molecular profile on response.

### Patient samples for myeloid cell translational studies

The association between myeloid cell densities and NLR was evaluated in two cohorts (Supplementary Table 1). Cohort 1 consisted of 48 mCRPC biopsies from patients treated at ICR/RMH, Oncology Institute of Southern Switzerland and Belfast City Hospital between 2012 and 2021. All patients provided informed consent, and enrolled onto institutional protocols approved by the local RECs (REC reference: 04/Q0801/60, 11/LO/2019). The validation cohort consisted of 57 mCRPC biopsies from patients treated at ICR/RMH between 2012 and 2016 under institutional protocols approved by the local REC (REC references: 04/Q0801/60, 2017-01002 CE TI 3237). Full blood counts were carried out using routine automated haematology analysers. NLR was defined as the quotient of the absolute peripheral blood neutrophil count divided by the absolute blood lymphocyte count. For comparisons of peripheral blood NLR with intratumour myeloid cell density, blood counts collected on the day of the biopsy, or when this was not available, within 7 days preceding the biopsy were used. Human biological samples were sourced ethically and their research use was in accordance with the terms of the informed consent provided. Studies of CXCR2 expression on immune cells and tumour cells consisted of 14 patients treated at the RMH who underwent mCRPC biopsies under a research protocol approved by The RMH REC (REC reference: 04/Q0801/60) providing consent for these analyses.

### IF and IHC

#### Antibody validation

Antibodies against CXCR2, FOXP3, MUM1, CD163, CD68, HLA-DR, CD4, CD38, CD206, CD8 and GzB were validated by western blot and/or IHC comparing detection of protein expression in cells treated with either non-targeting control siRNA or ON-TARGETplus pooled siRNA against the target gene (Dharmacon) or using positive and negative control cell lines. Cells were authenticated by STR profiling and tested for mycoplasma (Venor GeM Mycoplasma Detection Kit, Minerva Biolabs). Markers were validated for appropriate tissue localization on immunohistochemical staining of relevant positive and negative tissue controls and reviewed by a certified pathologist (B.G.). Validation for PTEN, CD4, CD8, FOXP3, CD11b, CD15, CD14, CD138, CD20, Syn, CgA and AR-V7 was also previously described^13,17,50,51^. IHC was carried out on FFPE tissue sections using an automated staining platform (Bond RX, Leica Biosystems). Bone biopsies were decalcified using pH 7 EDTA for 48 h at 37 °C. Once validated for target sensitivity and specificity, the antibodies were further optimized for IHC, multiplex IF and hyperplex IF using methods described below. The full list of antibodies, working dilutions and incubation times is in Supplementary Tables 7 and 8.

#### Hyperplex IF assay

FFPE CRPC biopsies were stained using a hyperplex IF assay. For paired samples, the pre- and on-treatment biopsies from each patient, along with the positive and negative controls (tonsil, ovarian cancer, appendix, HeLa and LNCaP cell line pellet), were placed on the same slide to control for any technical variability in staining intensity and allow for comparison of the pre- and on-treatment biopsies. Samples from the tissue microarrays had been stained previously using orthogonal methods (IF and/or IHC) for confirmation. Standard operating procedures were implemented to control for known factors that can impact IF staining intensity, including the use of antibodies with the same lot number, minimization of freeze–thaw of antibodies, and controlling for the temperature of the experiment. Automated hyperplex IF staining and imaging was carried out on the COMET platform (Lunaphore Technologies). Slides underwent iterative staining and imaging, followed by elution of the primary and secondary antibodies^52^.

FFPE tissue sections of 3 μm in thickness were baked in an oven for 60 min at 60 °C, followed by deparaffinization in xylene and rehydration in a series of ethanol solutions of decreasing concentrations. Next, tissue sections were fixed in 10% neutral-buffered formalin solution (No. BAF-0010-05A, CellPath) for 20 min at room temperature. Antigen retrieval was achieved by heating the slides in heat-induced epitope retrieval buffer H pH 9 (No. TA-999-DHBH, Epredia, Shandon Diagnostics) in the PT Module (No. A80400011, Thermo Fisher Scientific) for 60 min at 102 °C. Subsequently, slides were rinsed and stored in Multistaining Buffer (BU06, Lunaphore Technologies) until use.

The hyperplex IF protocol template was generated using the COMET Control Software (v.0.70.0.1, Lunaphore Technologies), and reagents were loaded onto the device to carry out the sequential IF (seqIF) protocol^52^. Secondary antibodies were used as a mix of two species’ complementary antibodies plus DAPI, Alexa Fluor Plus 647 goat anti-rabbit (No. A32733, 1:400 dilution, Thermo Scientific) and Alexa Fluor Plus 555 goat anti-mouse (No. A32727, 1:200 dilution, Thermo Scientific) diluted in Intercept T20 (TBS) antibody diluent (No. 927-65001, LI-COR Biosciences). Nuclear signal was detected with DAPI (No. 62248, dilution 1:1,000, Thermo Fisher Scientific) by dynamic incubation of 2 min. Primary antibodies were diluted in multistaining buffer (BU06, Lunaphore Technologies). For each cycle, the following exposure times were used: DAPI 80 ms, TRITC 400 ms, Cy5 200 ms. The elution step lasted 2 min for each cycle and was carried out with elution buffer (BU07-L, Lunaphore Technologies) at 37 °C. The quenching step lasted for 30 seconds and was carried out with quenching buffer (BU08-L, Lunaphore Technologies). The imaging step was carried out with imaging buffer (BU09, Lunaphore Technologies). The seqIF protocol in COMET resulted in a multi-stack ome.tiff file in which the imaging outputs from each cycle are stitched and aligned. COMET ome.tiff contains a DAPI image, intrinsic tissue autofluorescence in TRITC and Cy5 channels, and a single fluorescent layer per marker.

Elution efficiency and epitope stability of each biomarker were assessed separately through several rounds of staining, elution and imaging on positive control tissue. Antibody titration was carried out to identify the best antibody dilution and incubation time. The staining sequence was optimized through an iterative process using several positive and negative FFPE controls (appendix, tonsil, ovarian cancer and prostate cancer), cell lines (PC3, LNCaP and HeLa) and a patient-derived xenograft with a neuroendocrine phenotype (CP142)^17^. Images were reviewed by a pathologist (B.G.) and used to determine the final marker permutation (Supplementary Table 7 and Supplementary Figs. 1 and 2).

#### Six-colour IF

Six-colour OPAL-based sequential IF staining was carried out on the Bond RX automated staining platform (Leica Biosystems). FFPE tissue sections of 3 µm underwent heat-induced epitope retrieval with epitope retrieval solution 2 (pH 9.0; No. AR9640, ER2, Leica Biosystems) followed by endogenous peroxidase blocking (Novocastra Peroxidase Block, No. RE7157, Leica Biosystems) for 10 min. Nonspecific antibody binding was blocked using OPAL antibody diluent/block (ARD1001EA, Akoya Biosciences) for 10 min. Primary antibodies against CXCR2, CD15, CD11b, CD14 and HLA-DR; Supplementary Table 8) were sequentially incubated for 30 min followed by detection with the Novolink Max Polymer Detection System (RE7280-K, Leica Biosystems). IF signals for CXCR2, CD15, CD11b, CD14 and HLA-DR were visualized using TSA coumarin (NEL703001KT, Akoya Biosciences), OPAL 520 (NEL820001KT, Akoya Biosciences), OPAL 570 (NEL820001KT, Akoya Biosciences), OPAL 650 (FP1496001KT, Akoya Biosciences) and OPAL 780 (FP1501001KT, Akoya Biosciences), respectively, and counterstained with spectral DAPI. Slides were scanned using the VS200 Research Slide Scanner (Olympus).

#### AI-assisted image analyses

The hyperplex and six-colour IF assay images were reviewed by a certified pathologist (B.G.) and histopathologists (M.C., A.F., I.F.). Images were analysed using Halo software (Indica Labs). Tissue segmentation was carried out using a supervised machine learning algorithm to recognize prostate cancer foci and surrounding stroma. Cell segmentation was achieved with nuclear DAPI counterstain and tumour-infiltrating immune cells were phenotypically characterized by cell surface marker. We identified CD11b^+^HLA-DR^lo^CD15^+^CD14^−^ and CD11b^+^HLA-DR^lo^CD15^−^CD14^+^ cells using a supervised machine learning algorithm trained by a pathologist (B.G.) as previously described^13^.

For the hyperplex IF panel, a threshold for positivity for each marker used for cell phenotyping was set by the pathologist by referencing positive and negative control tissue or cell line pellets stained on the same slide. The same thresholds were applied to the entire slide. Manual curation and comparison with controls was essential because differences in tissue type and quality can impact the intensity of different antibodies differently, although all phenotypic markers showed excellent signal-to-noise ratio (>15). Using these thresholds, Halo software (Indica Labs) was used to analyse each cell for the marker to provide single-cell-level binary readouts for phenotypic markers of interest. A Boolean gating strategy to identify cell types of interest on the basis of the intensity and specificity of markers was established (Supplementary Fig. 2).

### Next-generation sequencing

Targeted NGS using a 113-gene panel was carried out on 16 available pre-treatment tumour biopsies, and 3 pre-treatment cell-free DNA samples, extracted from 20 ml of plasma collected in Streck tubes. NGS was carried out using previously described methods^38,53^. Libraries were constructed from 40 ng of cell-free DNA using a customized GeneRead DNAseq Mix-n-Match v.2 panel (Qiagen) and sequenced on the MiSeq Sequencer (Illumina). The somatic variant calls were manually inspected in the Integrative Genomics Viewer (v.2.16.1, Broad Institute). The sensitivity of NGS results from cell-free DNA analyses was assessed by carrying this assay out on three additional samples from the AZD5069 320 mg BD dose level for which NGS on tumour biopsy was also carried out and we confirmed that all pathogenic alterations detected in the tumour biopsy were also found in the cell-free DNA.

### RNA profiling of FFPE tumour biopsies

RNA profiling of FFPE tumour biopsies collected before and after starting AZD5069 was carried out by HTG EdgeSeq (HTG Molecular Diagnostics) using the HTG human transcriptome panel containing 19,616 nuclease protection probes (NPPs), including 19,398 gene target-specific 50-nucleotide probes, 100 negative control probes, 92 probes for RNA controls established by the External RNA Control Consortium, 22 probes that measure gDNA and 4 positive control probes. The assay was carried out with a minimum of 11 mm^2^ of FFPE tumour micro-dissected sections. The sections were lysed according to the manufacturer’s instructions, and added to a 96-well plate on the HTG EdgeSeq Processor (HTG Molecular Diagnostics) on which a quantitative nuclease protection assay was carried out. The addition of DNA nuclease protection probes (NPPs) was automated and they were allowed to hybridize for 16 hours to the target mRNAs. The excess non-hybridized DNA probes and non-hybridized mRNA were removed by S1 digestion leaving only NPPs hybridized to mRNA. This produced a 1:1 ratio of DNA detection probes to mRNA targets present in the sample. Libraries were constructed by adding sequencing indices and molecular barcodes to the NPPs through polymerase chain reactions (PCR). After clean-up and quantification by quantitative PCR using the kit KAPA Library Quantification Kit Illumina platforms, the libraries were pooled and sequenced on the NextSeq 500 using a High Throughput 75-cycle v.2.5 kit (Illumina). FASTQ files were generated using BCL2FATSQ v.2.0 and raw count data were generated using HTG EdgeSeq Parser Software (v.5.3, HTG Molecular Diagnostics). Data were analysed using the HTG EdgeSeq Reveal analysis software. Several quality control metrics were carried out: QC0 (insufficient sample quantity or poor sample quality) with positive control probes >4% reads was marked as a failure; QC1 (insufficient read depth) with total aligned reads <7 million per sample was marked as a failure; QC2 (high background signal) with median log_10_ negative control probes >2 was marked as a failure; QC3 (incomplete digestion of gDNA by DNase) with median log_10_ gDNA control probes >1 was marked as a failure. Samples failing any of the quality controls were removed from the subsequent analysis. Differential gene expression between pre- and on-treatment samples was carried out using the HTG EdgeSeq Reveal DESeq2 analysis pipeline and R Software (v.4.2.3).

### Circulating cytokine analyses

Serum samples were collected at baseline, on day 1 of every cycle, and on cycle 1 day 15. CXCL2, CXCL5, CXCL6, CXCL7 and CXCL8 were measured in patient serum (diluted 1:2 except for CXCL7, which was diluted 1:200), then analysed using R&D Systems Luminex discovery assays using the Luminex 200 and interpolated using a five-parameter logistic curve fit. CXCL1 was measured in neat patient serum using the R&D Systems human GROα Quantikine ELISA using the Perkin Elmer Envision 2103 Multilabel plate reader and interpolated using linear regression. The Luminex and ELISA assays were validated to good clinical practice compliance by The ICR and included quality control samples of serum, unspiked or spiked with recombinant protein standard, from healthy volunteer serum in every analytical run. A list of the ELISA reagents is provided in Supplementary Table 9.

### Bioinformatics

#### mCRPC transcriptome analysis

A total of 159 mCRPC transcriptomes generated by the SU2C–PCF Prostate Cancer Dream Team^34^ were downloaded and reanalysed. Only samples that had library preparation using polyA+ RNA isolation were used (that is, samples with library preparation carried out by capture methods were excluded). A total of 141 mCRPC transcriptomes had associated survival data available for survival analyses. A separate 95 mCRPC transcriptomes from patients treated at RMH/ICR were analysed^27^; 94 mCRPC transcriptomes were used for the survival analyses as survival data were not available from 1 patient. The SU2C–PCF transcriptomes were aligned to the human reference genome (GRCh37/hg19) using TopHat2 (v.2.0.7). Gene expression as fragments per kilobase of transcript per million mapped reads (FPKM) was calculated using Cufflinks (v.2.2.1). Unbiased interrogation of pan-immune genes^26^ present in the RMH/ICR bulk RNA-seq datasets in relation to NLR was carried out. MDSC signatures were adapted from previously published signatures^10,33^. Associations were analysed using the two-sided Spearman’s rank correlation test.

#### mCRPC biopsy RNA profiling

For mCRPC tumour biopsy RNA profiling, HTG EdgeSeq data were processed with the EdgeSeq processor and included multiple steps (parsing, quality control and normalization) with default settings. The normalized counts were transformed to log_2_[counts per million], which was used for downstream analysis. Gene set variation analysis (GSVA, R package GSVA v.1.4) was used for molecular signature analysis.

#### Single-cell transcriptomic analysis

Single-cell transcriptomic data from 15 mCRPC samples from 14 patients (https://www.nature.com/articles/s41591-021-01244-6)^35^ and single-cell transcriptomic data from 11 patients with localized prostate cancer (https://www.nature.com/articles/s41467-021-27322-4)^36^ were downloaded. Data were loaded into R Software (v.4.1.3). The raw counts from the localized prostate cancer data were log normalized. Both datasets were processed with Seurat (v.4.3.0) and underwent scaling, clustering, dimensional reduction and cell type assignment with SingleR (v.1.8.1) using the Blueprint ENCODE reference dataset from the celldex (v.1.4.0) library.

### Statistics

Sample size was chosen pragmatically. According to the rule-based 3 + 3 design, which guided dose-escalation decisions, the cohort size was three patients and skipping of dose levels was not allowed. Patients who completed the DLT period or experienced a DLT during the DLT period were considered part of the evaluable population. Patients who received at least one dose of study drug were considered part of the safety population. To be evaluable for response, the patient must have met the eligibility criteria, received at least three cycles of trial medication, and have had baseline assessment of disease. Comparisons of baseline characteristics between patients classed as responders and those classed as non-responders were carried out using the two-sided Mann–Whitney *U*-test. Comparisons of paired pharmacokinetic and pharmacodynamic parameters were carried out using the two-sided paired Wilcoxon signed-rank test. Safety variables and pharmacokinetic and pharmacodynamic endpoints were summarized descriptively.

Immune cell densities and continuous gene expression data are presented descriptively as individual values, as well as violin plots or boxplots with median and interquartile range. All analysable areas on the entire slide were analysed. The two-sided Spearman’s rank correlation test was used to estimate associations between continuous variables, two-sided Mann–Whitney *U*-test was used to test for differences between unpaired groups, and the two-sided paired Wilcoxon signed-rank test was used to test for differences between paired samples. The Kruskal–Wallis test was used to compare myeloid cell densities across multiple disease sites. Multivariable linear regressions were carried out to determine whether associations between NLR or neutrophil count and myeloid cell densities were impacted by biopsy site. The Maxstat method^54,55^ determined gene expression cutoffs for survival analyses. Overall survival and progression-free survival were estimated using the Kaplan–Meier method. Between-group comparisons of survival curves were carried out using the log-rank test. Hazard ratios with 95% confidence intervals were calculated using Cox regression. All *P* values ≤ 0.05 were considered significant. Bonferroni correction was applied to adjust for multiplicity in the context of multiple hypothesis testing of myeloid gene signatures but not for associations between NLR and immune genes for which the aim of the analyses was to identify the most highly ranked immune genes associating with NLR. Circulating cytokine levels are presented descriptively. Statistical analysis was carried out using R software (v.4.2.2) and according to the statistical analysis plan for trial-related analyses.

### Reporting summary

Further information on research design is available in the Nature Portfolio Reporting Summary linked to this article.

## Online content

Any methods, additional references, Nature Portfolio reporting summaries, source data, extended data, supplementary information, acknowledgements, peer review information; details of author contributions and competing interests; and statements of data and code availability are available at 10.1038/s41586-023-06696-z.

### Supplementary information

Supplementary Information Supplementary Tables 1–9, Figs. 1 and 2 and the study protocol.

Reporting Summary

### Source data

Source Data Fig. 1 and Source Data Extended Data Figs. 1 and 2.

## Figures and Tables

**Extended Data Table 1:**
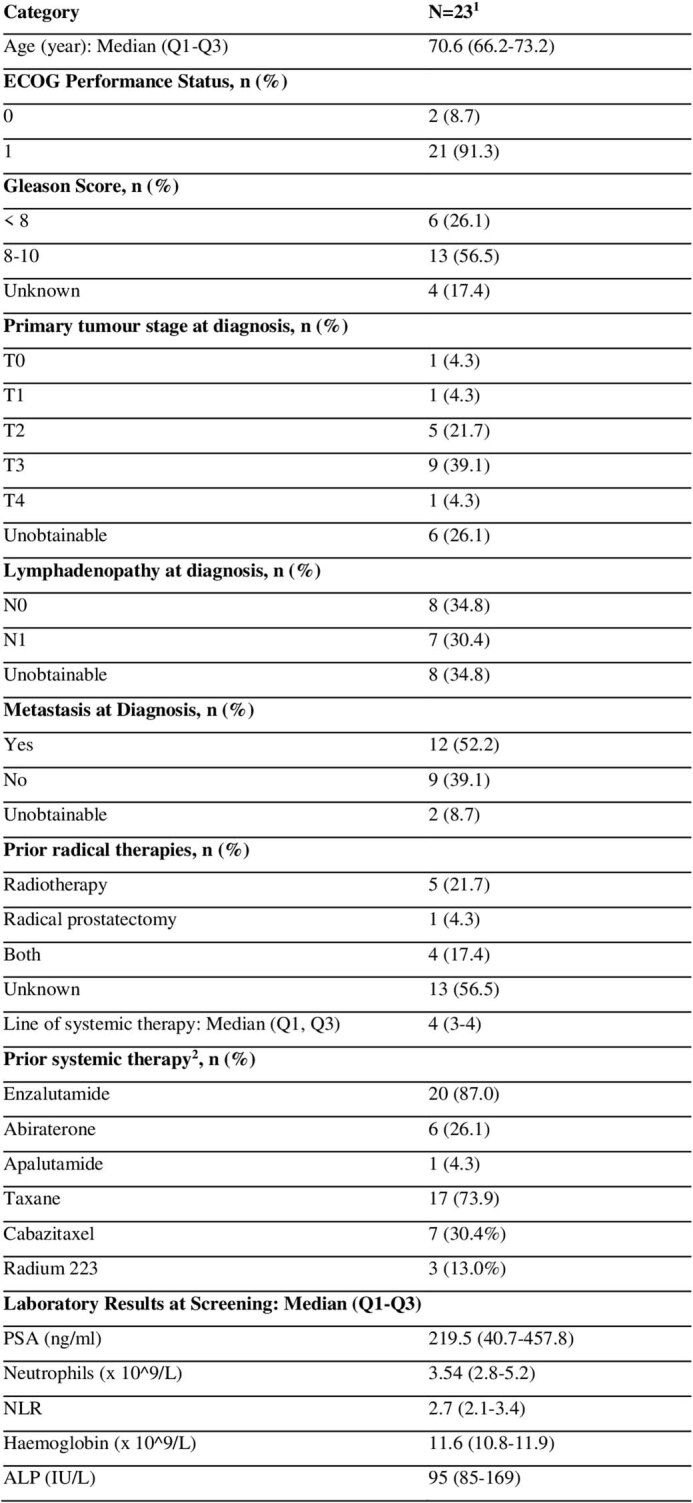
Baseline trial patient characteristics

^1^Two patients were replaced and not evaluable for DLT because they ceased treatment likely due to disease progression, and not for a DLT before completing the DLT period.

^2^All patients received and progressed on ADT.

ECOG = Eastern Cooperative Oncology Group.

**Extended Data Table 2:**
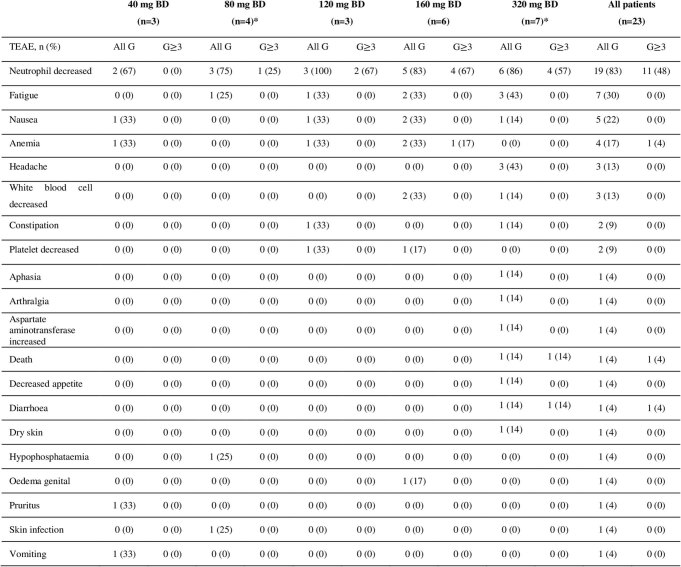
Treatment-emergent adverse events

Treatment-emergent adverse events (TEAEs) were defined as adverse events that are definitely, highly likely, or possibly related to the investigational agents occurring at any time whilst a patient was on study.

Data are n (%). Treatment-related adverse events of 23 patients were included the safety analysis.

G = grade according to NCI CTCAE v4.0.

*Two of 23 patients were not evaluable for DLT due to early cessation of the study drugs due to progressive disease before completing the DLT period; one patient died on study likely due to progressive disease after 6 days on study.

## Data Availability

The full study protocol is provided in the Supplementary Information. Bulk RNA-seq data from the SU2C–PCF cohort^34^ were downloaded from https://www.cbioportal.org/study/summary?id=prad_su2c_2019. Single-cell transcriptomic data from 15 mCRPC samples (https://www.nature.com/articles/s41591-021-01244-6) were downloaded from https://singlecell.broadinstitute.org/single_cell/study/SCP1244/transcriptional-mediators-of-treatment-resistance-in-lethal-prostate-cancer (study number SCP1244). Single-cell transcriptomic data from 11 patients with localized prostate cancer (https://www.nature.com/articles/s41467-021-27322-4) were downloaded from GEO accession GSE176031. De-identified, bulk RNA-seq data from the RMH cohort may be requested from the corresponding author (J.S.d.B.). Requests for the clinical and translational data from patient samples must provide clinically relevant rationale in adherence with the intent of the study and patients’ consent, and will be reviewed by the corresponding author (J.S.d.B.) and the ICR to determine whether the request is subject to any intellectual property, ethical and/or confidentiality considerations. A prompt response will be provided to such requests. Patient identifiers or information that may reveal the patient’s identity will not be shared. Any data or material that can be shared will be made available through a material transfer agreement with The ICR. Source data are provided with this paper.
