# Supplementary material for: Targeting myeloid chemotaxis to reverse prostate cancer therapy resistance
Source: Nature. 2023 Oct 16;623(7989):1053–61. doi: 10.1038/s41586-023-06696-z (PMC10686834; doi:10.1038/s41586-023-06696-z)
Supplement: Supplementary file 1 — Supplementary Tables 1–9, Figs. 1 and 2 and the study protocol. [file 41586_2023_6696_MOESM1_ESM.pdf]

nature portfolio

https://doi.org/10.1038/s41586-023-06696-z

Supplementary information

Targeting myeloid chemotaxis to reverse prostate cancer therapy resistance

In the format provided by the authors and unedited

Supplementary Information for:

Targeting myeloid chemotaxis to reverse prostate cancer therapy resistance

Supplementary Tables

Supplementary Table S1: Patient characteristics in cohort 1 and the validation cohort 2
Supplementary Table S2: Multivariable analyses of NLR/neutrophil associations with myeloid cell count by site 3
Supplementary Table S3: Myeloid signatures associations with NLR-related immune genes 4
Supplementary Table S4: Summary of tumour biopsy samples and analyses..5
Supplementary Table S5 Circulating tumour cell (CTC) counts for two CTC responders 6
Supplementary Table S6: Tumour molecular characteristics of evaluable patients 7
Supplementary Table S7: Antibodies for hyperplex immunofluorescence 8
Supplementary Table S8: Primary antibodies for six-colour immunofluorescence 9
Supplementary Table S9: Reagents for ELISA 10

Supplementary Figures

Supplementary Figure S1: Hyperplex immunofluorescence panel...11
Supplementary Figure S2: Boolean gating strategy..12

Study Protocol

Supplementary Table S1: Patient characteristics in cohort 1 and the validation cohort

	Cohort 1<br><br>(n=48)	Validation cohort<br><br>(n=57)
**Age**		
Median (IQR]	67.5 (63.4, 74.4)	69.7 (65.7, 72.9)
**Histology**		
Adenocarcinoma	48 (100%)	56 (98.2%)
Adenocarcinoma with focal neuroendocrine differentiation	0 (0%)	1 (1.8%)
**Gleason Score**		
6-7	13 (27.1%)	15 (26.3%)
8-10	20 (41.7%)	37 (64.9%)
Missing	15 (31.3%)	5 (8.8%)
**Site of biopsy**		
Bone	17 (35.4%)	15 (26.3%)
Liver	2 (4.2%)	4 (7.0%)
Lymph node	26 (54.2%)	32 (56.1%)
Soft tissue	3 (6.3%)	3 (5.3%)
TURP	0 (0%)	3 (5.3%)
**T stage at diagnosis**		
1	0 (0%)	0 (0%)
2	3 (6.3)	7 (12.3%)
3	17 (35.4%)	25 (43.9%)
4	5 (10.4%)	9 (15.8%)
Missing	23 (47.9%)	16 (28.1%)
**N stage at diagnosis**		
0	11 (22.9%)	11 (19.3%)
1	16 (33.3%)	16 (28.1%)
2	0 (0%)	4 (7.0%)
Missing	21 (43.8%)	26 (45.6%)
**M stage at diagnosis**		
0	14 (29.2%)	27 (47.4%)
1	21 (43.8%)	23 (40.4%)
Missing	13 (27.1%)	7 (12.3%)
**Prior lines of systemic therapy**		
Median (IQR]	3 (3, 4)	3 (3, 4)
**Prior taxane**	46 (95.8%)	52 (91.2%)
**Prior androgen receptor signalling inhibitor**	47 (97.9%)	53 (93.0%)

Supplementary Table S2: Multivariable analyses of NLR/neutrophil associations with myeloid cell count by site

COHORT 1

Coefficient	p-value (NLR)	p-value (neutrophils)
**Bone vs non-bone**		
NLR (left) Neutrophil (right)	2.88E-05	4.94E-05
Bone vs non-bone	0.132359	0.0151
**Bone vs all other sites**		
NLR (left) Neutrophil (right)	0.000187	0.000124
Liver vs bone	0.752798	0.544167
Lymph node vs bone	0.073718	0.006406
Soft tissue vs bone	0.794138	0.342202
**VALIDATION COHORT**		
**Bone v non-bone**		
NLR (left) Neutrophil (right)	0.02007	0.02764
Bone vs non-bone	0.00125	0.00102
**Bone vs all other sites**		
NLR (left) Neutrophil (right)	0.0164	0.036952
Liver vs bone	0.07567 .	0.085378 .
Lymph node vs bone	0.00115	0.000882
Soft tissue vs bone	0.66154	0.511946
TURP vs bone	0.02279	0.033197

p-values calculated using multivariable linear regression models.

Supplementary Table S3: Myeloid signatures associations with NLR-related immune genes

Signature	MDSC signature 1 (Calcinotto et al., Nature, 2018)			MDSC signature 2 (Alshetalwi et al., Sci Immunol, 2020)		
**Cohort**	**RMH CRPC TRANSCRIPTOME COHORT (n=95) (Fenor et al., European Urology Oncology, 2022)**					
**Gene**	**Correlation Coefficient**	**p-value**	***Statistically significant**	**Correlation Coefficient**	**p-value**	***Statistically significant**
**CXCL1**	0.51	1.32E-07	Yes	0.50	3.85E-07	Yes
**CXCL2**	0.47	1.36E-06	Yes	0.44	1.13E-05	Yes
**PLAUR**	0.68	4.31E-14	Yes	0.50	4.49E-07	Yes
**CEBPB**	0.45	6.52E-06	Yes	0.25	1.64E-02	No
**NFKB1**	0.22	3.01E-02	No	0.01	9.54E-01	No
**CXCL8**	0.12	2.50E-01	No	0.16	1.34E-01	No
**IL1RN**	0.37	2.77E-04	Yes	0.32	1.56E-03	Yes
**Cohort**	**SU2C/PCF CRPC TRANSCRIPTOME COHORT (n=159, PolyA+ capture subset) (Abida et al., PNAS, 2019)**					
**Gene**	**Correlation Coefficient**	**p-value**	***Statistically significant**	**Correlation Coefficient**	**p-value**	***Statistically significant**
**CXCL1**	0.40	2.40E-07	Yes	0.41	5.37E-08	Yes
**CXCL2**	0.40	1.35E-07	Yes	0.44	8.20E-09	Yes
**PLAUR**	0.60	3.81E-17	Yes	0.61	<2.2E-16	Yes
**CEBPB**	0.30	9.47E-05	Yes	0.40	1.35E-07	Yes
**NFKB1**	0.21	9.00E-03	No	-0.05	5.55E-01	No
**CXCL8**	0.26	1.06E-03	Yes	0.34	9.94E-06	Yes
**IL1RN**	0.47	4.40E-10	Yes	0.54	<2.2E-16	Yes

* Correlation coefficients and p-values from two-sided Spearman’s rank correlation analyses are shown. p-value threshold for significance was corrected for multiple hypothesis testing using the Bonferroni method, and p-values below 0.0036 were deemed statistically significant.

Supplementary Table S4: Summary of tumour biopsy samples and analyses

Patients (order as per Figure S4a)	Baseline biopsy available	Next generation sequencing	PTEN immunohistochemistry	AR-V7 immunohistochemistry	Hyperplex immunofluorescence
1	Yes	Baseline biopsy	Baseline biopsy	Baseline biopsy	Paired
2	Yes	Baseline biopsy	Baseline biopsy	Baseline biopsy	Paired
3	Yes	Baseline biopsy	Baseline biopsy	Baseline biopsy	Insufficient tumour
4	Yes	Baseline biopsy	Baseline biopsy	Baseline biopsy	Staining failed after 2 runs
5	Yes	Baseline biopsy	Baseline biopsy	Baseline biopsy	Paired
6	Yes	Baseline biopsy	Baseline biopsy	Baseline biopsy	Paired
7	Yes	Baseline biopsy	Baseline biopsy	Baseline biopsy	Paired
8	Yes	Baseline biopsy	Baseline biopsy	Baseline biopsy	Paired
9	Yes	Baseline biopsy	Baseline biopsy	Baseline biopsy	Insufficient tumour, high red cell content
10	Yes	Baseline biopsy	Baseline biopsy	Baseline biopsy	Paired
11	Yes	Baseline biopsy	Baseline biopsy	Baseline biopsy	Paired
12	Yes	Failed QC	Baseline biopsy	Baseline biopsy	Staining failed after 2 runs
13	Yes	Failed QC	Baseline biopsy	Baseline biopsy	Baseline only
14	Yes	Baseline biopsy	Baseline biopsy	Baseline biopsy	Paired
15	Yes	Baseline biopsy	Baseline biopsy	Baseline biopsy	Paired
16	No	cfDNA	Archival biopsy	Not available	Not available
17	Yes	Baseline biopsy	Baseline biopsy	Baseline biopsy	Paired
18	Yes	Baseline biopsy	Baseline biopsy	Baseline biopsy	Paired
19	Yes	Baseline biopsy	Baseline biopsy	Baseline biopsy	Paired
20	No	cfDNA	Archival biopsy	Not available	Not available
21	No	cfDNA	Not available	Not available	Not available

Supplementary Table S5: Circulating tumour cell (CTC) counts for two CTC responders

Trial timepoint	CTC/7.5 ml blood
**Patient 4 (patient number as per Fig. 4a)**	
C1D-14	9
C1D1	5
C1D15	2
C2D1	1
C3D1	5
C4D1	3
C5D1	5
C6D1	5
C7D1	3
C8D1	12
C9D1	14
C10D1	33
C11D1	34
C12D1	57
C13D1	52
C14D1	53
C15D1	96
C16D1	235
C17D1	86
C18D1	97
Treatment Discontinuation	119
**Patient 10 (patient number as per Fig. 4a)**	
C1D-14	11
C1D1	39
C1D15	26
C2D1	19
C3D1	0
C4D1	10
C5D1	46
C9D1	101
Treatment Discontinuation	129

Supplementary Table S6: Tumour molecular characteristics of evaluable patients

Patient (order as per Figure 4a)	AR-V7 Nuclear HS	AR-V7 protein status[a]	PTEN cytoplasmic HS	PTEN nuclear HS	PTEN protein status[b]	PTEN-PI3K pathway (NGS)	*AR* (NGS)	*TP53* (NGS)	*CDKN1B* (NGS)
1	35	Present	10	30	Present	Wildtype	Wildtype	Deletion	Wildtype
2	45	Present	5	40	Present	Pathogenic mutation of (*PTEN*)	Wildtype	Pathogenic mutation	Wildtype
3	30	Present	70	120	Present	Wildtype	Activating mutation	Wildtype	Wildtype
4	45	Present	50	50	Present	Wildtype	Amplificati on	Wildtype	Wildtype
5	70	Present	40	30	Present	Wildtype	Activating mutation	Wildtype	Wildtype
6	80	Present	5	0	Absent	Pathogenic mutation (*PTEN*)	Wildtype	Deletion	Wildtype
7	40	Present	80	30	Present	Wildtype	Wildtype	Wildtype	Wildtype
8	50	Present	190	150	Present	Wildtype	Wildtype	Wildtype	Wildtype
9	0	Absent	0	0	Absent	Wildtype	Wildtype	Wildtype	Wildtype
10	0	Absent	100	0	Present	Wildtype	Wildtype	Wildtype	Wildtype
11	130	Present	100	140	Present	Wildtype	Amplificati on	Wildtype	Wildtype
12	15	Present	100	20	Present	NA	NA	NA	NA
13	10	Present	0	0	Absent	NA	NA	NA	NA
14	200	Present	160	105	Present	Activating mutation (*PIK3CA*)	Amplificati on	Pathogenic mutation	Wildtype
15	215	Present	50	30	Present	Wildtype	Amplificati on	Wildtype	Wildtype
16	NA	NA	0[d]	0[d]	Absent	Wildtype[c]	Wildtype[c]	Wildtype[c]	Wildtype[c]
17	7	Absent	100	70	Present	Wildtype	Wildtype	Wildtype	Deletion
18	6	Absent	130	135	Present	Wildtype	Wildtype	Wildtype	Deletion
19	0	Absent	0	0	Absent	Deletion (*PTEN*)	Wildtype	Wildtype	Wildtype
20	NA	NA	0[d]	0[d]	Absent	Wildtype[c]	Wildtype[c]	Pathogenic[c] mutation	Wildtype[c]
21	NA	NA	NA	NA	NA	Activating mutation (*AKT1*)[c]	Wildtype[c]	Pathogenic[c] mutation	Wildtype[c]

[a]AR-V7 protein status: present defined as nuclear HS $\geq$ 10
[b]PTEN protein status: absent defined as HS < 10 in both the cytoplasm and nucleus
[c]cfDNA sample used for NGS when tumour biopsy was not available
[d]Archival sample used as baseline CRPC biopsy was not obtained.
HS = Histo-score
NA = Not available
cfDNA = cell-free DNA

Supplementary Table S7: Antibodies for hyperplex immunofluorescence

Antibody	Vendor	Host species	Clone	Lot. No.	Working dilutions	Catalogue number	Primary antibody incubation time (minutes)
**Primary antibodies**							
**NCAM1 (CD56)**	Abcam	Mouse	123C3.D5	1020624-1	1:25	ab270248	8
**CXCR2**	Abcam	Rabbit	EPR22301-103	GR3378654-6	1:100	ab245982	8
**FOXP3**	eBioscience™ (Thermo Fisher)	Mouse	236A/E7	2378013	1:25	14-4777-82	8
**CD15**	Dako	Mouse	Carb-3	11397463	1:25	M3631	8
**Granzyme B**	CST	Rabbit	D6E9W	6	1:50	46890	8
**CD14**	Abcam	Rabbit	EPR3652	GR211954-7	1:100	ab133503	8
**CD138**	Dako	Mouse	MI15	41415171	1:25	M7228	8
**CD11b**	Abcam	Rabbit	EP1345Y	GR3219233-5	1:200	ab52478	8
**MUM1**	Dako	Mouse	MUM1p	41455977	1:25	M7259	8
**CD8**	Dako	Mouse	C8/144B	41389238	1:50	M7103	8
**CD163**	Abcam	Rabbit	EPR19518	GR3339055-17	1:100	ab182422	8
**CD68**	Dako	Mouse	PG-M1	41337737	1:50	M0876	8
**Chromogranin A**	Dako	Mouse	DAK-A3	41449632	1:50	M0869	4
**HLA-DR**	Abcam	Mouse	TAL 1B5	GR3378141-4	1:400	ab20181	8
**CD4**	Abcam	Rabbit	EPR6855	GR3276764-30	1:100	ab133616	4
**Pan-cytokeratin**	Dako	Mouse	AE1/AE3	11445606	1:50	M3515	4
**CD20**	Dako	Mouse	L26	41367309	1:400	M0755	4
**CD38**	Abcam	Rabbit	EPR4106	GR3402044-1	1:7500	ab226034	4
**Synaptophysin**	Leica Biosystems	Mouse	27G12	6081141	1:50	SYNAP-299-L-CE	4
**CD206/MRC1**	CST	Rabbit	E2L9N	1	1:200	91992	4
**Secondary antibodies**							
**Goat anti-Mouse IgG (H+L) Highly Cross-Adsorbed Secondary Antibody, Alexa Fluor™ Plus 555**	Invitrogen (Thermo Fisher Scientific)	Goat	Polyclonal	WL333735	1:200	A32727	2
**Goat anti-Rabbit IgG (H+L) Highly Cross-Adsorbed Secondary Antibody, Alexa Fluor™ Plus 647**	Invitrogen (Thermo Fisher Scientific)	Goat	Polyclonal	WL333739	1:400	A32733	2

Other incubation times:

- Secondary antibody incubation time (all staining cycles): 2 minutes
- Elution time (all staining cycles, except staining cycle 1): 2 minutes

Supplementary Table S8: Primary antibodies for six-colour immunofluorescence

Antibody	Vendor	Host species	Clone	Lot. No.	Working dilutions	Catalogue number
**CXCR2**	Abcam	Rabbit	EPR22301-103	GR3378654-5	1:500	ab245982
**CD11b**	Abcam	Rabbit	EP1345Y	GR3219233-10	1:2500	ab52478
**CD15**	Dako	Mouse	Carb-3	11397463	1:500	M3631
**CD14**	Abcam	Rabbit	EPR3652	GR211954-8	1:1500	ab133503
**HLA-DR**	Abcam	Mouse	TAL.1B5	GR3456090-1	1:500	ab20181

Supplementary Table S9: Reagents for ELISA

CXCL7

All reagents, unless stated, are supplied by R&D systems in the kit LXSAHM-01 for CXCL7; details are recorded in the table below:

Reagent	Storage	Part number
Human standard cocktail L	2-8ºC	894863
Human magnetic premixed microparticle cocktail	2-8ºC	894723
Human Premixed Biotin-Ab cocktail	2-8ºC	893988
Streptavidin-PE concentrate	2-8ºC	893535
Diluent RD2-1	2-8ºC	895970
Calibrator Diluent RD6-52	2-8ºC	895438
Wash buffer concentrate	2-8ºC	895003
Microplate	2-8ºC	641385
Plate sealers	RT	640445
Human serum (Sigma-Aldrich)	-80ºC	H4522-100ML

CXCL2, CXCL5, CXCL6, CXCL8

All reagents, unless stated, are supplied by R&D systems in the kit LXSAHM-01 for CXCL2, CXCL5, CXCL6, CXCL8; details are recorded in the table below:

Reagent	Storage	Part number
Human standard cocktail B	2-8ºC	893901
Human standard cocktail K	2-8ºC	894824
Human magnetic premixed microparticle cocktail	2-8ºC	894723
Human Premixed Biotin-Ab cocktail	2-8ºC	893988
Streptavidin-PE concentrate	2-8ºC	893535
Diluent RD2-1	2-8ºC	895970
Calibrator Diluent RD6-52	2-8ºC	895438
Wash buffer concentrate	2-8ºC	895003
Microplate	2-8ºC	641385
Plate sealers	RT	640445
Human serum (Sigma-Aldrich)	-80ºC	H4522-100ML

CXCL1

All reagents, unless stated, are supplied by R&D systems in the kit DRG00B; details are recorded in the table below:

Reagent	Storage	Part number
Human GROα microplate	2-8ºC	890150
Human GROα standard	2-8ºC	890152
Human GROα conjugate	2-8ºC	890151
Assay Diluent RD1U	2-8ºC	895138
Calibrator Diluent RD6-69	2-8ºC	896012
Wash buffer concentrate	2-8ºC	895003
Color reagent A	2-8ºC	895000
Color reagent B	2-8ºC	895001
Stop solution	2-8ºC	895032
Plate sealers	RT	N/A
Human serum (Sigma-Aldrich)	-80ºC	H4522-100ML

Supplementary Figure S1: Hyperplex immunofluorescence panel

a

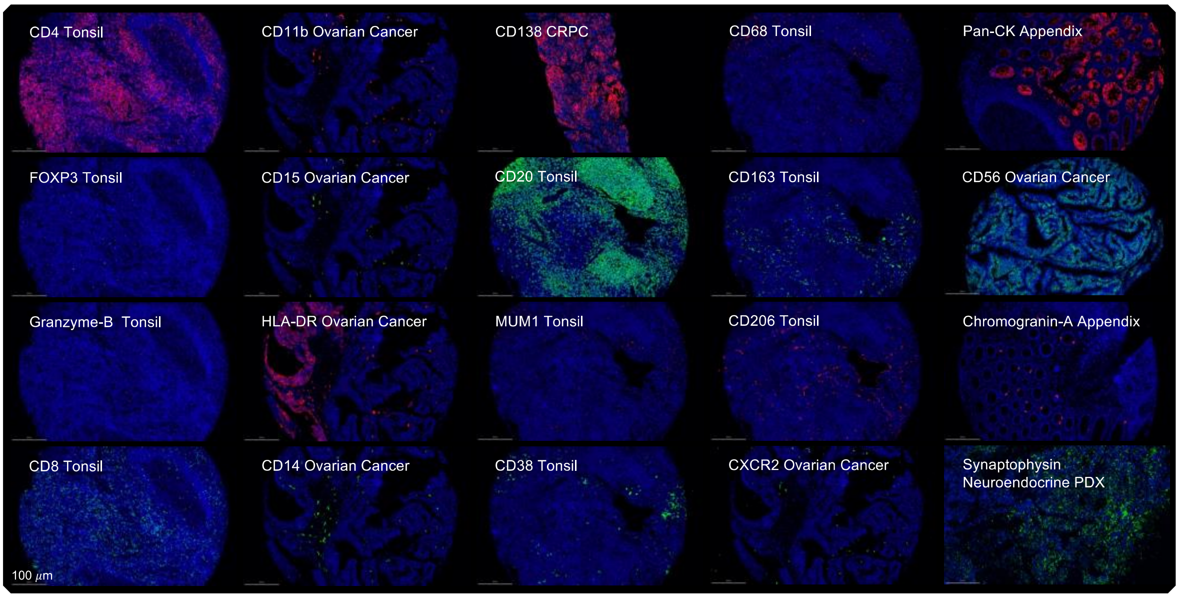

b

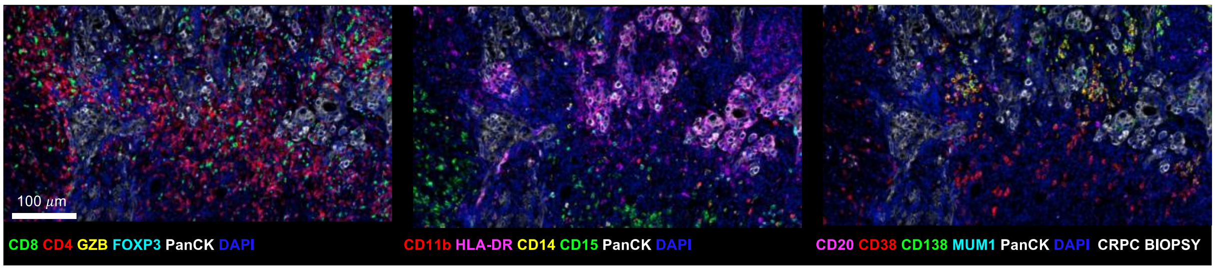

a, Validation of a hyperplex immunofluorescence panel using the COMET™ platform. Each panel shows example staining of relevant positive controls for the marker (ovarian cancer, tonsil, appendix, CRPC, and CRPC xenograft with known neuroendocrine phenotype and marker staining). **b**, Example immunofluorescence image of a CRPC biopsy (same field) showing combinations of markers for T cells, myeloid cells, and B cells. All scale bars = 100 μm.

Supplementary Figure S2: Boolean gating strategy

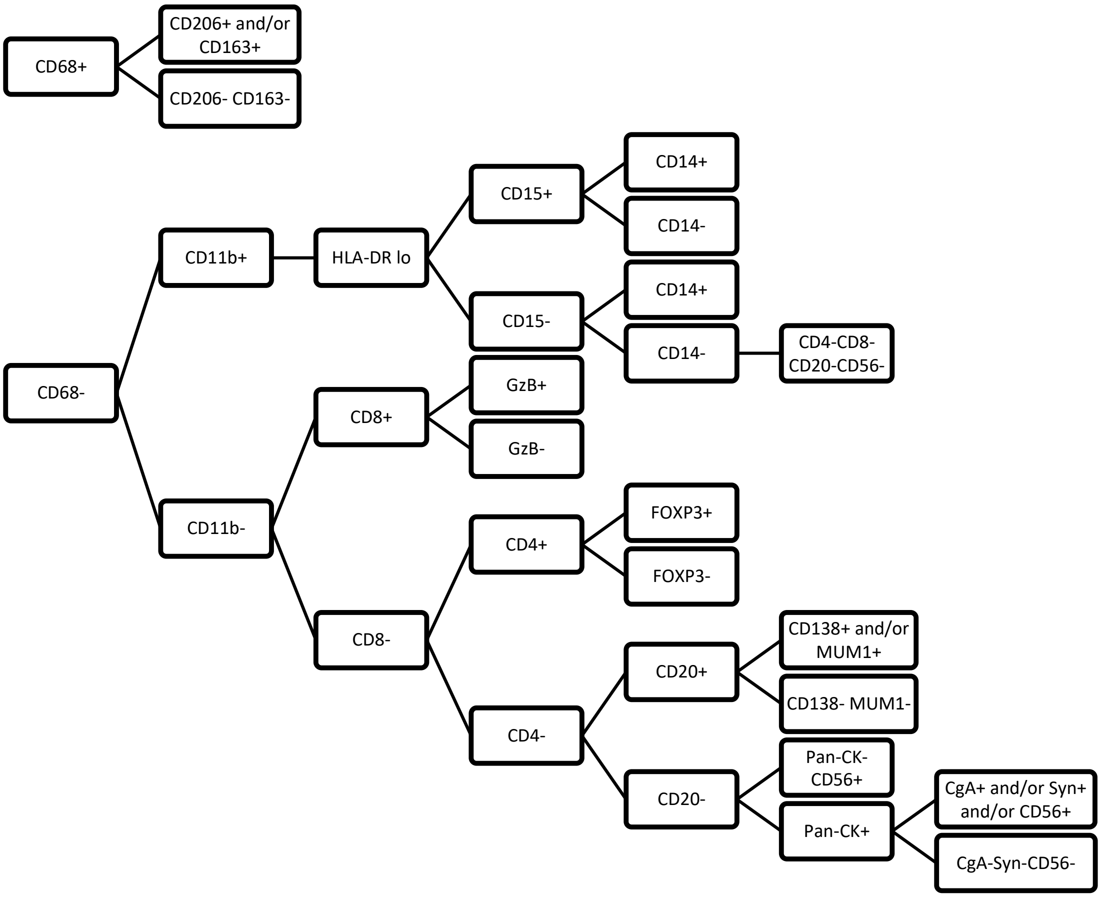

Boolean gating strategy for phenotyping of cells in CRPC biopsies stained by hyperplex IF. GzB = granzyme-B, CgA = chromogranin-A, Syn = synaptophysin.

ACE: Proof of concept Phase I/II trial of the CXCR2 antagonist **A**ZD5069, administered in **c**ombination with **e**nzalutamide, in patients with metastatic castration resistant prostate cancer (mCRPC)

Sponsor protocol number:	CCR4500
EudraCT number:	2016-003141-28
IRAS number	211557
**Chief Investigator:**	Professor Johann de Bono<br>Professor of Experimental Cancer Medicine and Head of Drug Development Unit<br>The Institute of Cancer Research and Royal Marsden NHS Foundation Trust<br>Drug Development Unit

**Co-Investigators:**	Dr Adam Sharp<br>Medical Research Council Clinical Research Training Fellow and Specialist Registrar in Medical Oncology<br><br>Dr Christina Guo

Clinical Fellow
Drug Development Unit/Prostate Cancer Targeted Therapies Group

Professor Christina Yap
The Institute of Cancer Research
15 Cotswold Road, Sutton
Surrey SM2 5PT

PARTICIPATING INVESTIGATORS AND CENTRES:

**Participating centre:**	The Royal Marsden NHS Foundation Trust<br>Drug Development Unit/Prostate Cancer Targeted Therapies Group<br>Downs Road<br>Sutton<br>Surrey SM2 5PT
**Principal Investigator:**	Professor Johann de Bono<br>Professor of Experimental Cancer Medicine and Head of Drug Development Unit
**Participating centre:**	Oncology Institute of Southern Switzerland (IOSI)<br>Ospedale San Giovanni<br>CH6500 Bellinzona<br>Switzerland
**Principal Investigator:**	Dr Ursula Dr. Vogl.<br>Oncology Institute of Southern Switzerland (IOSI)
**Participating centre:**	Belfast City Hospital Cancer Centre<br>51 Lisburn Road
**Principal Investigator:**	Dr Suneil Jain<br>Centre for Cancer Research and Cell Biology

**Participating centre:**	University Hospital<br>Southampton NHS Foundation Trust<br>Tremona Road<br>Southampton<br>Hampshire<br>SO16 6YD
**Principal Investigator:**	Dr Simon Crabb<br>Cancer Sciences Unit

PROTOCOL CONTRIBUTORS AND STATISTICIAN:

Protocol contributors	Alison Turner<br>Head of Investigator Initiated Research<br>The Institute of Cancer Research<br>Drug Development Unit<br>15 Cotswold Road<br>Belmont, Sutton<br>Surrey, SM2 5PT
	Ruth Matthews<br>Clinical Trials Manager<br>The Institute of Cancer Research<br>Drug Development Unit<br>15 Cotswold Road<br>Belmont, Sutton<br>Surrey, SM2 5PT
Methodological Lead	Professor Christina Yap<br>Professor of Clinical Trials Biostatistics<br>The Institute of Cancer Research<br>Clinical Trials and Statistics Unit<br>15 Cotswold Road<br>Belmont, Sutton<br>Surrey, SM2 5PT
Statistician	Jan Rekowski<br>Senior Statistician<br>The Institute of Cancer Research<br>Clinical Trials and Statistics Unit<br>15 Cotswold Road<br>Belmont, Sutton<br>Surrey, SM2 5PT

TABLE OF CONTENTS

TITLE PAGE
TABLE OF CONTENTS
LIST OF ABBREVIATIONS AND DEFINITION OF TERMS
PROTOCOL SIGNATURES

1 PROTOCOL SUMMARY 12

1.1 Full title . 12
1.1.1 Short title 12
1.2 Clinical trial objectives and endpoints 12
1.2.1 Primary objectives and endpoints 12
1.2.2 Secondary objectives and endpoints 13
1.2.3 Exploratory objectives 15
1.3 Design . 16
1.4 Administration schedule 16
1.4.1 Phase I safety run in cohort 16
1.4.2 Phase II cohorts 17
1.5 Treatment group 17
1.5.1 Phase I safety run in cohort 17
1.5.2 Phase II cohorts 17
1.6 Trial timelines and accrual rate 18

2 INTRODUCTION 18

2.1 Background 18
2.1.1 Prostate cancer 18
2.1.2 Neutrophil to Lymphocyte ratio 18
2.1.3 Myeloid derived suppressor cells 19
2.2 Overview of enzalutamide 19
2.2.1 Pre-clinical experience with enzalutamide 20
2.2.2 Pharmacokinetics and drug metabolism of enzalutamide in animals 20
2.2.3 Toxicology and safety pharmacology summary of enzalutamide 20
2.2.4 Clinical experience of enzalutamide 21
2.2.5 Pharmacokinetics and drug metabolism of enzalutamide in humans 22
2.3 Overview of AZD5069 23

2.3.1 Pre-clinical experience with AZD5069 23
2.3.2 Pharmacokinetics and drug metabolism of AZD5069 in animals 23
2.3.3 Toxicology and safety pharmacology summary of AZD5069 24
2.3.4 Clinical experience of AZD5069 25
2.3.5 Pharmacokinetics and drug metabolism of AZD5069 in humans 27
2.4 Rationale for the proposed trial 27
3 TRIAL DESIGN 27
3.1 Clinical trial objectives and endpoints 28
3.1.1 Primary objectives and endpoints 28
3.1.2 Secondary objectives and endpoints 28
3.1.3 Exploratory objectives 31
3.2 Definition of dose limiting toxicity 32
3.3 Definition of maximum tolerated dose 33
3.4 Safety Review Committee and Independent Data Monitoring Commitee 33
3.4.1 Phase I safety run in cohort 33
3.4.2 Phase II reversal of enzalutamide resistance cohort 34
3.5 Patient evaluability 34
3.6 Definition of disease progression (all stages) 35
3.7 Design of the clinical trial 36
4 PATIENT SELECTION 41
4.1 Eligibility criteria 41
4.1.1 Inclusion criteria: 41
4.1.2 Exclusion criteria: 42
4.2 Patient enrolment 44
5 TREATMENT 44
5.1 Dosing schedule/treatment schedule 45
5.1.1 Phase I safety run cohort 45
5.1.2 Phase II cohorts 46
5.2 Dose escalation scheme 46
5.2.1 Phase I safety run cohort 46
5.3 Dose Delays/Dose modifications and Adverse Event Managment 47
5.3.1 Enzalutamide dose reduction/dose adjustment 47
5.3.2 AZD5069 dose reduction/dose adjustment 48

5.3.3 Intra-patient dose escalations 48
5.3.4 Dose interruptions due to COVID-19 48
5.4 Duration of treatment 48
5.5 Concomitant medication and treatment 49
5.5.1 Enzalutamide 49
5.5.2 AZD5069 50
5.5.3 Corticosteroids 51
6 PHARMACEUTICAL INFORMATION 51
6.1 Supply of enzalutamide and AZD5069 52
6.2 Pharmaceutical data 52
6.2.1 Formulation of enzalutamide and AZD5069 52
6.2.2 Storage conditions 52
6.3 Administration of AZD5069 and enzalutamide 52
6.4 Enzalutamide and AZD5069 accountability 52
7 INVESTIGATIONS SCHEDULE 53
7.1 Pre-treatment evaluations 53
7.1.1 Obtaining written informed consent 53
7.1.2 Evaluations within four weeks (28 days) 54
7.1.3 Evaluations within one week (7 days) 55
7.2 Evaluations during the trial 56
7.3 Evaluations at ‘off-study’ visit 58
7.4 Follow-up . 59
7.5 Schedule of events 60
7.5.1 Phase I safety run in cohort 60
7.5.2 Phase II reversal of enzalutamide resistance cohort 62
7.5.3 Phase II enzalutamide resistance run in cohort 64
8 Pharmacokinetic and Pharmacodynamic ASSESSMENTS 66
8.1 Pharmacokinetic assays 66
8.2 Pharmacodynamic biomarker assays 66
8.2.1 Archival tumour tissue 66
8.2.2 Tumour sampling 66
8.2.3 Blood sampling 67
8.3 Schedule of research samples 69

8.3.1 Phase I safety run in cohort 69
9 ASSESSMENT OF SAFETY 70
9.1 Adverse event definitions 70
9.1.1 Adverse event 70
9.1.2 Serious adverse events (SAEs) 71
9.1.3 Determining adverse event causality 71
9.1.4 Expectedness 72
9.1.5 Suspected, unexpected, serious, adverse reactions (SUSARs) 72
9.2 Expedited reporting of serious adverse events to the Pharmacovigilance Officer, DDU 72
9.2.1 Events exempt from being reported as SAEs 73
9.3 Recording of adverse events and serious adverse events in eCRFs 73
9.3.1 Recording of anaemia and hypokalaemia 73
9.4 Follow-up of adverse events 74
9.5 Urgent safety measures 74
9.6 Pregnancy . 74
10 ASSESSMENT OF EFFICACY 75
10.1 Measurement of disease 75
10.2 Timing and type of tumour assessments 75
10.3 Baseline evaluations 76
10.4 Evaluations during and at ‘off-study’ 76
10.5 Tumour response 76
10.6 Recording of response in the eCRF 77
11 Discontinuation of treatment/ withdrawal from trial 77
12 DEFINING THE End of triaL 77
13 DATA ANALYSIS and statistical considerations 78
13.1 Statistical design 78
13.2 Analysis Populations 79
13.3 Presentation of data 79
13.4 Safety . 80
13.5 Pharmacokinetics 80
13.6 Pharmacodynamics 80
13.7 Anti-tumour activity 80

13.7.1 Phase I safety run in cohort 80
13.7.2 Phase II reversal of enzalutamide resistance cohort 81
14 ADMINISTRATION 81
14.1 Protocol deviations and amendments 81
14.2 Completion of the case report form (CRF) 82
14.3 Trial performance and monitoring 82
14.3.1 Management of the study 82
14.3.2 Source document verification 83
14.4 Clinical study report 83
14.5 Record retention 83
14.6 Ethical considerations 84
14.7 Indemnity . 84
14.8 Publication policy and press releases 84
15 REFERENCES 84
16 APPENDICES 86
16.1 APPENDIX 1: WHO PERFORMANCE SCALE 86
16.2 APPENDIX 2: DECLARATION OF HELSINKI 88
16.3 APPENDIX 3: MEASUREMENT OF DISEASE 92
16.4 APPENDIX 4: NEW YORK HEART ASSOCIATION (NYHA) SCALE 97
16.5 APPENDIX 5: POTENT/MODERATE CYP 3A4 INHIBITORS FOR PATIENTS ON AZD5069 . 97

LIST OF ABBREVIATIONS AND DEFINITION OF TERMS

	Abbreviation	Definition
**A**	ABPI	Association of the British Pharmaceutical Industry
	AE	adverse event
	alk phos	alkaline phosphatase
	ALT	alanine aminotransferase
	ANC	absolute neutrophil count
	AST	aspartate aminotransferase
	AUC	area under the curve
	BP	blood pressure
	BSA	body surface area
**C**	^{0}C	degrees Celsius
	cfDNA	Cell-free DNA
	CI	Chief Investigator
	CLT	total body clearance

LIST OF ABBREVIATIONS AND DEFINITION OF TERMS

	Abbreviation	Definition
	C_{max}	maximum observed plasma concentration
	CR	complete response
	CRA	Clinical Research Associate
	CRF	case report form
	CRP	C-reactive protein
	CT	computerised tomography
	CTA	clinical trial authorisation
	CTCAE	Common Terminology Criteria for Adverse Events
**D**	Day	calendar day
	DDU	Drug Development Unit
	DLT	dose limiting toxicity
**E**	ECG	electrocardiogram
	EDTA	ethylene diamine tetra-acetic acid
	EPD	early progressive disease
**F**	FDG	fluorodeoxyglucose
	FU	Formulation Unit
	FSH	follicle stimulating hormone
	GCP	Good Clinical Practice
	g/dL	gram(s) per decilitre
	GMP	Good Manufacturing Practice
**H**	Hb	Haemoglobin
	HbA1c	Glycosolated haemoglobin (HbA1c),
	HR	Hazard Ratio
	HRA	Health Research Authority
	HCG	human chorionic gonadotropin
	IB	Investigator Brochure
**I**	ICH GCP	International Conference on Harmonisation of Good Clinical Practice
	IHC	Immunohistochemistry
	IMP	investigational medicinal product
	ITF	Investigator Trial File
**L**	LVEF	left ventricular ejection fraction
**M**	MAD	maximum administered dose
	mg/m^2	milligram per square metre
	MHRA	Medicines and Healthcare products Regulations Agency
	MRI	magnetic resonance imaging
	MRT	mean residence time
	MTD	maximum tolerated dose
**N**	NCI	National Cancer Institute
**P**	PBMC	Peripheral blood mononuclear cells
	PD	progressive disease
	PI	Principal Investigator
	PK	pharmacokinetic
	PR	partial response
	PSA	prostate specific antigen
**Q**	QC	quality control
	QP	Qualified Person

LIST OF ABBREVIATIONS AND DEFINITION OF TERMS

	Abbreviation	Definition
**R**	REC	Research Ethics Committee
	RECIST	Response Evaluation Criteria in Solid Tumours
**S**	SAE	serious adverse event
	SD	stable disease
	SDV	source data verification
	SOP	standard operating procedure
	SUSAR	suspected unexpected serious adverse (drug) reaction
**T**	$T_{1/2}$	terminal elimination half-life
	T_{max}	time to reach C_{max}
	TSH	thyroid stimulating hormone
**U**	ULN	upper limit of normal
	USM	urgent safety measure
**V**	V_{SS}	steady state volume of distribution
	WBC	white blood cell
	WHO	World Health Organisation

PROTOCOL SIGNATURES

Investigator Signature:
I have read and agree to the protocol, as detailed in this document. I am aware of my responsibilities as an Investigator under the UK Clinical Trials Regulations[1], the guidelines of Good Clinical Practice (GCP)[2], the Declaration of Helsinki (appendix 2), the applicable regulations of the relevant NHS Trusts and the trial protocol. I agree to conduct the trial according to these regulations and guidelines and to appropriately direct and assist the staff under my control, who will be involved in the trial, and ensure that all staff members are aware of their clinical trial responsibilities.

INVESTIGATOR'S NAME: ______________________________

SIGNATURE: ______________________________
DATE: ______________________________

1 PROTOCOL SUMMARY

2 Full title

Proof of concept phase I/II trial of the CXCR2 antagonist AZD5069, administered in combination with enzalutamide, in patients with metastatic castration resistant prostate cancer (mCRPC) (ACE)

3 Short title

Phase I/II trial of AZD5069 in combination with enzalutamide in mCRPC

4 Clinical trial objectives and endpoints

5 Primary objectives and endpoints

Phase I safety run in cohort

Primary objective	Endpoint
To identify the safety and tolerability of enzalutamide and AZD5069 when given in combination continuously.	To identify the dose-limiting toxicities (DLTs), estimate the maximum tolerated dose (MTD) and identify the recommended phase II dose (RP2D) of AZD5069 administered in combination with enzalutamide at 160mg OD.

Phase II reversal of enzalutamide resistance cohort

1 The Medicines for Human Use (Clinical Trials) Regulations (S.I. 2004/1031) and any subsequent amendments to it.
2 ICH Harmonised Tripartite Guideline E6: Note for Guidance on Good Clinical Practice (CPMP/ICH/135/95) Step 5, adopted by CPMP July 1996.

Primary objective	Endpoint
To estimate the antitumour activity of AZD5069 in combination with enzalutamide as measured by response rate.	Antitumour activity will be defined by response rate on the basis of the following outcomes; if any of these occur, patients will be considered to have responded:<br><br>• Prostate specific antigen (PSA) decline ≥ 50% criteria confirmed 4 weeks or later and/or,<br>• Confirmed soft tissue objective response by RECIST (v1.1) in patients with measurable disease and/or,<br>• ONLY for patients with detectable circulating tumour cell count (CTC) of ≥ 5/7.5ml blood at baseline, conversion of CTC <5/7.5ml blood nadir.<br><br>For disease progression (see section 3.6) the Prostate Cancer Working Group 2 (PCWG2) criteria and RECIST (v1.1) criteria will be used. Treatment failure will be defined as:<br>• Progression of soft tissue/visceral disease by RECIST (v1.1) and/or,<br>• Progression of bone disease by PCWG2 bone scan criteria and/or<br>• Progression of PSA by PCWG2 PSA criteria.

6 Secondary objectives and endpoints

Phase I safety run in cohort

Secondary objectives	Endpoint
To characterise the pharmacokinetics (PK) of enzalutamide and AZD5069 when administered in combination and assess drug interaction.	Determination of the plasma levels of enzalutamide and AZD5069 using validated assays.
To characterise the pharmacodynamics (PD) of AZD5069 and enzalutamide when administered in combination.	Identify those patients with a neutrophil to lymphocyte ratio (NLR) ≥ 3 (at baseline) that convert to an NLR < 3

Secondary objectives	Endpoint
	(blood nadir) with AZD5069 and enzalutamide in combination.<br>Identify those patients whose circulating myeloid derived suppressor cells (MDSCs) and intratumoral MDSCs reduce by 50% with AZD5069 and enzalutamide in combination.
To estimate the antitumour activity of AZD5069 in combination with enzalutamide as measured by response rate.	Antitumour activity will be defined by response rate on the basis of the following outcomes; if any of these occur, patients will be considered to have responded:<br>• PSA decline ≥ 50% criteria confirmed 4 weeks or later and/or,<br>• Confirmed soft tissue objective response by RECIST (v1.1) in patients with measurable disease and/or,<br>• ONLY for patients with detectable circulating tumour cell count (CTC) of ≥ 5/7.5ml blood at baseline, conversion of CTC <5/7.5ml blood nadir.<br><br>For disease progression (see section 3.6) the PCWG2 criteria and RECIST (v1.1) criteria will be used. Treatment failure will be defined as:<br>• Progression of soft tissue/visceral disease by RECIST (v1.1) and/or,<br>• Progression of bone disease by PCWG2 bone scan criteria and/or<br>• Progression of PSA by PCWG2 PSA criteria.

Phase II reversal of enzalutamide resistance cohort

Secondary objectives	Endpoint
To establish the maximum PSA decline at any point on trial and at 12 weeks for patients on AZD5069 and enzalutamide.	Maximal PSA decline at any time during the trial and PSA decline after 12 weeks (as per PCWG2 criteria) of combination treatment.
To estimate overall survival (OS) in these patients.	Overall survival will be measured from the date of AZD5069 addition to enzalutamide to the date of death (whatever cause). Survival time of living

Secondary objectives	Endpoint
	patients will be censored on the last date of patient is known to be alive or lost to follow up.
To estimate the radiologic progression free survival (rPFS) on the combination of AZD5069 and enzalutamide in these patients.	rPFS will be measured from the date of AZD5069 addition to enzalutamide until:<br>• Progression of soft tissue/visceral disease by RESIST and/or,<br>• Progression of bone disease by PCWG2 bone scan criteria and/or,<br>• Death of any cause<br><br>Patients withdrawn for any reason prior to radiological progression then the patient should be assessed until radiological progression has occurred. If however they have started another treatment then they will be censored at the start of the new treatment.
To assess the effects of AZD5069 and enzalutamide on the number of circulating tumour cells.	CTC fall by >30% will be expressed as the proportion of patients that have demonstrated a CTC fall of >30% after 12 weeks of combination treatment.
To further evaluate the safety and tolerability of the combination in patients who progress on enzalutamide.	Recording the population exposure to the AZD5069 and enzalutamide combination will summarise safety. Adverse events will be graded according to the Common Terminology Criteria for Adverse Events (CTCAE) v4.0.
To further characterise the PD profile of AZD5069 and enzalutamide when administered in combination.	Identify those patients with a neutrophil to lymphocyte ratio (NLR) ≥ 3 (at baseline) that convert to an NLR < 3 (blood nadir) with AZD5069 and enzalutamide in combination.<br><br>Identify those patients whose circulating myeloid derived suppressor cells (MDSCs) and intratumoral MDSCs reduce by 50% with AZD5069 and enzalutamide in combination.

7 Exploratory objectives

Phase I safety run in cohort

Exploratory objectives
Evaluating the effect of AZD5069 with and without enzalutamide on chemokine expression (such as IL-6, IL-8, CXCL-1, CXCL-2, CXCL-5), MDSC infiltration and tumour cell Ki67 in serial tumour biopsies using immunohistochemistry (IHC) and multi-colour immunofluorescence (MC-IF).
To evaluate the impact of CXCR2 inhibition in PTEN loss and PTEN wildtype cancers (immunohistochemical H score <30).
Evaluating the effect of AZD5069 treatment on circulating cytokine levels (such as GM-CSF, IL-6, IL-8, CXCL-1, CXCL-2 and CXCL-5) in whole blood using an enzyme-linked immunosorbent assay (ELISA).
Evaluating the effect of treatment on whole blood transcriptome profiles.
Evaluating the effect of treatment on white blood cells by whole blood immunophenotyping using fluorescence-activated cell sorting (FACS).
To correlate cell-free DNA (cfDNA) with response to treatment and disease progression.

Phase II reversal of enzalutamide resistance cohort

Exploratory objectives
To investigate the effect of AZD5069 and enzalutamide on chemokine expression such as CXCL-1, CXCL-2, CXCL-5, IL-6, IL-8 and tumour cell Ki67 expression in serial tumour biopsies using IHC and IF.
To study the effects of AZD5069 and enzalutamide on tumour biopsy infiltration by MDSC utilizing IHC and MC-IF.
To evaluate the impact of CXCR2 inhibition in PTEN loss and PTEN wildtype cancers (immunohistochemical H score <30).
To report the effect of AZD5069 and enzalutamide on circulating chemokine and cytokine levels (such as GM-CSF, IL-6, IL-8, CXCL-1, CXCL-2 and CXCL-5) in whole blood by ELISA.
To evaluate the effect of the combination on the NLR in whole blood.
To evaluate the combination’s impact on whole blood transcriptomes.
To report the effect of the combination on circulating white blood cells by immunophenotyping through FACS.
To correlate cfDNA with response to treatment and disease progression.

8 Design

This is a multi-centre, proof of concept, Phase I/II trial.

9 Administration schedule

This is a phase I/II trial of the combination of AZD5069 and enzalutamide in patients with mCRPC.

10 Phase I safety run in cohort

During the phase I study, patients will receive AZD5069 orally twice daily (BD) in combination with enzalutamide at 160mg orally once daily continuously. The starting

dose of AZD5069 will be 40mg BD (dose level 1): other doses to be evaluated will include 80mg BD (dose level 2), 120mg BD (dose level 3), 160mg BD (dose level 4) and 320mg BD (dose level 5) in order to determine the MTD and RP2D to take forward to a Phase II reversal of resistance cohort. Intermediate dose levels such as 240mg BD may also be evaluated. During dose levels 1 to 4, patients will start with AZD5069 monotherapy for two weeks before commencing the combination. At all other dose levels, the two agents will be started concurrently. In addition, if agreed by the SRC, intermediate dose levels such as 240 mg taken BD will be explored.

11 Phase II cohorts

If the MTD of AZD5069 is greater than 160 mg BD, two different dose levels may be taken forward to the phase II study to determine efficacy. **Only patients who have experienced disease progression after at least 12 weeks of treatment with enzalutamide, apalutamide or darolutamide will be eligible for this trial (see eligibility criteria section 4.1).** Those patients who progressed on enzalutamide, apalutamide or darolutamide (at least 12 weeks of therapy) greater than 6 months prior to starting the IMP will enter the **Phase II enzalutamide resistance run in cohort** (section 1.4.2.1) to confirm resistance to enzalutamide; once progression on enzalutamide is confirmed they will enter the **Phase II reversal of enzalutamide resistance cohort** (section 1.4.2.2; if eligibility criteria met). Those patients who progressed on enzalutamide less than 6 months prior to starting the IMP will enter the **Phase II reversal of enzalutamide resistance cohort** (section 1.4.2.2).

12 Phase II enzalutamide resistance run in cohort

During the phase II enzalutamide resistance run in cohort patients will receive enzalutamide at 160mg orally once daily continuously.

13 Phase II reversal of enzalutamide resistance cohort

Once the MTD is reached, and if the MTD of AZD5069 is greater than 160 mg BD, two dose levels may be taken forward to a phase II reversal of enzalutamide resistance cohort. If the MTD is 160 mg BD or less, the MTD will be taken forward to a phase II reversal of enzalutamide resistance cohort.The dose and schedule to be taken forward will be determined by the Safety Review Committee (section 3.4).

14 Treatment group

15 Phase I safety run in cohort

Patients with histologically confirmed adenocarcinoma of the prostate that have progressed after either enzalutamide, apalutamide, darolutamide or abiraterone treatment (having received a minimum of 12 weeks enzalutamide or abiraterone).

16 Phase II cohorts

17 Phase II enzalutamide resistance run in cohort

Patients with histologically confirmed adenocarcinoma of the prostate that have progressed after either enzalutamide, apalutamide or darolutamide treatment (having

received a minimum of 12 weeks of treatment) **more than** 6 months prior to entry (day of starting IMP). Prior treatment with abiraterone is not an exclusion criteria.

18 Phase II reversal of enzalutamide resistance cohort

Patients with histologically confirmed adenocarcinoma of the prostate that have progressed after either enzalutamide, apalutamide or darolutamide treatment (having received a minimum of 12 weeks of treatment) **within** 6 months of trial entry (day of starting IMP). Prior treatment with abiraterone is not an exclusion criteria.

19 Trial timelines and accrual rate

Up to approximately 86 patients will be enrolled into this phase I/II trial, with up to 36 patients in the phase I safety run in cohort depending on number of patients required to determine the RP2D and up to 50 patients in the phase II study. We predict around 50% of these patients will enter the phase II enzalutamide resistance run in cohort first. The anticipated accrual rate for this trial is 3-6 patients per month across 4 centres.

20 INTRODUCTION

21 Background

22 Prostate cancer

Prostate adenocarcinoma is the most common male cancer in the Western World with over 570,000 new cases annually and an estimated 94,000 deaths in Europe and 32,050 deaths in the United States in 2008 (1). Surgery and/or radiotherapy can cure early PC. However, 30% of patients recur and >20% of patients present with advanced disease (2). The current standard of treatment is androgen ablation therapy. Despite initial robust responses, nearly all patients with advanced disease progress to fatal CRPC. Mitoxantrone was the first chemotherapy to show a palliative benefit for patients with CRPC and was approved by the food and drug administration (3). In 2003, the TAX327 trial demonstrated a survival advantage for patients treated with docetaxel compared to mitoxantrone (4). Until recently, cytotoxic chemotherapy had been the only therapy shown to improve survival for patients with CRPC. However, in the last 5 years, five novel treatments have shown survival benefits including sipuleucel-T, abiraterone, alpharadin, cabazitaxel and enzalutamide (5-8). Despite these newer therapies resistance to treatment remains common and new strategies for the treatment of mCRPC are needed to improve patient outcome.

23 Neutrophil to Lymphocyte ratio

A high neutrophil to lymphocyte ratio (NLR) is associated with poor overall survival from solid tumours (9). Interestingly in mCRPC patients with a high NLR was not only associated with poorer prognosis, it was also associated with a significantly poorer response rate to abiraterone and to cabazitaxel (10, 11). Overall, 49% of patients with an NLR ≤ 5 responded to abiraterone (PSA decline ≥50% below baseline maintained for ≥3 weeks) compared to 16% of patients with an NLR > 5 (p=0.01) with

these data being reproduced in independent test and validation sets from the Princess Margaret and the Royal Marsden Hospital (10). In the TROPIC trial evaluating cabazitaxel vs mitoxantrone, a high NLR was not only prognostic, but also associated with a significantly lower response rate to cabazitaxel; in addition, a reduction in NLR following cabazitaxel treatment was associated with an improved overall survival (11). In these and further studies, we have shown that this association is independent of therapeutic corticosteroid administration. We have hypothesized that patients with a high NLR have a high peripheral MDSCs counts and increased intratumoral MDSCs counts.

24 Myeloid derived suppressor cells

Activating the immune system has emerged as a promising way to treat cancer. Many cancers including prostate cancer alter the maturation of normal monocytes by secreting paracrine and endocrine factors, leading to the production of granulocytic MDSCs (gMDSCs) and monocytic MDSCs (mMDSCs) (12). gMDSCs are implicated in driving tumour growth and treatment resistance, and are defined as CD11b+ CD14- CD33+ CD15+ cells (12). mMDSC play a central role in immunosuppression and the subsequent failure of cytotoxic T-Cells to mount an efficient response to prostate cancer cells (13). Preclinical transgenic PTEN null mouse models indicate that MDSCs are recruited into tumours across a chemokine gradient (CXCL1 and CXCL2) through CXCR2 signalling (14). MDSCs secrete factors including IL-8, IL-6 and IL-1RA that fuel tumour growth by inhibiting senescence and driving proliferation (14). Increased circulating MDSC are also found in patients with prostate cancer, resulting in an elevated NLR (13, 15). High circulating MDSC counts are associated with more advanced disease and worse survival from CRPC (13, 15). Prostate cancer patients also have increased circulating IL-6 and IL-8, which correlate with increased circulating MDSCs (15). Moreover, increased MDSCs correlate with poor prognostic biomarkers including elevated lactate dehydrogenase (13). Following prostatectomy in patients with locally advanced disease, circulating MDSC counts decrease significantly indicating that these are generated by tumour derived endocrine/paracrine factors (16). Overall, these data indicate that MDSC play a critical role in fuelling prostate cancer cell proliferation and survival. Critically, studies in the transgenic PTEN knockout mouse model indicate that MDSC recruitment results in taxane treatment resistance; this was reversed when MDSC recruitment into tumour was inhibited by treating the mice with a CXCR2 antagonist (14). Patients treated with docetaxel in an adjuvant setting, with increased number of CD33+ infiltrates did not respond to the treatment (15).

25 Overview of enzalutamide

Investigators should be familiar with the current U.S enzalutamide (formerly MDV3100) Full Prescribing Information.
Enzalutamide is a small molecule that binds the androgen receptor (AR) and suppresses the androgen receptor-signalling axis. Enzalutamide slows growth and induces cell death in bicalutamide resistant cancers via three complementary mechanisms of action:

- Inhibiting testosterone binding to the AR
- Inhibiting nuclear translocation of the AR
- Preventing binding of the AR to DNA

26 Pre-clinical experience with enzalutamide

Enzalutamide has been studied extensively in humans. For full details of the pre-clinical experience with enzalutamide please refer to the U.S enzalutamide Full Prescribing Information.

27 Pharmacokinetics and drug metabolism of enzalutamide in animals

In mice, rats, and dogs, oral enzalutamide had a $t_{½}$ of approximately 0.25 to 3 days. The $t_{½}$ did not appear to be affected by dose; however, the bioavailability appeared to decrease with increasing dose. *In vitro* drug metabolism studies suggest that enzalutamide undergoes very slow rates of metabolism. Plasma protein binding of enzalutamide in human plasma ranged from 97% to 98% and was similar in mice, rats, rabbits, and dogs. *In vitro* drug metabolism studies suggest that enzalutamide may have the potential to induce cytochrome P450 (CYP) 3A4 and to directly inhibit CYP1A2, CYP2B6, CYP2C8, CYP2C9, CYP2C19, CYP2D6, and CYP3A4/5. In consideration of time dependent inhibition data, a metabolite of enzalutamide may inhibit CYP1A2.

28 Toxicology and safety pharmacology summary of enzalutamide

The safety of enzalutamide (160mg daily) has been investigated in two large multinational phase III trials in patients with metastatic castration resistant prostate cancer that had either received docetaxel chemotherapy or where chemotherapy naïve.

The phase III AFFIRM (NCT00974311) study investigated the efficacy and safety of enzalutamide (160mg daily) in patients with progressive castration resistant prostate cancer previously treated with docetaxel chemotherapy (8). The rates of adverse events were similar in patients treated with enzalutamide and placebo. The enzalutamide group had lower incidence of grade 3 adverse events (45.3%) when compared with placebo group (52.1%) (8). There was an increased incidence of headache, musculoskeletal pain, fatigue, diarrhoea and hot flushes at all grades in the enzalutamide group when compared to placebo group. Cardiac disorders were noted in 6% of patients receiving enzalutamide and 8% of patients receiving placebo. The incidence of hypertension and increased blood pressure was 6.6% in the enzalutamide group compared to 3.3% in the placebo group. Extensive cardiac monitoring demonstrated no clinically relevant differences in heart rate, atrioventricular conduction, cardiac depolarization, or effect on cardiac repolarization as determined by means of the QTc between enzalutamide and placebo groups (8). There was no evidence to suggest the development of metabolic syndrome between the enzalutamide group and placebo group with no obvious intergroup increase in hyperglycaemia, weight gain, hyperlipidaemia, or glucose intolerance. Liver-function

abnormalities were reported as adverse events in 1% of patients receiving enzalutamide and in 2% of those receiving placebo. As an inhibitor of the GABA gated chloride channel enzalutamide has the potential to cause seizure activity. Five patients (0.6%) of those in the enzalutamide group were reported to have had a seizure compared to no reports in the placebo group. One case of status epilepticus required medical intervention and the other four cases were self-limiting. A number of these cases had potentially predisposing factors including brain metastasis (2 cases), post lidocaine infusion (1 case), brain atrophy (1 case) and previous alcohol excess (1 case) (8). Overall, enzalutamide was well tolerated in the AFFIRM trial.
The phase III PREVAIL (NCT01212991) study investigated the efficacy and safety of enzalutamide (160mg daily) in chemotherapy naïve patients with progressive metastatic prostate cancer who had progressed on androgen deprivation therapy (17). Grade 3 or higher adverse events were reported in 43% of patients in the enzalutamide group compared to 37% in the placebo group. Most common toxicities (>20% of patients) were fatigue, back pain, arthralgia and constipation (17). The most common adverse event leading to death was disease progression and general deterioration, with similar incidences between the two groups. After correction for length of treatment exposure, events with a higher rate in the enzalutamide group than in placebo group were hot flush (14 vs 12 events per 100 patient-years), hypertension (11 vs 7 events per 100 patient-years), and falls (11 vs 9 events per 100 patient-years) (17). Hypertension was the most common grade 3 or higher event in the enzalutamide group (7%) compared to the placebo group (2%). Atrial fibrillation was the most common cardiac event (Enzalutamide group – 2%; Placebo group – 1%). There was a single seizure reported in each group and enzalutamide was not associated with hepatotoxicity (17). Consistent with the AFFIRM study, Enzalutamide was well tolerated in this population.

29 Clinical experience of enzalutamide

The efficacy of enzalutamide (160mg daily) has been compared to placebo in two large phase III multinational trials of patients with metastatic castration resistant prostate cancer that had either received docetaxel chemotherapy or were chemotherapy naïve.
In the phase III AFFIRM study 1199 patients who had castration resistant prostate cancer and had received docetaxel chemotherapy were randomised (2:1 ratio) to receive either enzalutamide (800 patients; 160mg daily) or placebo (399 patients) (8). The median overall survival was 18.4 months (95% confidence interval [CI], 17.3 to not reached) in the enzalutamide group and 13.6 months (95% CI, 11.3 to 15.8) in the placebo group (Hazard ratio in the enzalutamide group 0.63; 95% CI, 0.53 to 0.75; P<0.001). Enzalutamide was superior to placebo with respect to all secondary outcomes including soft tissue response rate (29% vs 4%, P<0.001), quality of life response rate (43% vs 18%, P<0.001), time to PSA progression (8.3 vs 3.0 months; HR, 0.25; P<0.001), radiographic progression-free survival (8.3 vs 2.9 months; HR, 0.40; P<0.001), reduction in PSA level by 50% or more (54% vs 2%, P<0.001) and time to first skeletal-related event (16.7 vs 13.3 months; HR, 0.69; P<0.001) (8). Overall enzalutamide was well tolerated (see section 2.2.3).

In the phase III PREVAIL study 1717 patients who had castration resistant prostate cancer who had not received chemotherapy were randomised to receive enzalutamide (872 patients; 160mg OD) or placebo (845 patients) (17). The rate of radiographic progression-free survival at 12 months was 65% in the enzalutamide group and 14% in the placebo group (HR in the enzalutamide group, 0.19; 95% CI, 0.15 to 0.23; P<0.001) (17). At time of analysis 72% of patients in the enzalutamide group were alive compared to 63% in the placebo group (HR, 0.71; 95% CI, 0.60 to 0.84; P<0.001). The benefit of enzalutamide was shown with respect to all secondary endpoints compared to placebo group including time to initiation of cytotoxic therapy (HR, 0.35; P<0.001), time until the first skeletal-related event (HR, 0.72; P<0.001), complete/partial soft tissue response (59% vs 5%; P<0.001), time to PSA progression (HR, 0.17; P<0.001) and reduction in PSA level by 50% or more (78% vs 3%; P<0.001) (17). Similar to the AFFIRM study, enzalutamide was well tolerated in the PREVAIL study (see section 2.2.3).

30 Pharmacokinetics and drug metabolism of enzalutamide in humans

Enzalutamide was absorbed rapidly after oral administration, with the time to maximum plasma concentration (t_{max}) after a single dose typically occurring at 1-hour post dose. No major deviations from dose proportionality were observed over the dose range 30mg to 600mg. Due to the long $t_{½}$ (~5.8 days) it took approximately 1 month to reach steady state concentrations. With daily oral administration, enzalutamide accumulation was observed at steady state with a 8.3-fold higher exposure (steady state area under the curve [AUC]) relative to a single dose. Based on the mean peak to trough ratio, the average difference between the peak (C_{max}) and trough (minimum plasma concentration [C_{min}]) concentrations was 25%. As a result of the low daily fluctuations, plasma profiles at steady state resembled a constant infusion. The C_{min} values in individual patients remained constant beyond Day 28 of chronic therapy, suggesting time-linear PK once a steady state was achieved.

Enzalutamide is a strong CYP3A4 inducer and a moderate CYP2C9 and CYP2C19 inducer in humans. At steady state, enzalutamide reduced the plasma exposure to midazolam (CYP3A4 substrate), warfarin (CYP2C9 substrate), and omeprazole (CYP2C19 substrate). Concomitant use of enzalutamide with narrow therapeutic index drugs that are metabolized by CYP3A4 (e.g. alfentanil, cyclosporine, dihydroergotamine, ergotamine, fentanyl, pimozide, quinidine, sirolimus and tacrolimus), CYP2C9 (e.g. phenytoin and warfarin) and CYP2C19 (e.g. S-mephenytoin) should be avoided, as enzalutamide may decrease their exposure. If co-administration with warfarin cannot be avoided, conduct additional INR monitoring. Single 160mg oral dose of enzalutamide was administered alone or after multiple oral doses of gemfibrozil (strong CYP2C8 inhibitor). Gemfibrozil increased the AUC_{0-inf} of enzalutamide plus N-desmethyl-enzalutamide by 2.2-fold with minimal effect on C_{max}. Single 160mg oral dose of enzalutamide was administered alone or after multiple oral doses of itraconazole (strong CYP3A4 inhibitor).

Itraconazole increased the $AUC_{0\text{-}inf}$ of enzalutamide plus N-desmethyl-enzalutamide by 1.3-fold with no effect on C_{max}.

31 Overview of AZD5069

Investigators should be familiar with the current AZD5069 Investigator Brochure (IB).

AZD5069 is a highly selective, small molecule potent CXCR2 antagonist. It has not been tested extensively in cancer patients although a phase Ib/IIa study of the combination of AZD5069 in combination with durvalumab (MEDI4736) in patients with metastatic pancreatic ductal adenocarcinoma (NCT02583477) was recently completed; both AZD5069 as monotherapy and in combination with durvalumab is currently being evaluated in patients with advanced solid malignancies including squamous cell carcinoma of the head and neck (NCT02499328). This will provide further information on its tolerability in cancer patients. AZD5069 has been extensively tested in patients with pulmonary disease (asthma, COPD and bronchiectasis) with long-term tolerability.

32 Pre-clinical experience with AZD5069

AZD5069 is a highly selective, potent CXCR2 antagonist as determined in both binding and functional studies of the human receptor. It is a highly efficacious inhibitor of CXCR2-mediated calcium mobilisation, adhesion molecule expression and chemotaxis of human neutrophils in vitro. AZD5069 is also a potent and selective antagonist of the CXCR2 receptor in a number of other species and has demonstrated with similar potency clear inhibition of rodent, canine and non-human primate CXCR2 receptors. In a rat model of lipopolysaccharide (LPS) induced pulmonary neutrophilia, AZD5069 demonstrated a dose dependent inhibition of the neutrophilia. In safety pharmacology studies with AZD5069 investigating effects on electrocardiogram (ECG) waveform morphology or any biologically significant effects on blood pressure, heart rate or ECG intervals in the dog (doses up to 477 mg/kg) or effects on gastrointestinal (GI), renal/urinary respiratory, and central and peripheral nervous system function in the rat, the only compound-related effect was an inhibition of gastric emptying and intestinal motility at a dose level of 350 mg/kg. A clear No Observed Effect Level (NOEL) was identified at 60 mg/kg.

33 Pharmacokinetics and drug metabolism of AZD5069 in animals

The absorption, distribution, metabolism and excretion (ADME) of AZD5069 have been studied in vitro and/or in vivo in hamster, mouse, rat, dog, rabbit, Cynomolgus monkey and human. AZD5069 is highly bound to plasma protein (>96%) with some potential concentration dependence in rat and rabbit. Quantitative whole-body autoradiography (QWBA) in the rat confirmed rapid absorption and widespread distribution of [^{14}C] AZD5069. There was no evidence of an association with melanin. In pregnant females there was evidence of fetal exposure. No unique human phase I metabolites (for example, formed via oxidation) have been identified but a phase II glucose conjugate metabolite was identified at a low % of dose (<5.3%) in urine from

the human mass balance study (NCT01332903) that was not detected in other species. Only 1 phase I circulating human metabolite has been identified at levels >10% of AZD5069 and this has been synthesised as the authentic reference compound AZ13587715. Exposures to AZ13587715 have been monitored in rat, rabbit and Cynomolgus monkey toxicity studies. Both rabbit and Cynomolgus monkey but not rats have measurable levels of AZ13587715 and these two species have been shown to have significant exposures to AZ13587715 in these safety tests. In rats and Cynomolgus monkey, [^{14}C] AZD5069-related material was predominately eliminated in faeces except after IV dosing in the Cynomolgus monkey where urine predominated.
In vitro studies in oocytes indicated that AZD5069 was a substrate for a range of uptake transporters and was actively effluxed in CACO-2 cells (efflux ratio 57 at 0.85 μmol/L) most probably via the breast cancer resistance protein (BCRP) transporter. In vitro studies indicated that AZD5069 was not an inhibitor of P-glycoprotein (P-gp) or CYP1A2, CYP2A6, CYP2B6, CYP2C19, CYP2D6, CYP2E1 or CYP3A4 at concentrations of up to 100 μmol/L but inhibited CYP2C8 and CYP2C9 with IC_{50} values (concentration of drug causing half maximal inhibitory effect) of approximately 100 μmol/L and 53.8 μmol/L, respectively. Based on the current data, no clinically significant effects on other drugs through inhibition of these pathways by AZD5069 are expected. There was no compelling evidence of time-dependent inhibition but there was a concentration dependent induction of CYP2B6 activity and P-glycoprotein (P-gp; MDR1) transporter mRNA in human hepatocytes. Studies using microsomes and heterologous expressed human cytochrome P450 enzymes indicate that the human CYP mediated metabolism of [^{14}C] AZD5069 involves CYP3A and CYP2C9. The major circulating human metabolite AZ13587715 did not inhibit the CYP1A2, CYP2C9, CYP2C19, CYP2D6 and CYP3A4 (all IC_{50} values >40 μM) and did not induce CYP1A2, CYP2B6 or CYP3A4, as measured by mRNA in the HepaRG cell line at concentrations up to 30μM.

34 Toxicology and safety pharmacology summary of AZD5069

Single doses up to the maximum feasible dose of 350 mg/kg BD were tolerated in the monkey with the only notable effects being increases in C-reactive protein (CRP) and decreases in white blood cell (WBC) (mainly due to neutrophil) counts. Single doses up to 600 mg/kg were tolerated in the rat with no noteworthy findings reported.
In the 1-month and 6-month rat studies, enlarged mandibular lymph nodes were identified in both sexes at 40 mg/kg BD and above, with histological evidence of inflammatory changes (and abscessation in some animals), and increases in circulating WBC (including neutrophils). In the 6-month study, the abscesses led to the early termination of many high dose animals. Increases in myeloid cells in the bone marrow were evident at all dose levels and increases in spleen weight in some groups correlated with a dose-related increase in granulocytes. No NOEL was established in either study; however, effects at the low dose levels were considered to be consistent with NOAELs; 35 mg/kg BD and 10 mg/kg BD in the 1 and 6-month studies, respectively. In the 1-month and 9-month monkey studies, dose levels up to 262.5 mg/kg BD were well tolerated and resulted in compound-related increases in

the Myeloid to Erythroid ratio in the bone marrow associated with an increase in segmented (mature) granulocytes at all dose levels with clear evidence of a dose-relationship. Clinical pathology changes included increases in CRP, slight reductions in red cell parameters (1-month study only), and a reduction in albumin: globulin ratio. A NOAEL was established in both studies at 262.5 mg/kg BD.
AZD5069 was not genotoxic in the in vitro and in vivo studies conducted and AZD5069 has a low risk for phototoxic potential. Rat and rabbit embryofetal development studies have identified NOEL or NOAEL dose levels at acceptable safety margins to support inclusion of women of child bearing potential in future clinical studies. The major human metabolite, AZ13587715, has been qualified in monkey general toxicology studies and in the rabbit embryofetal development study. It was not genotoxic in either the Ames or mouse lymphoma tests.

35 Clinical experience of AZD5069

To date, AZD5069 has been studied in 8 completed Phase I clinical studies in a total of 255 healthy volunteers, of whom 202 received AZD5069 (single doses up to 200 mg; multiple doses up to 80 mg BD for up to 7 days and 100 mg BD for up to 6.5 days). Two 4-week studies have been conducted in patients: 87 patients with chronic obstructive pulmonary disease (COPD; GOLD stage II-III) (50 mg and 80 mg BD/placebo), and 52 patients with bronchiectasis (80 mg BD/placebo). A Phase II efficacy study in 640 patients with persistent uncontrolled asthma has also been completed (478 patients received AZD5069 at doses of 5 mg, 15 mg BD, or 45 mg BD, while 162 patients received placebo for 6 months). In addition, approximately 70% of patients enrolled during this first portion of the trial completed a planned optional 6-month safety extension. Even though that portion of the study was terminated early due to a lack of efficacy, a small (up to 8 patient) study involving comparison of neutrophils in sputum, serum, and bronchial tissue in subjects with neutrophilic asthma is ongoing (4 patients have been enrolled as of 01 April 2015).
In the 202 healthy volunteers who have received AZD5069 to date, no clinically significant adverse effects have been observed that are considered to be related to treatment. There was a reduction in blood neutrophil counts at doses of 5.45 mg and above, as expected from previous experience with CXCR2 antagonists. Eleven healthy volunteers were withdrawn from multiple dose treatment because of blood neutrophil counts below $1x10^9$/L in 2 consecutive samples within 48 hours (Studies NCT01100047 and NCT01051505). Three healthy volunteers in the same studies were withdrawn by the investigators because of a rise in CRP to >3x upper limit of normal (ULN). Mean decreases in blood neutrophil counts were seen in COPD patients (Study NCT01233232), as with the healthy volunteers, with 4 patients having study drug discontinued (below $1x10^9$/L in 2 consecutive samples within 48 hours) as a result. There was no evidence of any increase in infections that might have been related to these neutrophil drops. In the bronchiectasis study NCT01255592 a mean reduction in blood neutrophils of approximately 16% was seen but no patients were withdrawn because of persistent drops below $1x10^9$/L. More patients reported adverse events (AEs) on AZD5069 compared with placebo (23 of 26, compared with 16 of 26), and more patients withdrew because of AEs on AZD5069 (5, compared

with 0 on placebo). The number of infections reported in the treated and placebo groups, however, did not differ significantly (9 (34.6%) versus 8 (30.8%). Further, there was only one infectious SAE (an infectious exacerbation of bronchiectasis) in the AZD5069 treatment group; this was deemed unrelated to the investigational product by the reporting investigator.
In the asthma study NCT01704495 longer term dosing (up to 12 months) of AZD5069 at 5 to 45 mg BD revealed no new safety signals. Among the 161 patients in the 6-month safety analysis set receiving AZD5069 at 45 mg BD, 4 discontinued the drug due to neutrophil counts $1x10^9$/L; none were fewer than $0.5x10^9$/L. Also, the overall rate of serious infections (defined by SAE or administration of IV antibiotics) in this 640 patient trial was <2.5% and did not differ significantly among treatment groups. Three of these events occurred in the highest dose AZD5069 treatment group. The effect of AZD5069 on sputum neutrophils was addressed as a primary objective in the bronchiectasis patient study NCT01255592. There was a statistically significant reduction in sputum neutrophils of 69% (one sided p-value=0.002) after 4 weeks' treatment with AZD5069 compared with placebo. No effect of AZD5069 on clinical variables such as lung function or diary card data was seen in this study; no efficacy variables were included in the COPD patient study NCT01233232. The effect of AZD5069 on the rates of severe asthma exacerbations was assessed as a primary objective in the asthma study NCT01704495. No statistically significant difference in these rates was observed in groups of patients treated with AZD5069 at BD doses of 5, 15, or 45 mg. A 12-month analysis of the rate of severe exacerbations was consistent with the 6-month results. No clear benefit was observed in any of the AZD5069 treatment groups with respect to hospitalization rates/total number of days of hospital admissions, severity of symptoms, pulmonary function, or quality of life.

Recently, Kirsten and colleagues investigated AZD5069 in a population of patients with chronic obstructive pulmonary disease (COPD) (18). Adverse events (AEs) were similar in patients receiving placebo (31%), 50mg AZD5069 (33%) and 80mg AZD5069 (21%) (18). AEs leading to discontinuation of treatment occurred in 3 (10%) patients in the placebo group, 5 (17%) patients in the AZD5069 50 mg group and 2 (7%) patients in the AZD5069 80 mg group. Reasons for treatment discontinuation included decrease in neutrophils (neutrophil count $<1.0 \times 10^9$/L in two blood samples, 24 hours apart) (50mg AZD5069, 10%; 80mg AZD5069, 4%), influenza, cardiac failure, lower respiratory tract infection, nasopharyngitis and pyrexia (18). Reduction in neutrophil count was not associated with increased risk of infection (Placebo, 17%; 50mg AZD5069, 10%; 80mg AZD5069, 4%). Two non-fatal serious AEs were reported during the study – one case of atrial fibrillation was seen in a patient receiving AZD5069 50 mg and a severe exacerbation of COPD was reported in one patient receiving AZD5069 80 mg. No AE-related deaths were reported. No clinically important AZD5069-related abnormalities in clinical chemistry or urinalysis were reported during the study; except for an expected reduction in blood neutrophil counts. Furthermore, no cases of syncope or hypotension were reported, and no clinically relevant effects on blood pressure or pulse rate were observed.

Adverse events have been reported for 109 patients who received AZD5069 in two oncology studies as of the data cut-off for the IB (07 July 2019). In NCT02499328, a study enrolling patients with advanced solid tumours including squamous cell carcinoma of the head and neck, 64 patients received AZD5069 40 mg BD; 59 of these patients receiving AZD5069 in combination with durvalumab (1.5g, q4w). Twenty-five patients received AZD5069 80 mg BD; 24 of these patients received AZD5069 in combination with durvalumab. In NCT02583477, a study enrolling patients

with metastatic pancreatic ductal adenocarcinoma, 20 patients received AZD5069 80 mg BD in combination with durvalumab (1.5g, q4w).

Adverse events considered by the investigator to be related to study treatment were reported in 80 (73.4%) patients. The most common AEs were fatigue (42, 38.5%), decreased appetite (39, 35.8%), and nausea (31, 28.4%), each of which occurred in ≥20% of patients at all doses. The most frequently reported causally-related AEs (i.e., those reported in ≥10% of patients overall) were fatigue (25, 22.9%), decreased appetite (22, 20.2%), neutropenia (17, 15.6%), nausea (13, 11.9%), neutrophil count decreased (12,11.0%) and diarrhoea (11, 10.1%). The most frequent causally-related AEs of CTCAE ≥Grade 3 were neutropenia (12, 11.0%), fatigue and neutrophil count decreased (5, 4.6% each). Causally-related SAEs were reported in 10 (9.2%) patients. In Study NCT02499328, neutrophil count decreased was reported more frequently at the 80 mg BD dose (6/25, 24%) than the 40 mg BD dose (4/64, 6.3%).There was no fatal AE that was considered to be treatment-related. Four (3.7%) patients experienced causally-related AEs that led to treatment discontinuation; two of which were due to treatment-related neutropenia.

36 Pharmacokinetics and drug metabolism of AZD5069 in humans

AZD5069 is rapidly absorbed with a t_{max} of ~2 hours during fasting conditions. Food reduces the peak concentration but total exposure (AUC) is unchanged. The plasma concentration declines with an initial half-life of 4 hours and a terminal half-life of 11 hours. Steady state is achieved in 2 to 3 days with no major time dependency in PK observed. Systemic exposure is proportional to the dose following both single and repeated BD dosing. The apparent volume of distribution is ~53L and the total plasma clearance is ~8L/h.
AZD5069 is metabolised by CYP3A4 and CYP2C9 enzymes. Less than 10% of the drug is renally excreted. Co-administration with ketoconazole (a strong inhibitor of CYP3A4) resulted in increased exposure of 2.1-fold (AUC) and 1.6 –fold (C_{max}). Japanese subjects seemed to have similar or higher exposure than Caucasian subjects and elderly subjects had higher exposure than young subjects. The exposure in COPD patients was similar to that of healthy volunteers. During the 6-month Phase II asthma study, plasma exposure of AZD5069 was approximately proportional to the dose in the range studied (5 to 45 mg BD) and the morning pre-dose plasma levels were consistent over the time period investigated (1 week to 6 months). Concentrations of major metabolite AZ13587715 were proportional to AZD5069.

37 Rationale for the proposed trial

Taken together these pre-clinical and clinical data support the investigation of treatments that oppose MDSC recruitment into tumours, and specifically the targeting of CXCR2 in patients with mCRPC. We now hypothesize that CXCR2 blockade in mCRPC patients will decrease intratumoral infiltration of MDSCs, decrease tumour cell survival and growth, impact circulating MDSC counts and reverse acquired endocrine treatment resistance.

38 TRIAL DESIGN

39 Clinical trial objectives and endpoints

40 Primary objectives and endpoints

Phase I safety run in cohort

Primary objective	Endpoint
To identify the safety and tolerability of enzalutamide and AZD5069 when given in combination continuously.	To identify the dose-limiting toxicities (DLTs), estimate the maximum tolerated dose (MTD) and identify the RP2D of AZD5069 administered in combination with enzalutamide at 160mg OD.

Phase II reversal of enzalutamide resistance cohort

Primary objective	Endpoint
To estimate the antitumour activity of AZD5069 in combination with enzalutamide as measured by response rate.	Antitumour activity will be defined by response rate on the basis of the following outcomes; if any of these occur, patients will be considered to have responded:<br>• PSA decline ≥ 50% criteria confirmed 4 weeks or later and/or,<br>• Confirmed soft tissue objective response by RECIST (v1.1) in patients with measurable disease and/or,<br>• ONLY for patients with detectable circulating tumour cell count (CTC) of ≥ 5/7.5ml blood at baseline, conversion of CTC <5/7.5ml blood nadir.<br>For disease progression (see section 3.6) the Prostate Cancer Working Group 2 (PCWG2) criteria and RECIST (v1.1) criteria will be used. Treatment failure will be defined as:<br>• Progression of soft tissue/visceral disease by RECIST (v1.1) and/or,<br>• Progression of bone disease by PCWG2 bone scan criteria and/or<br>• Progression of PSA by PCWG2 PSA criteria.

41 Secondary objectives and endpoints

Phase I safety run in cohort

Secondary objectives	Endpoint
To characterise the pharmacokinetics (PK) of AZD5069 and enzalutamide when administered in combination and assess drug interaction.	Determination of the plasma levels of enzalutamide and AZD5069 using validated assays
To characterise the pharmacodynamics (PD) of AZD5069 and enzalutamide when administered in combination.	Identify those patients with a neutrophil to lymphocyte ratio (NLR) ≥ 3 (at baseline) that convert to an NLR < 3 (blood nadir) with AZD5069 and enzalutamide in combination.<br>Identify those patients whose circulating myeloid derived suppressor cells (MDSCs) and intratumoral MDSCs reduce by 50% with AZD5069 and enzalutamide in combination.
To estimate the antitumour activity of AZD5069 in combination with enzalutamide as measured by response rate.	Antitumour activity will be defined by response rate on the basis of the following outcomes; if any of these occur, patients will be considered to have responded:<br>• PSA decline ≥ 50% criteria confirmed 4 weeks or later and/or,<br>• Confirmed soft tissue objective response by RECIST (v1.1) in patients with measurable disease and/or,<br>• ONLY for patients with detectable circulating tumour cell count (CTC) of ≥ 5/7.5ml blood at baseline, conversion of CTC <5/7.5ml blood nadir.<br>For disease progression (see section 3.6) the prostate cancer working group 2 (PCWG2) criteria and RECIST (v1.1) criteria will be used. Treatment failure will be defined as:<br>• Progression of soft tissue/visceral disease by RECIST (v1.1) and/or,<br>• Progression of bone disease by PCWG2 bone scan criteria and/or<br>• Progression of PSA by PCWG2 PSA criteria.

Phase II reversal of enzalutamide resistance cohort

Secondary objectives	Endpoint
To establish the maximum PSA decline at any point on trial and at 12 weeks for patients on AZD5069 and enzalutamide.	Maximal PSA decline at any time during the trial and PSA decline after 12 weeks (as per PCWG2 criteria) of combination treatment.
To estimate overall survival (OS) in these patients.	Overall survival will be measured from the date of AZD5069 addition to enzalutamide to the date of death (whatever cause). Survival time of living patients will be censored on the last date of patient is known to be alive or lost to follow up.
To estimate the radiologic progression free survival (rPFS) on the combination of AZD5069 and enzalutamide in these patients.	rPFS will be measured from the date of AZD5069 addition to enzalutamide until:<br>• Progression of soft tissue/visceral disease by RESIST and/or,<br>• Progression of bone disease by PCWG2 bone scan criteria and/or,<br>• Death of any cause<br><br>Patients withdrawn for any reason prior to radiological progression then the patient should be assessed until radiological progression has occurred. If however they have started another treatment then they will be censored at the start of the new treatment.
To assess the effects of AZD5069 and enzalutamide on the number of circulating tumour cells.	CTC fall by >30% will be expressed as the proportion of patients that have demonstrated a CTC fall of >30% after 12 weeks of combination treatment.
To further evaluate the safety and tolerability of the combination in patients who progress on enzalutamide.	Recording the population exposure to the AZD5069 and enzalutamide combination will summarise safety. Adverse events will be graded according to the CTCAEv4.0.
To further characterise the PD profile of AZD5069 and enzalutamide when administered in combination.	Identify those patients with a neutrophil to lymphocyte ratio (NLR) ≥ 3 (at baseline) that convert to an NLR < 3 (blood nadir) with AZD5069 and enzalutamide in combination.

Secondary objectives	Endpoint
	Identify those patients whose circulating myeloid derived suppressor cells (MDSCs) and intratumoral MDSCs reduce by 50% with AZD5069 and enzalutamide in combination.

42 Exploratory objectives

Phase I safety run in cohort

Exploratory objectives
Evaluating the effect of AZD5069 with and without enzalutamide on chemokine expression (such as IL-6, IL-8, CXCL-1, CXCL-2, CXCL-5), MDSC and tumour cell Ki67 in serial tumour biopsies using immunohistochemistry (IHC) and multi-colour immunofluorescence (MC-IF).
To evaluate the impact of CXCR2 inhibition in PTEN loss and PTEN wildtype cancers (immunohistochemical H score <30).
Evaluating the effect of AZD5069 treatment on circulating cytokine levels (such as GM-CSF, IL-6, IL-8, CXCL-1, CXCL-2 and CXCL-5) in whole blood using an enzyme-linked immunosorbent assay (ELISA).
Evaluating the effect of treatment on whole blood transcriptome profiles.
Evaluating the effect of treatment on white blood cells by whole blood immunophenotyping using fluorescence-activated cell sorting (FACS).
To correlate cell-free DNA (cfDNA) with response to treatment and disease progression.

Phase II reversal of enzalutamide resistance cohort

Exploratory objectives
To investigate the effect of AZD5069 and enzalutamide on chemokine expression such as CXCL-1, CXCL-2, CXCL-5, IL-6, IL-8 and tumour cell Ki67 expression in serial tumour biopsies using IHC and IF.
To study the effects of AZD5069 and enzalutamide on tumour biopsy infiltration by MDSC utilizing IHC and MC-IF.
To evaluate the impact of CXCR2 inhibition in PTEN loss and PTEN wildtype cancers (immunohistochemical H score <30).
To report the effect of AZD5069 and enzalutamide on circulating chemokine and cytokine levels (such as GM-CSF, IL-6, IL-8, CXCL-1, CXCL-2 and CXCL-5) in whole blood by ELISA.
To evaluate the effect of the combination on the NLR in whole blood.
To evaluate the combination’s impact on whole blood transcriptomes.

Exploratory objectives
To report the effect of the combination on circulating white blood cells by immunophenotyping through FACS.
To correlate cell-free DNA (cfDNA) with response to treatment and disease progression.

43 Definition of dose limiting toxicity

The dose limiting toxicity (DLT) and maximum tolerated dose (MTD) are defined using the National Cancer Institute (NCI) CTCAE Version 4.0. Please see section 9.3.1 for exceptions to this. DLTs will be primarily assessed during the 28-day DLT assessment window (Cycle 1)and are highly probably or probably related to either AZD5069 or enzalutamide:

- Grade 3 (or greater) non-haematological, non-hepatic major organ adverse event, excluding the following:
  - Grade 3 nausea, vomiting, or diarrhoea that resolves to grade 1 within 7 days with appropriate supportive care.
  - Grade 3 rashes that resolve rapidly upon discontinuation of drug with supportive measures.
  - Grade 3 laboratory abnormality that is asymptomatic and deemed by the investigator not to be clinically significant.
  - Alopecia of any grade
- Febrile neutropenia (fever of unknown origin without clinically or microbiologically documented infection) with Grade 4 neutropenia (absolute neutrophil count [ANC] <0.5 x 109/L and fever >38.3ºC or persistent fever of ≥ 38ºC for more than one hour)
- Infection (documented clinically or microbiologically) with Grade 4 neutropenia (ANC <0.5 x 109/L)
- Grade 4 neutropenia for seven days or more & see note
- Grade 4 thrombocytopenia associated with:
  - Duration of five days or more *see note
  - Active bleeding
  - Requirement for platelet transfusion
- Grade 4 anaemia
- One episode of fasting Grade 4 hyperglycaemia (>27.8mmol/l) or two episodes of asymptomatic fasting Grade 3 hyperglycaemia (>13.9 to ≤ 27.8mmol/L) on separate days, within 7 days, determined by laboratory blood glucose evaluation.
- Any Grade 3 (or greater) elevation of hepatic transaminases (ALT or AST) OR total bilirubin lasting >48 hours will be considered a DLT with the following exceptions:
  - For patients with elevated hepatic transaminase at baseline due to documented liver metastasis (i.e. ≤5xULN), hepatic transaminase >8x ULN for > 48 hours will be considered a DLT.
- Any case involving an increase in hepatic transaminase >3xULN and an increase in total bilirubin >2xULN, without any findings of cholestasis AND in the absence of other contributory factors (e.g. worsening metastatic disease or concomitant exposure to known hepatotoxic agent) is suggestive of potential drug induced liver injury according to Hy's Law and will be considered a DLT.
- Any other toxicity, which in the view of the investigators is considered to be a DLT, at any time during the study. These cases will be discussed at the safety review committee.

[&]Note: In the event of a Grade 4 neutropenia, a full blood count must be performed at least on Day 7 after the onset of the event to determine if a DLT has occurred. Continue to monitor the patient closely until resolution to Grade 1 or less.
*Note: In the event of a Grade 4 thrombocytopenia, a full blood count must be performed at least on Day 5 after the onset of the event to determine if a DLT has occurred. Continue to monitor the patient closely until resolution to Grade 1 or less.
In order to define DLT, patients should not be prophylactically prescribed growth factor support, antiemetics, anti-diarrhoeals or antipyretics prior to commencement of therapy. If a patient experiences any nausea and/or vomiting, diarrhoea, medical intervention should occur, including prophylactic administration of these agents for subsequent doses as indicated.
Management and dose modifications associated with the above adverse events are outlined in Section 5.3.
Should any change be made to the grade or causality of an AE during the trial that may alter its DLT status, the Drug Development Unit (DDU) must be informed immediately as this may affect dose escalation decisions.

44 Definition of maximum tolerated dose

If two out of up to six patients at the same dose level experience a DLT as defined above, the MTD will be determined as the dose level below. At least six evaluable patients are required to establish the MTD at a specific dose level for the combination of AZD5069 and enzalutamide.

45 Safety Review Committee and Independent Data Monitoring Commitee

46 Phase I safety run in cohort

To reflect that this is a phase I trial, rules for stopping investigational agent(s) due to toxicity should be enforced (Phase I safety run in cohort).
Regular weekly to 2-weekly safety reviews will be conducted between the Principal Investigator or delegate(s) from each investigational site and DDU Clinical Trials Manager or delegate. Decisions will be based on all relevant data available from all dose levels evaluated in the ongoing study including safety information, DLTs, toxicity data and available PK and PD data from evaluable patients.
A formal review of toxicity and dose escalation data will be undertaken by the SRC after accrual of at least 3 evaluable patients per cohort at each dose level during the dose escalation phase of the study.

The SRC will consist of:

- Chief Investigator or delegate (which can be the Principal Investigator)
- Principal Investigator or delegate from each investigational site
- DDU Pharmacovigilance Officer or delegate
- DDU Clinical Trials Manager or delegate
- Independent, Senior ECMC clinician (who is not CI)

Further internal or external experts may be consulted by the SRC as necessary.

The SRC will either meet face to face or via telephone conference and will review and assess all available safety data from the cohort, together with available pharmacokinetic and pharmacodynamics data to make a decision on the dose for the next cohort of patients. Any dose interruptions and reductions will be taken into account.

The decision may be to:

1. Proceed with dose escalation;
2. Expand the cohort to a maximum of 6 evaluable patients;
3. De-escalate the dose of AZD5069 or enzalutamide or both drugs within the combination;
4. Alter the schedule of AZD5069;
5. Define the MTD (where 6 evaluable patients are assessed);
6. Stop the study.

The outcome of the SRC meeting will be fully documented and circulated by email to the members of the SRC and investigational site staff. Patients must NOT be dosed at a higher dose level until after the SRC have agreed this and such communication has been received. During dose expansion, SRC meetings may be held as required for safety review (e.g. every 5-10 patients). Additional SRC meetings will be organised in the event of any safety concerns.

47 Phase II reversal of enzalutamide resistance cohort

An Independent Data Monitoring Committee (IDMC) will be set up to monitor the progress of the phase II study. It will comprise of a Chairperson and at least two further members with clinical or statistical expertise (at least one member must be a statistician).

The IDMC will meet in confidence at regular intervals, and at least annually and at the pre-planned interim analyses. A summary of findings and any recommendations will be produced following each meeting.

Specifically, the IDMC will further review the toxicity data and response data after 14 patients have completed stage 1 of the phase II reversal of enzalutamide resistance cohort. Recruitment may continue whilst the interim analysis of the primary outcome is being conducted in preparation for the IDMC. If two dose levels are expanded in two separate cohorts in the phase II study, the IDMC will review the data from stage 1 of each cohort separately to make a decision on whether one or both cohorts should proceed to stage 2.

If two dose levels are expanded in the phase II study, the dose level that will proceed to the next phase of evaluation will be determined based on antitumour activity, defined by response rate and duration of response, provided the safety profile is deemed acceptable by the IDMC.

48 Patient evaluability

During the Phase I part of the study DLTs will be assessed during the 28-day DLT assessment window (Cycle 1, Day 1 to Day 28) for patients treated at all other dose levels. The following patients will not be considered evaluable for DLTs and will be replaced:

- Patients who withdraw or are withdrawn from the study prior to completing the

DLT assessment window for any other reason than a DLT.

- Patients who miss >10 days of scheduled AZD5069 or enzalutamide dosing during the DLT assessment window for reasons other than a DLT. Patients will not make up missed doses of AZD5069 or enzalutamide.

During the Phase II reversal of enzalutamide resistance cohort patients that stop treatment with AZD5069 and enzalutamide within the first 12 weeks of treatment for reasons other than progression (Section 3.6) will be replaced.
During the phase II enzalutamide resistance cohort patients who become ineligible for the Phase II reversal of enzalutamide resistance cohort during treatment will be replaced.

49 Definition of disease progression (all stages)

For disease progression; RECIST (v1.1) and Prostate Cancer Working Group 2 (PCWG2) criteria will be used. A patient will be considered to have progressed on study if the following findings occur:

1) Progression of bone disease by PCWG2 criteria:

   Progression is defined as presence of 2 or more new bone metastasis as detected by bone scan as compared with baseline scan. However, if the 2 or more new lesions are detected at week 12, a confirmatory scan 6-12 weeks later with additional 2 or more new lesions will be required in order to qualify for disease progression. If on the confirmatory scan less than 2 additional lesions are detected, patients are classified as having stable disease.

2) Progression of soft tissue/visceral disease by RECIST (v1.1) (appendix 3):

   A patient will be determined to have progressed if they have progression of target lesions, clear progression of existing non-target lesions, or the appearance of one or more new lesions by RECIST (v1.1).

3) Progression of PSA by PCWG2 criteria:

   PSA progression is defined as a 25% or greater increase and an absolute increase of 2 ng/mL or more from the baseline value or a 25% or greater increase and an absolute increase of 2 ng/mL or more from the nadir if PSA decreases from baseline after treatment, which is confirmed by a second value obtained 4 or more weeks later. For the phase I safety run in and phase II reversal of enzalutamide resistance cohorts; PSA progression should only be defined on or after 12 weeks (3 cycles) of treatment. It is strongly recommended that in case of PSA progression in the absence of objective soft tissue or bone progression, patients will remain on study treatment. For the Phase II enzalutamide resistance run in cohort; PSA will be measured at 4 weekly intervals with those having PSA progression (by the above criteria) confirmed

by a second value 1 or more weeks later being confirmed as enzalutamide resistant.

4) Unequivocal evidence of clinical progression:

Discontinuation of treatment is discouraged unless the patient has progressed by biochemical and radiological measures. Nevertheless, a patient can be taken off trial treatment at the discretion of the treating clinician under the following circumstances:

- Marked escalation in cancer related pain that is assessed by the investigator to indicate the need for other systemic chemotherapy.
- Immediate need for initiation of new anticancer treatment, surgical or radiological intervention for complications due to tumour progression even in the absence of radiological progression.
- Marked deterioration in ECOG performance status to grade 3 or higher felt by the investigator to indicate clinical progression.
- Decision of investigator that in the best interest of the patient to discontinue trial treatment due to clinical progression.

50 Design of the clinical trial

This is a phase I/II proof of concept trial of the combination of the CXCR2 antagonist, AZD5069, and enzalutamide in patients with mCRPC. There are two parts to this study: a Phase I safety run in cohort and a phase II reversal of enzalutamide resistance cohort (including a enzalutamide resistance run-in cohort) (please refer to Figure 1 and 2).
The Phase I safety run in cohort (Figure 1) will investigate the combination of enzalutamide and AZD5069 to determine the dose of AZD5069 to be used for the Phase II reversal of enzalutamide resistance cohort. AZD5069 has been investigated in man and this safety run in will ensure there are no additional toxicities with dual therapy in castrate men (18). Higher AZD5069 doses may also be required in cancer patients, than those used to date. This study will pursue a 3+3 dose escalation design. The dose of enzalutamide will be fixed at 160mg OD. Higher doses of AZD5069 than those evaluated to date may be pursued if indicated by the acquired data. The AZD5069 starting dose will be 40mg BD with single dose escalations to 80mg BD, 120mg BD, 160mg BD and 320mg BD to determine the MTD. Intermediate dose levels may also be evaluated; for example, if the dose of 160mg BD is considered tolerable, but the dose of 320 mg is considered intolerable by the SRC, the intermediate dose of 240mg BD may be evaluated. For patients treated at AZD5069 dose levels 1-4, enzalutamide will be added after 2-weeks of AZD5069 monotherapy, to assess for the presence of drug-drug interactions. Since a drug-drug interaction was established from PK data from patients treated at dose levels 1-4, at subsequent dose levels, the two agents will be commenced concurrently without the

need for an AZD5069 monotherapy run-in (Figure 1). If the MTD is160 mg BD or higher, two dose levels with acceptable safety profiles as determined by the SRC (Section 3.4) may be taken forward as the RP2D for evaluation in the phase II study. Up to 25 patients will be treated at each RP2D in the phase II study (i.e. total of up to 50 patients) to explore their efficacy. Available PK and PD date may be taken into account when determining the dose level(s) to take forward to the phase II study.
The phase II reversal of enzalutamide resistance cohort(s) (Figure 2) will explore whether the addition of AZD5069 to enzalutamide reverses resistance to enzalutamide, as well as apalutamide or darolutamide in patients who progressed on these treatments within 6 months of trial entry. If the MTD for AZD5069 is greater than 160 mg BD up to two dose levels may be taken forward for evaluation in the Phase II study. This will pursue a two-stage Simon design to test the null hypothesis that P ≤ 0.05 (undesirable response rate) versus the alternative that P ≥ 0.25 (desirable response rate). For each RP2D, 14 patients will be treated in stage 1, the trial will be terminated if no patient responds. If at least 1 response is observed, the trial will go to stage 2 and recruitment will continue to a total of 25 patients. If the total number responding is less than or equal to 3 (in 25 patients), the RP2D will not be taken forward for further testing. This design has an expected sample size of 19.64 and a probability of early termination of 0.488. If the RP2D is actually not effective, there is a 0.033 probability of concluding that it is (type I error). If the RP2D is actually effective, there is a 0.101 probability of concluding that it is not (type II error). If two dose levels are expanded in the phase II study, the decisions to progress from stage 1 to stage 2 or for recruitment to be terminated, will occur independently in the two cohorts. At the time of adding dose level 5 (+/- 4b) to the phase 1 study, dose level 4 was already been established as safe and tolerable and phase II expansion at this dose (160 mg BD) had begun. Therefore, if another RP2D is established, there is likely to be no or little overlap in the recruitment phases of the two phase II expansions. If dose expansion is to occur at a second RP2D, and there are less than 8 remaining patients to be allocated to the expansion at dose level 4, patients will be systematically allocated to level 4 before expanding at a higher dose level; if 8 or more remaining patients are to be allocated to the expansion at dose level 4 when a second expansion cohort is to begin, then patients will be randomly allocated to the two arms.
Patients who progressed on enzalutamide, apalutamide or darolutamide treatment (having received a minimum of 12 weeks of treatment) within 6 months of trial entry (day of starting IMP) will enter the Phase II reversal of enzalutamide resistance cohort (Figure 2). Those patients who progressed on enzalutamide, apalutamide or darolutamide treatment (having received a minimum of 12 weeks of treatment) greater than 6 months before trial entry (day of starting IMP) will enter the Phase II enzalutamide resistance run in cohort (Figure 2) to confirm resistance. At which time point, if patients still meet the eligibility criteria, they will enter the Phase II reversal of enzalutamide resistance cohort.
If two dose levels are expanded in the phase II study, the dose level that will proceed to the next phase of evaluation will be determined based on antitumour activity,

defined by response rate and duration of response, provided the safety profile is deemed acceptable by the IDMC.
Up to approximately 86 patients will be enrolled into this phase I/II trial, with up to 36 patients in the phase I safety run in cohort depending on number of patients required to determine RP2D and up to 50 patients in the phase II. We predict around 50% of these patients will enter the phase II enzalutamide resistance run in cohort first. The anticipated accrual rate for this trial is 3-6 patients per month across 4 centres.

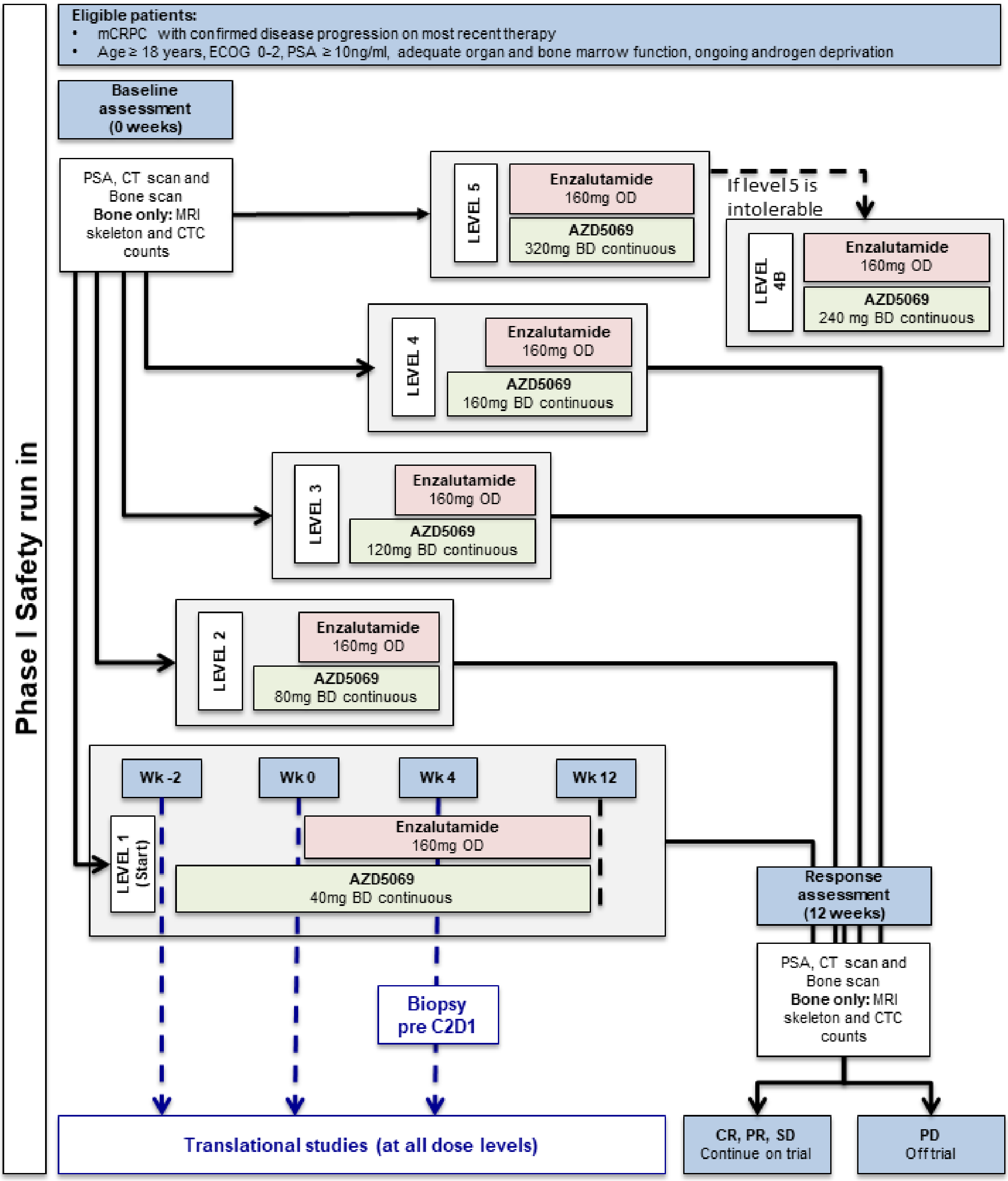

Figure 1: Phase I safety run in cohort

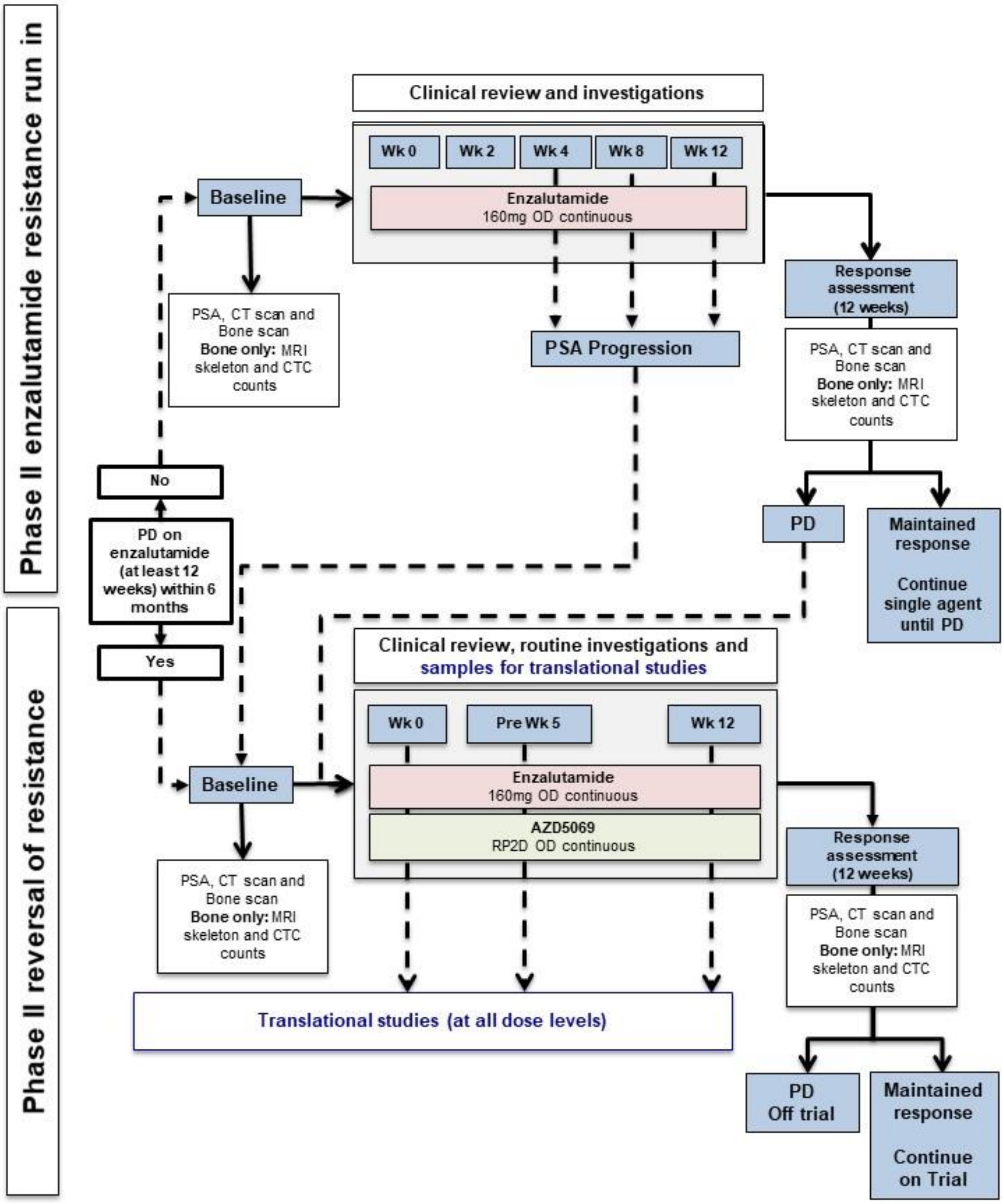

Figure 2: Phase II reversal of enzalutamide resistance cohort and Phase II enzalutamide resistance run in cohort

*Two RP2Ds may be taken forward to the Phase II study in two separate phase II reversal of enzalutamide resistance cohorts.

51 PATIENT SELECTION

52 Eligibility criteria

The patient must fulfil the eligibility criteria (listed in Sections 4.1.1 and 4.1.2).

53 Inclusion criteria:

1. Written informed consent and be capable of cooperating with treatment.
2. Age ≥ 18 years
3. Histologically confirmed adenocarcinoma of the prostate and with tumour tissue accessible for research analysis for this trial. Patients who have no histological diagnosis must be willing to undergo a biopsy to prove prostate adenocarcinoma.
4. Metastatic castration resistant prostate cancer.
5. Documented prostate cancer progression as assessed by the investigator with RECIST (v1.1) and PCWG2 criteria (Section 3.6) with at least one of the following criteria:
    a. Progression of soft tissue/visceral disease by RECIST (v1.1) and/or,
    b. Progression of bone disease by PCWG2 bone scan criteria and/or,
    c. Progression of PSA by PCWG2 PSA criteria and/or,
    d. Clinical progression with worsening pain and need for palliative radiotherapy for bone metastases.
6. PSA ≥ 10ng/ml.
7. Received prior castration by orchiectomy and/or ongoing luteinizing hormone releasing hormone agonist treatment.
8. Ongoing androgen deprivation with serum testosterone < 50 ng/dL (<2.0 nM).
9. Willing to have pre- and post-treatment biopsies to obtain proof of mechanism from translational studies. Archival tissue must be available for research analysis
10. Eastern Cooperative Oncology Group (ECOG) Performance Status of 0-2.
11. Documented willingness to use an effective means of contraception while participating in the study and for 6 months post last treatment dose as defined in Section 9.6.
12. Able to swallow the study drug.
13. All efforts should be made to discontinue steroid usage but up-to 5mg BD prednisolone (or equivalent) will be allowed.

14. Haematological and biochemical indices within the ranges shown below. These measurements must be performed within one week (Day -7 to Day 1) before the patient goes in the trial.

Laboratory Test	Value required
Haemoglobin (Hb)	≥ 9.0 g/dL
Absolute neutrophil count	≥ 1.5 x 10^9/L
Platelet count	≥ 100 x 10^9/L
WBC	≥ 3.0 x 10^9/L
Calculated creatinine clearance	≥ 50 mL/min (uncorrected value)
Serum bilirubin	≤ 1.5 x upper limit of normal (ULN) unless documented Gilbert's disease.
Alanine aminotransferase (ALT) and aspartate aminotransferase (AST)	≤ 2.5 x (ULN) unless raised due to known metastatic liver disease in which case ≤ 5 x ULN is permissible

15. **Phase I safety run in cohort ONLY**

Patients that have progressed after either enzalutamide, apalutamide, darolutamide or abiraterone treatment (having received a minimum of 12 weeks of enzalutamide, apalutamide, darolutamide or abiraterone treatment).

16. **1 Phase II enzalutamide resistance run in cohort ONLY**

Patients with histologically confirmed adenocarcinoma of the prostate that have progressed after either enzalutamide, apalutamide or darolutamide (having received a minimum of 12 weeks of enzalutamide, apalutamide or darolutamide) **more than** 6 months prior to entry (day of starting IMP). Prior treatment with abiraterone is not an exclusion criteria.

17. **Phase II reversal of enzalutamide resistance cohort ONLY**

Patients with histologically confirmed adenocarcinoma of the prostate that have progressed after either enzalutamide. apalutamide or darolutamide (having received a minimum of 12 weeks enzalutamide, apalutamide or darolutamide) **within** 6 months prior to entry (day of starting IMP). Prior treatment with abiraterone is not an exclusion criteria.

54 Exclusion criteria:

1. Surgery, chemotherapy or other anti-cancer therapy within 4 weeks prior to trial entry/randomization into the study (with the exception of enzalutamide, apalutamide or darolutamide). Any other therapy for prostate cancer, other than gonadotropin releasing hormone analogue therapy, such as progesterone, medroxyprogesterone, progestins or 5-alpha reductase inhibitors, must be discontinued at least 2 weeks before the first dose of the study drug.

2. Participation in another interventional clinical trial of an IMP within 4 weeks prior to trial entry. Participation in trials of licenced medications is allowed provided the medication is not a prohibited concomitant medication.

3. Prior limited field radiotherapy within 2 weeks and wide field radiotherapy within 4 weeks prior to trial entry.

4. Clinical and/or biochemical evidence of hyperaldosteronism or hypopituitarism.

5. History of seizures or other predisposing factors including, but not limited to, underlying brain injury, stroke, primary brain tumours, brain metastases and leptomeningeal disease, or alcoholism.

6. Use of drugs that are known potent/moderate CYP3A4 inhibitors or potent/moderate CYP3A4 inducers (with the exception of enzalutamide), potent inhibitors of CYP2C8, P-gp substrates with narrow therapeutic index, sensitive CYP2B6 substrates, warfarin or any other coumarin derivatives, BCRP-substrates that reduce blood neutrophils, Seville orange or grapefruit products, and any herbal medications should be avoided 4 weeks prior to trial entry (please see Appendix 5 and http://medicine.iupui.edu/clinpharm/ddis/table.aspx).

7. Malabsorption syndrome or other condition that would interfere with enteral absorption.

8. Any of the following cardiac criteria:
   - QT interval > 470 msec.
   - Clinically important abnormalities including rhythm, conduction or ECG changes (left bundle branch block, third degree heart block).
   - Factors predisposing to QT prolongation including heart failure, hypokalemia, congenital long QT syndrome, family history of prolonged QT syndrome, unexplained sudden death (under 40) and concomitant medications known to prolong QT interval.
   - Coronary artery bypass, angioplasty, vascular stent, myocardial infarction, angina or congestive heart failure (NYHA ≥ grade 2) in the last 6 months (see appendix 4 for NYHA scale).
   - Uncontrolled hypotension (systolic blood pressure < 90mmHg and or diastolic blood pressure < 50 mmHg).
   - Uncontrolled hypertension on optimal medical management

9. Clinically significant history of liver disease (Chlid-Pugh B or C, viral or other hepatitis, current alcohol abuse or cirrhosis).

10. Any other finding giving reasonable suspicion of a disease or condition that contraindicates the use of an investigational drug or that may affect interpretation of the results or renders the patients at high risk from treatment complications e.g. patients with a hypersensitivity to the active substance or any of the excipients.

11. Malignancy other than prostate cancer within 5 years of trial entry with the exception of adequately treated basal cell carcinoma.
12. Unresolved significant toxicity from prior therapy (except alopecia and grade 1 peripheral neuropathy).
13. Inability to comply with study and follow-up procedures.
14. Patients with predominantly small cell or neuroendocrine differentiated prostate cancer are not eligible.
15. Immunocompromised patients.
16. Active or uncontrolled autoimmune disease requiring corticosteroid therapy.
17. History of thromboembolic disease within 12 months of commencement of trial.
18. At high-risk because of non-malignant systemic disease including active infection and any serious concurrent illness.
19. Any known intolerance to enzalutamide, AZD5069 or to any constituents
20. Symptoms of COVID-19 and/or documented COVID-19 infection

55 Patient enrolment

All patients who provide informed consent including any screen failures must be entered onto the patient enrolment log provided by the DDU to the investigational site.

Before enrolling the patient in the trial, the Investigator or designated representative should confirm the patient is eligible to enter the trial. A registration form provided by the DDU should be completed prior to enrolment. To enrol a patient, email the completed registration form and any associated documents as instructed by the DDU, to the study-specific mailbox (see below). A trial number and dose level allocation will be entered on the registration form by the Clinical Trials Manager and emailed back to the Investigator site. The original registration form should be filed within the Investigator Trial File.

To enrol a patient please email the completed eligibility and enrolment form to:<br>**ACE@icr.ac.uk (Monday – Friday 09:00-17:00)**

56 TREATMENT

A multi-centre, proof of concept phase I/II trial of the CXCR2 antagonist AZD5069, administered in combination with enzalutamide, in patients with mCRPC.
The phase II reversal of enzalutamide resistance cohort and phase II enzalutamide resistance run in cohort will commence on completion of the phase I safety run in. Patients who have progressed on enzalutamide, apalutamide or darolutamide (at least 12 weeks of enzalutamide, apalutamide or darolutamide) greater than 6 months prior to trial entry will enter the Phase II enzalutamide resistance run in cohort. Those patients with confirmed enzalutamide resistance, who remain eligible, and those who progressed on enzalutamide, apalutamide or darolutamide (at least 12 weeks of enzalutamide, apalutamide or darolutamide) within 6 months of trial entry will enter the Phase II reversal of resistance cohort.

57 Dosing schedule/treatment schedule

58 Phase I safety run cohort

AZD5069 has been investigated in patients with chronic obstructive pulmonary disease (COPD) at dose levels of 50mg BD and 80mg BD (18). AZD5069 was well tolerated with adverse events (AEs) reported in 9 (31%), 10 (33%) and 6 (21%) patients in the placebo, AZD5069 50 mg and AZD5069 80 mg groups, respectively. AEs were generally mild or moderate in severity (18). The incidence of infections, the most commonly reported AE, was similar across the three groups (17%, 17% and 11% of patients in the placebo, AZD5069 50 and 80 mg groups, respectively). Blood neutrophil counts decreased on average from baseline by 14-40% and 13-36% in the AZD5069 50 mg and 80 mg groups, respectively, and 4 patients discontinued from the study due to decreased neutrophil count, 3 in the AZD5069 50 mg group and 1 in the 80 mg group (18). However, the safety and tolerability of AZD5069 in combination with enzalutamide at 160mg OD continuous dosing has not been established.

Multiple dose levels of AZD5069 (e.g. 40mg BD, 80mg BD, 120mg BD, 160mg, 320mg BD including intermediate dose levels such as de-escalation from 320 mg to 240 mg BD if 160 mg BD is tolerable and 320 mg BD is not tolerable) in combination with enzalutamide are planned. Enzalutamide will be administered at 160mg OD.

- From dose level 5 onwards, patients will start on the AZD5069 and enzalutamide concurrently on Cycle 1 Day 1. Dosing between patient 1 and 2 of each cohort will be staggered by one week; patient 2 and 3 will be able to commence on the study drugs on the same day. Dosing in subsequent cohorts can commence after all patients in the preceding dose level has completed the DLT period and the preceding dose level has been deemed safe by the SRC. If dose level 4 (160 mg BD) is deemed tolerable and dose level 5 (320 mg BD) is deemed not tolerable by the SRC, an intermediate dose level 4B (240mg BD of AZD5069) will be evaluated. Decisions on dose escalation or expansion will be made by the Safety Review Committee.

Once the MTD is determined in the phase I study, the optimum dose and/or schedule combination will be taken forward as the RP2D for the phase II reversal of enzalutamide resistance cohort. Two different dose levels of AZD5069 may be taken forward to the phase II study if the MTD of AZD5069 is greater than 160mg BD.

59 Phase II cohorts

60 Phase II enzalutamide resistance run in cohort

Patients who have documented progression (Section 3.6) and progressed on enzalutamide, apalutamide or darolutamide (having received a minimum of 12 weeks of enzalutamide, apalutamide or darolutamide) **greater than** 6 months prior to trial entry (day of starting IMP) will be eligible for the phase II enzalutamide resistance run in cohort. Patients will receive enzalutamide 160mg OD continuously until disease progression (PSA progression at 4 or 8 weeks; or full evaluation at 12 weeks). Those patients with confirmed disease progression (Section 3.6) and who remain eligible for the Phase II reversal of enzalutamide resistance cohort will begin combination treatment with AZD5069 at the RP2D in combination with ongoing enzalutamide 160mg OD continuously to explore if AZD5069 can reverse resistance to enzalutamide therapy.

61 Phase II reversal of enzalutamide resistance cohort

Patients who have documented progression (Section 3.6) and progressed on enzalutamide, apalutamide or darolutamide (having received a minimum of 12 weeks enzalutamide, apalutamide or darolutamide) **within** 6 months of trial entry (day of starting IMP) will be eligible for the phase II reversal of resistance cohort. Patients will receive AZD5069 at the RP2D in combination with enzalutamide 160mg OD continuously to explore if AZD5069 can reverse resistance to enzalutamide therapy.

62 Dose escalation scheme

63 Phase I safety run cohort

A minimum of 3 patients will be enrolled to Dose Level 1. If none of the first 3 patients experiences a dose limiting toxicity (DLT), dose escalation may proceed to Dose Level 2. If one instance of DLT (as defined in Section 3.2) is observed in three patients, up to six patients will be treated at that dose. If less than two of six patients at any dose level experience a DLT, dose escalation will occur to the next level. If two out of up to six (i.e. between two and six) patients experience a DLT dose escalation will stop and this dose will be defined as the maximum administered dose (MAD). Once this MAD is defined, the MTD (maximum tolerated dose) will be confirmed at the previous dose-level below the MAD. At least six evaluable patients are required to establish the MTD at a specific dose level. Dose escalation may only proceed following confirmation from the Safety Review Committee. If the initial dose level evaluated is not considered tolerable then a different schedule of administration of AZD5069 may be explored on the advice of the SRC.

Dose level	AZD5069	Enzalutamide	Maximum number of patients
-1	20mg BD (40mg total)	160 mg OD	3+3
1 (starting)	40mg BD (80mg total)	160mg OD	3+3
2	80mg BD (160mg total)	160mg OD	3+3
3	120mg BD (240mg total)	160mg OD	3+3
4	160mg BD (320mg total)	160mg OD	3+3
4B*	240mg BD (480mg total)	160mg OD	3+3
5	320mg BD (640mg total)	160mg OD	3+3

*Dose level 4B will only be evaluated if dose level 4 is deemed tolerable, but dose level 5 is deemed not tolerable by the SRC.

Table 1: Phase I safety run in dose escalation schedule

64 Dose Delays/Dose modifications and Adverse Event Managment

If a patient experiences a clinically significant and/or unacceptable toxicity including a DLT not attributable to the disease or disease-related processes under investigation, dosing will be interrupted or the dose reduced and supportive therapy administered as required.
If the toxicity resolves or reverts to ≤ CTCAEv4.0 Grade 1 within 14 days of onset, treatment with the combination of enzalutamide and AZD5069 may be restarted following agreement with the Chief Investigator.
For all other events, if the toxicity does not resolve to ≤ CTCAEv4.0 Grade 1 after 14 days, then the patient should be discontinued from treatment and observed until resolution of the toxicity.

65 Enzalutamide dose reduction/dose adjustment

Patients who experience Grade 3 or greater toxicity that is related to enzalutamide in the opinion of the investigator and that cannot be ameliorated by the use of adequate medical intervention should have their enzalutamide treatment interrupted until the toxicity improves to Grade 1 or lower severity. Patients may subsequently be restarted on study drug, including at a reduced dose of 120mg (3 capsules/tablets) of enzalutamide. No further dose reductions for enzalutamide will be allowed on this trial.
Enzalutamide has been associated with a small risk of seizures and hallucinations. Patients with known predisposing factors, including but not limited to, brain metastases, leptomeningeal spread of the cancer, a history of stroke or epilepsy, or

excess alcohol intake will be excluded from this trial. Patients who experience a seizure or unexplained loss of consciousness of any grade on trial while receiving treatment with enzalutamide will discontinue trial treatment (enzalutamide and AZD5069).

66 AZD5069 dose reduction/dose adjustment

AZD5069 has been well tolerated in healthy individuals and patients with pulmonary diseases. In the 202 healthy volunteers who have received AZD5069 to date, no clinically significant adverse effects have been observed that are considered to be related to treatment. There was a reduction in blood neutrophil counts but no increased incidence of infection between patients treated with AZD5069 and placebo. If a patient experiences a clinically significant and/or unacceptable toxicity including a DLT not attributable to the disease or disease related processes under investigation, dosing will be interrupted or the dose reduced and supportive therapy administered as required. If the toxicity resolves or reverts to CTCAEv4.0 grade ≤1 within 8 days of onset and the patient is showing clinical benefit, treatment with AZD5069 may be restarted. If the patient is still showing clinical benefit, but toxicity takes between 8 and 14 days to resolve or revert to CTCAEv4.0 grade ≤1, treatment with AZD5069 may be restarted using a lower dose as shown in Table 1.
Patients who are at the lowest possible dose i.e. Dose Level -1 or who have their dose previously reduced to Dose Level -1 (20mg BD) and who have demonstrated an acceptable response to the dose interruption may be permitted to restart at the discretion of the Investigator. For all other events, if the toxicity does not resolve to CTCAEv4.0 Grade ≤1 after 14 days, then the patient should be discontinued from treatment and observed until resolution of the toxicity.

67 Intra-patient dose escalations

Intra-patient dose escalation will be considered in patients progressing on a lower dose level if a higher dose level has been deemed to be safe by the SRC.

68 Dose interruptions due to COVID-19

If a patient tests positive for COVID-19 during the study, treatment will be interrupted until the patient becomes asymptomatic, has tested negative for the virus and it is deemed safe for them to continue on study. During treatment interruption the patient will be followed up regularly by phone.

69 Duration of treatment

Patients will receive study treatment until disease progression (Section 3.6) or unacceptable toxicity or patient withdrawal from the study, study completion or termination and in any case no treatment will be given beyond August 2022 due to the discontinuation of AZD5069. Patients with pure PSA progression in the absence of clinical or radiological progression should remain on trial. If a patient is withdrawn for any reason prior to radiological progression then the patient should be assessed

until radiological progression has occurred or a subsequent line of treatment is started.

A complete treatment cycle is defined as 28 days of uninterrupted continuous treatment with the study drug combinations. The first day that the combination of both enzalutamide and AZD5069 is given defines Cycle 1 Day 1. All treatment cycles have duration of 28 days. There will be no delays between cycles, i.e. study day 29 represents Cycle 2 Day 1. Patients treated at dose levels 1-4 in the phase I study were started on AZD5069 14 days (Cycle 1 Day -14) before enzalutamide (Cycle 1 Day 1), whilst patients treated at subsequent dose levels in the Phase I study will start AZD5069 and enzalutamide concurrently. In the phase II reversal of enzalutamide resistance cohort AZD5069 and enzalutamide will be given at the same time (Cycle 1 Day 1). In the phase II enzalutamide resistance run in cohort patients will receive enzalutamide only.

70 Concomitant medication and treatment

Non permissible concurrent medications/therapies include:
- Supplements or complementary medications (conventional multivitamins are allowed).
- Other approved or investigational systemic anticancer treatments, including chemotherapy, hormone therapy and immunotherapy.
- Other investigational drugs.
- Concurrent radionucleotide treatment.

Permissible concurrent medications/therapies include:
- Luteinizing hormone releasing hormone (LHRH) analogue to maintain a testosterone level <50g/dL should be administered in patients who have not undergone orchiectomy.
- Conventional multivitamins and minerals.
- Initiating bisphosphonates or denosumab therapy or adjusting bisphosphonates or denosumab dose/regime within 30 days prior to cycle 1 day 1 is prohibited. Patients on a stable bisphosphonate or denosumab regimen are eligible to continue.
- Blood transfusions and growth factor support as per standard of care and institutional guidelines. Granulocyte colony stimulating factors should not be used prophylactically during Cycle 1.
- Anticoagulant therapy: It is recommended that patients who are taking warfarin should be switched to the equivalent dose of LMWH.
- Single fraction palliative radiotherapy: May be used for the treatment of pain at the site of bony metastasis that were present at baseline, providing the investigator does not feel that this is an indication for the patient to discontinue treatment due to disease progression.
- Prophylactic antiemetics as required.

71 Enzalutamide

a) Medications (potentially) affecting enzalutamide exposure

CYP2C8 plays an important role in enzalutamide elimination and formation of its active metabolite. Strong inhibitors of CYP2C8 (please see: http://medicine.iupui.edu/clinpharm/ddis/table.aspx) should be avoided during enzalutamide treatment. CYP3A4 plays a minor role in metabolism of enzalutamide. CYP3A4 inhibitors, and CYP2C8 and CYP3A4 inducers can be co-administered with enzalutamide. For full details please refer to the U.S enzalutamide Full Prescribing Information.

b) Enzalutamide (potentially) affecting exposure to other medications

Enzalutamide is a potent enzyme inducer and increases the synthesis of many enzymes and transporters; therefore, interactions with many common medicinal products that are substrates of these enzymes of transporters is expected. The reduction in plasma concentrations can be substantial, and lead to lost or reduced clinical effect. There is also a risk of increased formation of active metabolites. Enzymes that may be induced include CYP3A4, CYP2B6, CYP2C9, CYP2C19, and uridine 5'-diphospho-glucuronosyltransferase (UGTs – glucuronide conjugating enzymes). The transport protein P-gp may also be induced along with other transporters as well (e.g. multidrug resistance associated protein 2 (MRP2), breast cancer resistance protein (BCRP) and the organic anion transporting polypeptide 1B1 (OATP1B1). Patients taking medicinal products that are substrates of CYP3A4, CYP2B6, CYP2C9, CYP2C19, UGT1A1, P-gp, BCRP, MRP2, organoic anion transporter 3 (OAT3) and organic cation transporter 1 (OCT1) (please see: http://medicine.iupui.edu/clinpharm/ddis/table.aspx) should be evaluated for possible loss of pharmacological effects (or increase in effects in cases where active metabolites are formed) during the first month of enzalutamide. Since androgen deprivation treatment may prolong the QT interval, the concomitant use of enzalutamide with medicinal products known to prolong the QT interval or medicinal products able to induce Torsade de pointes such as class IA (e.g. quinidine, disopyramide) or class III (e.g. amiodarone, sotalol, dofetilide, ibutilide) antiarrhythmic medicinal products, methadone, moxifloxacin and antipsychotics (not exhaustive) should be carefully evaluated. For full details please refer to the U.S enzalutamide Full Prescribing Information.

72 AZD5069

In patients treated with AZD5069, coadministration of drugs that are known potent or moderate CYP3A4 inhibitors, potent or moderate CYP3A4 inducers, P-gp substrates with narrow therapeutic index, sensitive CYP2B6 substrates, warfarin or any other coumarin derivatives, BCRP-substrates that reduce blood neutrophils, Seville orange or grapefruit products, and any herbal medications should be avoided (with the exception of enzalutamide).
For each patient enrolled in this study, the Investigator has to assess both the patient's medication history for any such products as well as the patient's anticipated need or likelihood to consume such products at any time throughout the study.

Restrictions apply starting 14 days prior to the first dose of AZD5069 and last for as long as the patient is treated with AZD5069 and until 24 hours after the last dose of AZD5069.
A list of the main potent or moderate CYP3A4 inhibitors, potent or moderate CYP3A4 inducers, P-gp substrates with narrow therapeutic index, sensitive CYP2B6 substrates, warfarin or any other coumarin derivatives, and BCRP-substrates that reduce blood neutrophils is shown in appendix 5 (please note this is not an exhaustive list). In addition, investigators should look at a frequently updated drug reference such as Lexicomp to see if any medicine they want to prescribe is on a list of drugs to avoid.
Herbal preparations/medications can be substrates, inhibitors, and inducers, similar to any registered medication. Herbal preparations are therefore not allowed throughout the study for patients administered AZD5069 alone or in combination with enzalutamide. These herbal medications include, but are not limited to dehydroepiandrosterone, ephedra (ma huang), Gingko biloba, ginseng, kava, saw palmetto, St. John's wort, and yohimbe.
Patients administered AZD5069 alone and in combination with enzalutamide must not consume Seville orange marmalade, Seville orange juice, grapefruit, grapefruit juice, grapefruit marmalade, or other Seville orange or grapefruit products. For full details please refer to AZD5069 IB.

<u>COVID-19 guidelines:</u>
It is not recommended to receive the COVID-19 vaccination during screening and the first two cycles of combination therapy (AZD5069 and Enzalutamide) during the Phase I safety run-in cohort or during the Phase II reversal of resistance component of the ACE trial due to the unknown interaction of the vaccine and AZD5069. However, COVID-19 vaccination can be considered outside of these times by the treating physician following careful discussion with the patient.

For those patients who are established on single agent enzalutamide and deemed clinically stable within the Phase II enzalutamide resistance run-in cohort then COVID-19 vaccination can be considered by the treating physician following careful discussion with the patient.

73 Corticosteroids

It is strongly recommended that patients considered for this trial be taken off any chronic low dose steroid treatments before entering the trial (see Section 4.1: Inclusion Criteria). In case of emergency then a short course of high dose steroids is allowed (<28 days) and glucose levels should be monitored. If chronic corticosteroids therapy is stopped, study treatment can only be started if PSA is not declining within the two weeks prior to study initiation.

74 PHARMACEUTICAL INFORMATION

75 Supply of enzalutamide and AZD5069

A complete certificate of analysis and a Qualified Person (QP) certification must be provided with each batch of IMP and be retained in the Investigator Trial File (ITF)/Pharmacy File.
All study drug will be packaged and labelled in accordance with local regulations and Good Manufacturing Practice, stating that the drug is for clinical trial use only and should be kept out of the reach and sight of children. The drug will be supplied, packaged, labelled and QP released by Catalent Pharma Solutions.
Enzalutamide and AZD5069 will only be dispatched to sites after receipt of confirmation that the regulatory checklist is complete.
For information on all IMP and re-ordering of supplies, contact the DDU Clinical Trials Manager responsible for the trial who will arrange further supplies.

76 Pharmaceutical data

77 Formulation of enzalutamide and AZD5069

Enzalutamide is presented in 40mg white to off white soft capsules or as yellow film-coated tablets supplied either as;
Capsules/tablets provided in a cardboard wallet incorporating a PVC/PCTFE/aluminium blister which holds 28 soft capsules/tablets. Each carton contains 4 wallets (112 soft capsules/tablets). Complete information can be found in the Summary of Product Characteristics.
Or in bottles containing 120 soft capsules. Complete information can be found in the Full Prescribing Information.AZD5069 is presented as 10mg and 40mg plain, beige, film-coated tablets packaged in bottles.

78 Storage conditions

All supplies must be stored in a secure, limited access storage area. The drug products should be stored in accordance with the labeling. Please refer to the current IB (AZD5069) or Full Prescribing Information (enzalutamide) for detailed information on storage conditions and stability.

79 Administration of AZD5069 and enzalutamide

Patients should be fasted for at least 2 hours before AZD5069 administration and continue to fast for at least 2 hours after. Water and liquids are allowed during this fasting period. Doses should be taken at approximately the same time every day.
The first dose of AZD5069 should always be taken in clinic, in the presence of the trial nurse.

80 Enzalutamide should be administered as a single daily dose of 160 mg (four 40 mg capsules/tablets).Enzalutamide and AZD5069 accountability

Accurate records of all IMP shipments, tablets/capsules dispensed, and all tablets/capsules returned must be maintained. This inventory record must be available for inspection at any time by members of the Drug Development Unit

(DDU). IMP supplies are to be used only in accordance with this protocol and under the supervision of the Investigator.
The Investigator delegates responsibility for IMP management to the Pharmacist. The Pharmacist undertakes not to destroy any unused IMP unless directed to by the DDU. Any unused IMP must be destroyed according to hospital procedures and properly accounted for using the IMP Destruction Form and also on the IMP Accountability Record. During the course of the trial the Clinical Trials Manager will check the numbers of tablets/capsules of enzalutamide and AZD5069 shipped to the centre, the number used and the number destroyed or returned. The pharmacy will give an account of any discrepancy.

81 INVESTIGATIONS SCHEDULE

82 Pre-treatment evaluations

Details of all evaluations/investigations for enrolled patients, including relevant dates, required by the protocol must be recorded in the medical records so that the eCRF can be checked against the source data.
Please also refer to the tabulated Schedule of Assessments in Section 7.5.

83 Obtaining written informed consent

Written informed consent must be obtained from the patient before any protocol-specific procedures are carried out and within four weeks before the patients first dose of the IMP. Initial discussion and signing of the informed consent must **not** occur on the same day, as the patient must be given time to think about their commitment to the trial.
Only the Principal Investigator (PI) and those Sub-Investigator(s) delegated responsibility by the PI, and have signed the Study Site Delegation Log, are permitted to gain informed consent from patients and sign the consent form. All signatures must be obtained before the occurrence of any medical intervention required by the protocol (ICH GCP 4.8.8 and 8.3.1.2). The date of the signatures of both the patient and the PI/Sub-Investigator should be the same.

- The PI or the Sub-Investigator must inform the patient about the background to, and present knowledge of the normal management of their disease (in case the patient does not go on the trial) and the IMP and must also ensure that the patient is aware of the following points. The known toxicity of the IMP and the possibility of experiencing side effects.
- That the IMP is new and that the exact degree of activity is at present unknown, but that treating him/her will contribute to further knowledge.
- The potential dangers of the patient’s partner becoming pregnant and he has been given information about appropriate medically approved contraception (refer to Section 9.6).
- That the patient may refuse treatment either before or at any time during the trial and that refusal to participate will involve no penalty or loss of benefits to which they are otherwise entitled.

- Whom to contact for answers to pertinent questions about the research and their rights, and also who to contact in the event of a research-related injury.

A copy of the consent form and patient information sheet must be given to the patient to keep and the original consent form and patient information sheet, must be filed in the Investigator Trial File (ITF) (unless otherwise agreed that the original consent form will be filed in the medical records and the copies kept in the ITF).

84 Evaluations within four weeks (28 days)

The following must be performed/obtained **within the four weeks before** the patient receives the first dose:

- Informed consent (as detailed in Section 7.1.1);
- Demographic details;
- Medical history including prior diagnosis, prior surgery, prior treatments and any residual toxicities, concomitant diseases, concomitant treatment and allergies;
- Acquisition of archival tissue;
- Radiological disease assessments: Computer Tomography (CT) scan of thorax, abdomen and pelvis; or Magnetic Resonance Imaging (MRI) scan of abdomen and pelvis, with CT chest and bone scan (if indicated); In patients with no measurable disease by routine radiological assessment, whole body MRI scan is recommended to determine whether disease is measurable.
    - Radiological measurements must be performed within four weeks before the patient receives the first dose.
- Urinalysis – specific gravity, pH, glucose, erythrocytes, protein, ketones and nitrites.
- Electrocardiogram (ECG): Resting 12-lead electrocardiogram (ECG), conducted in triplicate taken up to 5 minutes apart, with manual calculation of QT interval (QTcF).

Note that all adverse events (AEs), including serious adverse events (SAEs), must be monitored and recorded in the eCRF from the time the patient consents to any protocol-specific procedure (Refer to Section 9).

<u>COVID-19 guidelines:</u>
It is not recommended to receive the COVID-19 vaccination during screening and the first two cycles of combination therapy (AZD5069 and Enzalutamide) during the Phase I safety run-in cohort or during the Phase II reversal of resistance component of the ACE trial due to the unknown interaction of the vaccine and AZD5069.

However, COVID-19 vaccination can be considered outside of these times by the treating physician following careful discussion with the patient.

For those patients who are established on single agent enzalutamide and deemed clinically stable within the Phase II enzalutamide resistance run-in cohort then COVID-19 vaccination can be considered by the treating physician following careful discussion with the patient.

85 Evaluations within one week (7 days)

The following must be performed/obtained one week before the patient receives the first dose:

- **Clinical disease measurements:** If applicable (i.e. patients with clinically assessable disease).
- **Physical examination:** Cardiovascular, respiratory, abdominal, central and peripheral nervous system, dermatological and any other system which might be relevant to the patient’s medical history.
- **Vital signs:** Height, weight, body surface area (BSA), WHO performance status, temperature, supine blood pressure (BP), capillary blood glucose, pulse oximetry and pulse rate.
- **Laboratory blood tests to confirm eligibility:**
  - Haematology – haemoglobin (Hb), white blood cells (WBC) with differential count (neutrophils and lymphocytes) and platelets.
  - Biochemistry – sodium, potassium, adjusted calcium, phosphate, magnesium, urea, creatinine, total protein, albumin, bilirubin, alkaline phosphatase (ALP), alanine transferase (ALT) and aspartate aminotransferase (AST).

    In addition the following should be determined:

    - Thyroid function including TSH (thyroid stimulating hormone) and T4
    - Follicle stimulating hormone (FSH)
    - Fasting blood glucose
    - Glycosolated haemoglobin (HbA1c),
    - Serum testosterone
    - CRP
- **Collection of blood sample for PD analysis**

- Phase I safety run in cohort: Baseline blood samples will be collected (Refer to Section 8.3.1).
- Phase II reversal of enzalutamide resistance cohort: Baseline blood samples will be collected (Refer to Section 8.3.2).
- Phase II enzalutamide resistance run in cohort: PD samples not required.

- **Tumour serum markers**: PSA.
- **Tumour biopsy:** A tumour biopsy will be collected at baseline from all patients enrolled into the Phase I safety run in and Phase II reversal of enzalutamide resistance cohort. Where a biopsy is not possible, permission for the biopsy not to take place may be sought from the sponsor (refer to Section 8.2.2 for the minimum number of patients required to undergo biopsies in the Phase II study). Biopsies are **NOT** required from patients enrolled to the Phase II enzalutamide resistance run in.

COVID-19 guidelines: If a patient tests positive for COVID-19 during the screening period, they will not be deemed eligible to proceed with the study. Once they have recovered, tested negative for COVID-19 and are asymptomatic, screening can proceed and the patients' eligibility will be ascertained again at that point.

86 Evaluations during the trial

- **Physical examination**:
  - Phase I safety run in cohort: A complete physical exam will be performed prior to dosing during Cycle 1 and 2 on Day 1, 8, 15, 22, then only on Day 1 of each cycle from Cycle 3 onwards
  - Phase II: A complete physical exam will be performed prior to dosing on Day 1 of every cycle.
- **Vital signs**:
  - Phase I safety run in cohort: Weight, WHO performance status, temperature, supine blood pressure (BP) and pulse rate will be performed prior to dosing during Cycle 1 and Cycle 2 on Day 1, 8, 15, 22, then only on Day 1 of each cycle from Cycle 3 onwards.
  - Phase II: Weight, WHO performance status, temperature, supine blood pressure (BP) and pulse rate will be performed prior to dosing on Day 1 of every cycle.
- **Electrocardiogram (ECG):**
  - Phase I safety run in cohort: Resting 12-lead electrocardiogram (ECG), conducted in triplicate taken up to 5 minutes apart, with manual calculation of QT interval (QTcF) will be performed on Day 1 of each cycle.

  - Phase II: Serial ECGs are not required. Patients will be monitored for symptoms of ischaemic heart disease, which if present, will be investigated with ECGs and additional relevant cardiac investigations.

- **Adverse events and concomitant treatments**: At each visit, before each AZD5069 and/or enzalutamide administration, an assessment of any AE experienced since the previous visit must be made by the Investigator or Research Nurse and the start and stop dates of the AE together with the relationship of the event to treatment with the IMP's must be recorded in the medical records. All AEs must be graded according to NCI CTCAE Version 4.0. Please see section 9.3.1 for exceptions to this. Any concomitant treatment must also be recorded in the medical records and in the eCRF (See Section 9.5 for further details regarding AE reporting requirements).

- **Laboratory tests:** Haematology and biochemistry (as per Section 7.1.3) may be performed 24 hours prior to dosing.
  - Phase I safety run in cohort: Haematology and biochemistry, with the exceptions outlined below, must be repeated weekly during cycle 1 and Cycle 2 on Days1, 8, 15 and 22 and then only on Day 1 of each cycle from Cycle 3 onwards.
  - Phase II: Haematology and biochemistry, with the exceptions outlined below, must be repeated prior to dosing on Day 1 of every cycle.
  - Exceptions:
    - TFH, FSH, glucose, testosterone, and CRP only need to be performed on Cycle Day 1 of every cycle during the Phase I study and on Cycle 1 Day 1 of the Phase II study. HbA1c does not need to be repeated.

- **Urinalysis** – Urine dipstick must be repeated prior to dosing on Day 1 of every cycle.

  Tumour serum markers: PSA must be repeated on the first day of each cycle

- **Radiological disease assessments:** These must be repeated every 3 cycle(s) (Refer to Section 10).

- **Collection of blood samples for PK analysis (Refer to Section 8):**
  - Phase I safety run in cohort: Blood samples for determination of AZD5069 concentrations will be taken on Cycle 1 Day 1 at the following time-points: pre-dose, then post-dose 30 min, 1, 2, 4, 8, 24 and 48 hours. Blood samples for determination of AZD5069 and enzalutamide concentrations will be collected on Cycle 2 Day 1 at the following time-points: pre-dose, then post-dose 30 min, 1, 2, 4, 8, 24 and 48 hours.
  - Phase II: PK samples are not required.

Refer to Section 8 for details of the schedule of PK samples.

- **<u>Collection of blood sample for PD analysis</u>**
    - Phase I safety run in cohort: PD samples will be taken from all evaluable patients in the Phase I safety run in cohort. Blood samples (all pre-dose) will be collected prior to study drug administration on Cycle 1 Day 1, on Cycle 1 Day 15 and on Day 1 of every cycle thereafter for patients treated at all dose levels (Refer to Section 8).
    - Phase II reversal of enzalutamide resistance cohort: Blood samples (all pre-dose) will be collected on Day 1 of each cycle (Refer to Section 8).
    - Phase II enzalutamide resistance run in cohort: PD samples not required.
- **<u>Tumour biopsy</u>**
    - Phase I safety run in cohort: An optional biopsy on Cycle 2 Day 1 (+/- 3 days) will be performed if the patient provides consent.
    - Phase II reversal of enzalutamide resistance cohort: performed on Cycle 2 Day 1 (+/- 3 days). Where a biopsy is not possible, permission for the biopsy not to take place may be sought from the sponsor (refer to Section 8.2.2 for the minimum number of patients required to undergo biopsies).
    - Phase II enzalutamide resistance run in cohort: No Biopsy required.

COVID-19 guidelines: Any deviations from the protocol in scheduled samples due to COVID-19 infection (confirmed by testing) will be discussed and agreed with the Chief Investigator and will be carefully documented for each patient.

87 Evaluations at 'off-study' visit

Evaluations at the 'off-study' visit must be performed within 28 days after the last dose of enzalutamide and AZD5069. The following investigations must be done:

- A symptom-directed physical examination including WHO performance status, temperature, pulse rate, BP (supine) and bodyweight.
- ECG
- Haematology – detailed in Section 7.1.3.
- Biochemistry – detailed in Section 7.1.3, including:
    - T4, TSH, FSH,
    - HbA1c
    - CRP and fasting blood glucose
- Urinalysis detailed in Section 7.1.3
- Prostate-specific antigen (PSA).
- Radiological disease assessments – CT scan of thorax, abdomen and pelvis; or MRI scan of abdomen and pelvis, with CT chest and bone scan (if indicated)

if more than 8 weeks since last scans. In patients with no measurable disease by routine radiological assessment, whole body MRI scan is recommended to determine whether disease is measurable.

- Blood samples for PD analysis (Refer to Section 8).
- Assessment of AEs (Refer to Section 9.0); and
- Assessment of concomitant treatments.

88 Follow-up

Patients will be followed up for 28 days after the last administration of enzalutamide and AZD5069. If any AEs and SAEs are considered to have a highly probable, probable or possible causal relationship to enzalutamide or AZD5069 and are still present 28 days after the last administration of enzalutamide and AZD5069 or occur in the 28 days post enzalutamide and AZD5069 administration; then the patient will be followed up monthly afterwards until resolution, to baseline or stabilisation of these events.

For patients who refuse or are unable to attend further clinic study visits, telephone contact should be attempted to follow up for adverse events 28 days after the last dose of study drug. All serious adverse events will be followed until resolution, until the event has stabilized and/or become chronic, until it has been determined that the event was caused by aetiology other than the study drug.

Patients within the phase II reversal of enzalutamide resistance cohort should be followed up for survival status every 3 months from the safety follow up visit for the first 12 months and then 6 monthly thereafter. If a patient withdraws for any reason prior to radiological progression then the patient should be assessed until radiological progression has occurred or a subsequent line of treatment is started.

Reasonable effort should be made to contact any patient lost to follow-up during the course of the study in order to complete study related assessments and retrieve any outstanding data and study drug. Such efforts should be documented in the source documents.

89 Schedule of events

90 Phase I safety run in cohort

Observation/Investigation	Baseline/Pre-study		Cycle 1				Cycle 2				Cycle 3 onwards		Off Study and Follow Up
	Within 4 Weeks	Within 1 Week	Day 1	Day 8	Day 15	Day 22	Day 1 (+/- 1)	Day 8	Day 15	Day 22	Day 1 (+/-2)	Off Study: ≤ 28 days after last dose of IMP	Follow-up monthly
Written informed consent	X												
Inclusion/Exclusion criteria	X												
Demographics	X												
Medical history	X												
Adverse event evaluation	X	X	X	X	X	X	X	X	X	X	X	X	Until resolution (a)
Concomitant treatments	X	X	X	X	X	X	X	X	X	X	X	X	
Archival tumour sample	X												
Radiological (CT, MRI, bone scan) disease assessment (b)	X										X(b)	X	
Prostate-specific antigen (PSA)		X	X				X				X	X	
Physical examination		X	X	X	X	X	X	X	X	X	X	X	
WHO performance status		X	X	X	X	X	X	X	X	X	X	X	
Vital Signs		X	X	X	X	X	X	X	X	X	X	X	
Electrocardiogram (ECG)	X		X				X				X	X	
Laboratory tests: haematology and biochemistry (c)		X	X	X	X	X	X	X	X	X	X	X	
Urinalysis	X		X				X				X	X	

Observation/Investigation	Baseline/Pre-study		Cycle 1				Cycle 2				Cycle 3 onwards		Off Study and Follow Up
	Within 4 Weeks	Within 1 Week	Day 1	Day 8	Day 15	Day 22	Day 1 (+/- 1)	Day 8	Day 15	Day 22	Day 1 (+/-2)	Off Study: ≤ 28 days after last dose of IMP	Follow-up monthly
Blood for PK analysis (d)			X				X						
Blood for PD analysis (e)		X	X		X		X				X	X	
cfDNA			X				X				X	X	
Tumour biopsy (f)		X (f)					X (f)						
AZD5069 administration			X	X	X	X	X	X	X	X	X		
Enzalutamide administration			X	X	X	X	X	X	X	X	X		

a) Monthly follow-up required ONLY for those AEs and SAEs considered drug-related (highly probable, probable or possible) that is present at time that patient comes off the trial. Monthly follow-up to continue until resolution, to baseline or stabilisation.

b) Radiological disease assessment will be performed after every 3 cycles (12 weeks) of treatment.

c) Haematology and biochemistry to be performed pre-dose (up to 24 hours before), weekly for Cycle 1 and 2, then may be reduced to pre-dose (up to 24 hours before) on Day 1 of every cycle thereafter. TSH, T4, FSH, CRP, HbA1c and fasting blood glucose to be performed at screening (i.e. within 7 days of study commencement) and at the 'off-study' visit. TSH, FSH, glucose, testosterone and CRP will be repeated on Day 1 of every cycle. In the event of a Grade 4 neutropenia or Grade 4 thrombocytopenia a full blood count must be performed at least 7 days after the onset of the event to determine if a dose limiting toxicity has occurred. Continue close monitoring until resolution to Grade 1 or less.

d) PK samples should be taken at the following time points: pre-dose, then post-dose 30 min, 1h, 2h, 4h, 8h and 24h and 48h on Cycle 1 Day 1 and Cycle 2 Day 1 (Refer to 8.3.1)

e) Blood samples for PD biomarker analysis should be taken at the following time-points: pre-dose on Cycle 1 Day 1, pre-dose on Cycle 1 Day 15, pre-dose on Cycle 2 Day 1 and pre-dose on Day 1 of each cycle thereafter and at off study (Refer to Section 8.3.1)

f) Tumour biopsies should be taken from all patients at baseline within one week preceding Cycle 1 Day. Where a biopsy is not possible, permission for the biopsy not to take place may be sought from the sponsor. The biopsy on Cycle 2 Day 1 (+/- 3 days) is optional but preferred.

91 Phase II reversal of enzalutamide resistance cohort

Observation/Investigation	Baseline/Pre-study		Cycle 1	Cycle 2	Cycle 3 onwards	Off Study and Follow Up	
	Within 4 Weeks	Within 1 Week	Day 1	Day 1 (+/- 1)	Day 1 (+/-2)	Off Study: ≤ 28 days after last dose of IMP	Follow-up monthly
Written informed consent	X						
Inclusion/Exclusion criteria	X						
Demographics	X						
Medical history	X						
Adverse event evaluation	X	X	X	X	X	X	Until resolution (a)
Concomitant treatments	X	X	X	X	X	X	
Archival tumour sample	X						
Radiological (CT, MRI, bone scan) disease assessment (b)	X				X(b)	X	
Prostate-specific antigen (PSA)		X	X	X	X	X	
Physical examination		X	X	X	X	X	
WHO performance status		X	X	X	X	X	
Vital Signs		X	X	X	X	X	
Electrocardiogram (ECG)	X					X	
Laboratory tests: haematology and biochemistry (c)		X	X	X	X	X	
Urinalysis	X		X	X	X	X	
Blood for PD analysis		X	X	X	X	X	
Tumour biopsy (d)		X (d)		X (d)			

Observation/Investigation	Baseline/Pre-study		Cycle 1	Cycle 2	Cycle 3 onwards	Off Study and Follow Up	
	Within 4 Weeks	Within 1 Week	Day 1	Day 1 (+/- 1)	Day 1 (+/-2)	Off Study: ≤ 28 days after last dose of IMP	Follow-up monthly
AZD5069 administration			X	X	X		
Enzalutamide administration			X	X	X		
Survival status							X (e)

a) Monthly follow-up required ONLY for those AEs and SAEs considered drug-related (highly probable, probable or possible) that is present at time that patient comes off the trial. Monthly follow-up to continue until resolution, to baseline or stabilisation.

b) Radiological disease assessment will be performed after every 3 cycles (12 weeks) of treatment.

c) TSH, T4, FSH, CRP, HbA1c and fasting blood glucose to be performed at screening (i.e. within 7 days of study commencement) and at the ‘off-study’ visit. TSH, FSH, glucose, testosterone and CRP will be taken on Cycle 1 Day 1. In the event of a Grade 4 neutropenia or Grade 4 thrombocytopenia a full blood count must be performed at least 7 days after the onset of the event to determine if a dose limiting toxicity has occurred. Continue close monitoring until resolution to Grade 1 or less.

d) Tumour biopsies should be taken from all patients within one week preceding Cycle 1 Day 1 and on Cycle 2 Day 1 (+/- 3 days). Where a biopsy is not possible, permission for the biopsy not to take place may be sought from the sponsor (refer to Section 8.2.2 for the minimum number of patients required to undergo biopsies).

e) Patients should be followed up for survival status every 3 months from the off study visit for the first 12 months and then 6-monthly thereafter.

92 Phase II enzalutamide resistance run in cohort

Observation/Investigation	Baseline/Pre-study		Cycle 1	Cycle 2	Cycle 3 onwards	Off Study and Follow Up	
	Within 4 Weeks	Within 1 Week	Day 1	Day 1 (+/- 1)	Day 1 (+/-2)	Off Study: ≤ 28 days after last dose of enzalutamide	Follow-up monthly
Written informed consent	X						
Inclusion/Exclusion criteria	X						
Demographics	X						
Medical history	X						
Adverse event evaluation	X	X	X	X	X	X	Until resolution (a)
Concomitant treatments	X	X	X	X	X	X	
Archival tumour sample	X						
Radiological (CT, MRI, bone scan) disease assessment (b)	X				X(b)	X	
Prostate-specific antigen (PSA)		X	X	X	X	X	
Physical examination		X	X	X	X	X	
WHO performance status		X	X	X	X	X	
Vital Signs		X	X	X	X	X	
Electrocardiogram	X					X	
Laboratory tests: haematology and biochemistry (c)		X	X	X	X	X	
Urinalysis	X		X	X	X	X	
Enzalutamide administration			X	X	X		

a) Monthly follow-up required ONLY for those AEs and SAEs considered drug-related (highly probable, probable or possible) that is present at time that patient comes off the trial. Monthly follow-up to continue until resolution, to baseline or stabilisation.

b) Radiological disease assessment will be performed after every 3 cycles (12 weeks) of treatment.

c) TSH, T4, FSH, CRP, HbA1c and fasting blood glucose to be performed at screening (i.e. within 7 days of study commencement) and at the ‘off-study’ visit. TSH, FSH, glucose, testosterone and CRP will be taken on Cycle 1 Day 1. In the event of a Grade 4 neutropenia or Grade 4 thrombocytopenia a full blood count must be performed at least 7 days after the onset of the event to determine if a dose limiting toxicity has occurred. Continue close monitoring until resolution to Grade 1 or less.

93 Pharmacokinetic and Pharmacodynamic ASSESSMENTS

94 Pharmacokinetic assays

AZD5069 and enzalutamide will be measured in plasma according to agreed SOPs and validated methods. PK evaluation will be undertaken in each patient enrolled in the phase I safety run in cohort.
Blood samples will be collected from all patients enrolled in the Phase I safety run in cohort. 2 mL samples for the determination of AZD5069 concentrations will be collected on Cycle 1 Day 1 at the following time-points: pre-dose then post-dose 30mins, 1, 2, 4, 8, 24 and 48 hours. Two 2ml blood samples for the determination of AZD5069 and enzalutamide concentrations will be collected on Cycle 2 Day 1 at the following time-points: pre-dose then post-dose 30mins, 1, 2, 4, 8, 24 and 48 hours. Please refer to the Study Laboratory Manual for handling, storage and shipment of samples.

95 Pharmacodynamic biomarker assays

96 Archival tumour tissue

Paraffin-embedded tumour tissue must be requested for all patients enrolled into the study. The tumour samples will be in the form of a formalin fixed paraffin embedded (FFPE) block (tissue derived from the diagnostic tumour or a metastatic site). Analysis may be done retrospectively for patients. Please see section 8.2.2 for analysis that will be performed.

Archival tumour biopsies will be analysed at the laboratories of the Institute of Cancer Research. Please refer to the Study Laboratory Manual for handling, storage and shipment of samples.

97 Tumour sampling

All patients in the Phase I study and in the reversal of enzalutamide resistance arm of the Phase II study will be required to provide consent for collection of tumour biopsies. Where a biopsy is not possible, permission for the biopsy(ies) to be omitted may be sought from the sponsor. **A minimum of 10 of 14 patients enrolled in stage 1 of the Phase II reversal of enzalutamide resistance cohort and a minimum of 6 of 11 patients in stage 2 of the Phase II reversal of enzalutamide resistance cohort need to undergo serial biopsies as per the study schedule.**
Tumour biopsies may be collected from either bone or soft tissue disease.
Core needle tumour biopsies will be taken according to local procedures then fixed in formalin and embedded as instructed in the lab manual.
Please refer to the Study Laboratory Manual for handling, storage and shipment of samples.
Tumour biopsies will be analysed at the laboratories of the Institute of Cancer Research to determine the infiltration of tumour by MDSC and T-cells by IF and/or IHC. Molecular characterisation including markers such as PTEN, CXCL-1, CXCL-2, CXCL-5, IL-6, IL-8 and Ki67 will be determined by IF and/or IHC. Pre-clinical data

suggest that MDSCs are recruited into tumours across a chemokine gradient (CXCL1 and CXCL2) through CXCR2 signalling (14). We therefore propose that CXCR2 inhibition by AZD5069 will reduce MDSC infiltration. Fresh tissue biopsies will be taken pre and post four weeks of treatment with AZD5069 in combination with enzalutamide to determine the level of MDSC infiltration. MDSC will be determined by IHC and 5-colour IF with known markers of MDSCs (CD11b+ CD14- CD33+ CD15+) (12). Other assays including NGS and the study of tumour cell senescence will also be pursued. Downstream effect of the inhibition of MDSC trafficking on JAK-STAT-ROR□-AR signalling and AR splice variant expression will also be examined.

98 Blood sampling

PD blood samples will be collected from all patients enrolled into the study as follows:

Phase I safety run in cohort:

- Baseline
- Cycle 1 Day 1 (predose)
- Cycle 1 Day 15 (pre-dose)
- Cycle 2 Day 1 (pre-dose)
- Cycle 3 onwards Day 1 (pre-dose) of each cycle
- Off Study: ≤ 28 days after last dose of IMP, on progression

Phase II reversal of enzalutamide resistance cohort:

- Baseline
- Cycle 1 Day 1 (pre-dose)
- Cycle 2 onwards Day 1 (pre-dose)
- Off Study: ≤ 28 days after last dose of IMP, on progression

Different PD markers will be collected at different time points (Section 8.3.1). PD samples will be analysed at the laboratories of the Institute of Cancer Research. Please refer to the Study Laboratory Manual for handling, storage and shipment of samples.

The planned assays will be prioritised according to an agreed order if limited sample material is available. The proposed assays are likely to change during the study to ensure the most relevant use of the collected samples.

99 Circulating MDSCs

Increased circulating MDSC are also found in patients with prostate cancer, resulting in an elevated NLR (13, 15). High circulating MDSC counts are associated with more advanced disease and worse survival from CRPC (13, 15). Blood samples (3 mL) will be drawn at specified time points (section 8.2.3 above) and immunophenotyping will be performed using fluorescence activated cell sorting, to determine the level of circulating MDSCs and the effects of AZD5069 on circulating MDSC levels.

100 Circulating chemokines and cytokines

Prostate cancer patients also have increased circulating IL-6 and IL-8, which correlate with increased circulating MDSCs (15). Blood (5 mL) will be drawn at specified time points (section 8.2.3 above) and enzyme-linked immunosorbent assay will be performed to determine the levels of cytokines such as IL-6, IL-8, CXCL-1, CXCL-2, CXCL-5 and GM-CSF in whole blood.

101 Neutrophil to lymphocyte ratio

A high neutrophil to lymphocyte ratio (NLR) is associated with poor overall survival from solid tumours (9). Interestingly in mCRPC patients a high NLR was not only associated with poorer prognosis, it was also associated with a significantly poorer response rate to abiraterone and to cabazitaxel (10, 11). Overall, 49% percent of patients with an NLR ≤ 5 responded to abiraterone (PSA decline ≥50% below baseline maintained for ≥3 weeks) compared to 16% of patients with an NLR > 5 (p=0.01) with these data being reproduced in independent test and validation sets from the Princess Margaret Hospital and the Royal Marsden (10). Blood (5 mL) will be drawn at specified time points (section 8.2.3 above) and NLR calculated to determine the effects of AZD5069 on NLR.

102 Circulating tumour cells (CTCs)

The use of CTCs as a potential surrogate biomarker of treatment efficacy has been examined in mCRPC (19). CTC conversion rate is defined as a change from prognostically unfavorable CTC count (≥5 CTC/7.5mls blood) at baseline to a favorable CTC count on therapy (<5 CTC/7.5mls blood). Blood (2 x 10 mL) will be drawn at specified time points (section 8.2.3 above) and CTC will be calculated to determine the effects of AZD5069 on CTC numbers.

103 PAXgene mRNA

Whole blood mRNA expression profiles have been shown to identify gene-expression signatures that stratify patients with metastatic castration resistant prostate cancer into distinct prognostic groups (20). Blood samples (2.5 mL) will be drawn at specified time-points (section 8.3.1) and whole blood mRNA expression profiles will be determined to identify whether patients with high numbers of MDSCs within their tumour and circulating blood have specific profiles.

104 Cell-free DNA (cfDNA)

cfDNA, nucleic acid released by tumour cells into the blood stream, can be detected in the serum or plasma of patients with cancer to monitor disease burden, response to treatment, and to identify potential mechanisms of resistance. Blood samples (10 ml x 2) will be drawn at specified time-points (section 7.5 and section 8.3) and stored and tested retrospectively for level of cfDNA and the frequency of specific mutant alleles if deemed of clinical interest.

105 Schedule of research samples

106 Phase I safety run in cohort

Time points	Tissue			Blood based biomarkers					
	Archival	Fresh	CTC	EDTA: Circulating MDSC	PAXgene mRNA	Circulating chemokines	Streck: cfDNA	PK – AZD5069	PK – Enzalutamide
Screening	X	X (a)	2x10ml	1x3ml	1x2.5ml	1x5ml	2x10ml		
C1D1 (pre-dose)			2x10ml	1x3ml	1x2.5ml	1x5ml	2x10ml	X	
C1D1 (post-dose)								30min, 1, 2, 4, 8h	
C1D2								X (24h)	
C1D3								X (48h	
C1D15 (pre-dose)			2x10ml	1x3ml	1x2.5ml	1x5ml	2x10ml		
C2D1 (pre-dose)		X(b)	2x10ml	1x3ml	1x2.5ml	1x5ml	2x10ml	X	X
C2D1 (post-dose)								30min, 1, 2, 4, 8h	30min, 1, 2, 4, 8h
C2D2								X (24h)	X (24h)
C2D3								X (48h)	X (48h)
C3D1 (pre dose) and each subsequent			2x10ml	1x3ml	1x2.5ml	1x5ml	2x10ml		
Treatment discontinuation			2x10ml	1x3ml	1x2.5ml	1x5ml	2x10ml		

(a) Tumour biopsies should be taken from all patients at baseline within one week preceding Cycle 1 Day 1. Where a biopsy is not possible, permission for the biopsy not to take place may be sought from the sponsor.
(b) A further biopsy on Cycle 2 Day 1 is optional but preferred.

Phase II reversal of enzalutamide resistance cohort

Time points	Tissue			Blood based biomarkers			
	Archival	Fresh	CTC	EDTA: Circulating MDSC	PAXgene mRNA	Circulating chemokines	Streck: CfDNA
Screening	X	X(a)	2x10ml	1x3ml	1x2.5ml	1x5ml	2x10ml
C1D1 (pre-dose)			2x10ml	1x3ml	1x2.5ml	1x5ml	2x10ml
C2D1 (pre-dose)		X(a)	2x10ml	1x3ml	1x2.5ml	1x5ml	2x10ml
C3D1 (pre-dose) and each subsequent			2x10ml	1x3ml	1x2.5ml	1x5ml	2x10ml
Treatment discontinuation			2x10ml	1x3ml	1x2.5ml	1x5ml	2x10ml

(a) Where a biopsy is not possible, permission for the baseline and Cycle 2 Day 1 biopsies to be omitted may be sought from the sponsor.

107 ASSESSMENT OF SAFETY

108 Adverse event definitions

109 Adverse event

For eligible patients, adverse event (AE) and serious adverse events (SAE) collection and monitoring commences from the time the patient gives their written consent to participate in the trial and continues for 28 days after the last administration of either enzalutamide or AZD5069 (investigational medicinal product; IMP).

Follow-up of AEs with a causality of possible, probable or highly probable will continue until the events resolve or stabilise.

An AE is any untoward, undesired or unplanned occurrence in a patient administered an IMP. An AE can be a sign, symptom, disease, and/or clinically significant laboratory or physiological observation that may or may not be related to the IMP. All AEs will be recorded and graded according to CTCAE Version 4.0 terminology. Please see section 9.3.1 for exceptions to this.

An AE includes but is not limited to those in the following list:

- A clinically significant worsening of a pre-existing condition. This includes conditions that may resolve completely and then become abnormal again.
- AEs occurring from an overdose of an IMP, whether accidental or intentional.

Other reportable events that must be treated as AEs are listed below.

- Pregnancy exposure to the IMP. Any pregnancy occurring in a patient or a patient's partner during treatment with an IMP or occurring within six months of the last IMP administration, must be reported to the PV data manager in the same timelines as an SAE. These should be reported even if the patient is withdrawn from the trial.
- Any AE associated with an overdose or incorrect administration of IMP. An overdose or incorrect administration of IMP is not an AE unless it results in untoward medical effects.

- Any AE that could be related to the protocol procedures, and which could modify the conduct of the trial.

110 Serious adverse events (SAEs)

An SAE is any AE, regardless of dose, causality or expectedness, that:

- Results in death.
- Is life-threatening.
- Requires in-patient hospitalisation or prolongs existing in-patient hospitalisation (some hospitalisations are exempt from SAE reporting – see Section 9.2.1).
- Results in persistent or significant incapacity or disability.
- Is a congenital anomaly or birth defect.
- Is any other medically important event*

*A medically important event is defined as any event that may jeopardise the patient or may require intervention to prevent an SAE. It should be noted that medically important events might be identified before or at any point in the study.

111 Determining adverse event causality

The relationship of an AE to the IMP is determined as follows.

**Highly probable**<br>• Starts within a time related to the IMP administration and<br>• No obvious alternative medical explanation.
**Probable**<br>• Starts within a time related to the IMP administration and<br>• Cannot be reasonably explained by known characteristics of the patient's clinical state.
**Possible**<br>• Starts within a time related to the IMP administration and<br>• A causal relationship between the IMP and the AE is at least a reasonable possibility.
**Unlikely**<br>• The time association or the patient's clinical state is such that the trial drug is not likely to have had an association with the observed effect.
**Not related**<br>• The AE is definitely not associated with the IMP administered.

Note: Drug-related refers to events assessed as possible, probable or highly probable.

The Investigator must endeavour to obtain sufficient information to determine the causality of the AE (i.e. IMP, other illness, progressive malignancy etc) and must provide his/her opinion of the causal relationship between each AE and IMP. This may require instituting supplementary investigations of significant AEs based on their clinical judgement of the likely causative factors and/or include seeking a further opinion from a specialist in the field of the AE.

112 Expectedness

Assessment of expectedness will be made by the Chief Investigator (or delegate) against the current version of the following (depending on batch type):

- SmPC (section 4.8) for enzalutamide capsules/tablets provided in blister packs
- U.S enzalutamide Full Prescribing Information (Adverse Reaction section) for enzalutamide capsules provided in bottles
- AZD5069 IB (reference safety information section).

113 Suspected, unexpected, serious, adverse reactions (SUSARs)

A SUSAR is a suspected, unexpected, serious adverse reaction. All AEs and SAEs will be assessed for seriousness, causality and expectedness. The DDU Pharmacovigilance Officer will expedite all SUSARs to the relevant Competent Authority/Authorities and the relevant Ethics Committee(s) within the timelines specified in legislation (SI 2004/1031 as amended i.e. 15 days or 7 days for fatal/life threatening events.

114 Expedited reporting of serious adverse events to the Pharmacovigilance Officer, DDU

All SAEs, regardless of causality, must be reported to the DDU Pharmacovigilance Officer in an expedited manner.

SAEs should be documented on an SAE report form, using the completion guidelines provided.

The SAE report form must be emailed to the DDU Pharmacovigilance Officer within 24 hours of site staff becoming aware of the SAE.

Email address: ddu-sae@icr.ac.uk

Each episode of an SAE must be recorded on a separate SAE report form. The NCI CTCAE Version 4.0 must be used to grade each SAE, and the worst grade recorded.

If new or amended information on a previously reported SAE becomes available, the Investigator should report this to the DDU Pharmacovigilance Officer on a new SAE report form.

Should the Investigator become aware of any drug-related SAEs after the patient goes ‘off study’, these must also be reported to the DDU Pharmacovigilance Officer within the specified timelines above.

115 Events exempt from being reported as SAEs

Events specified in this section do not require reporting as SAEs in this trial, unless hospitalisation is prolonged for any reason and then an SAE form must be completed. The events must still be recorded in the appropriate section of the case report form (CRF).

Screening period – SAEs occurring in the period prior to administration of enzalutamide and AZD5069 from the time of informed consent the commencement of any study drug that are not related to the design or conduct of the trial are exempt from reporting.

Elective admissions – Elective admissions to hospital for procedures which were planned and documented in the medical records at the time of consent are not SAEs, and do not require SAE reporting. Hospitalisation for administration of the IMP according to the trial protocol is also exempt from being reported as an SAE.

If per protocol a visit is expected to be on-site but the patient was contacted by phone, the data should be entered using the regular eCRF pages for that visit to capture all data and all of the assessments completed remotely (e.g. adverse events, and any additional safety information that can be obtained remotely).

116 Recording of adverse events and serious adverse events in eCRFs

All AEs, including SAEs, must be recorded in the eCRF for eligible patients. All concomitant medications, including herbal medications and supplements must be recorded. Any therapy used to treat the event must be recorded. The eCRF will be reconciled with the safety database during and at the end of the trial. Therefore, the sites should ensure the data entered on the SAE report form and the data entered into the eCRF are consistent. The Safety Review Committee and the Investigator(s) will regularly review the safety data from both the safety and the clinical database.

If per protocol a visit is expected to be on-site but the patient was contacted by phone, the data should be entered using the regular eCRF pages for that visit to capture all data and all of the assessments completed remotely (e.g. adverse events, and any additional safety information that can be obtained remotely).

117 Recording of anaemia and hypokalaemia

The NCI CTCAE Version 4.0 must be used to grade all AEs except Grade 2 or Grade 3 anaemia and Grade 1 or Grade 2 hypokalaemia. This is a clinical decision made by the CI due to historical inconsistencies with grading of hypokalaemia and anaemia.

Grade 2 or Grade 3 anaemia should be recorded by the lab value alone, regardless of any transfusion given. If a transfusion is given prophylactically or as a supportive care for a Grade 2 anaemia, this will be recorded in the patients notes and in the eCRF concomitant medication page; i.e. transfusion (prophylaxis).

Grade 1 or Grade 2 for hypokalaemia with any intervention will remain as Grade 1, where intervention is long-term supplements or oral medication. Exception to this rule is if intravenous intervention is required or if patient is symptomatic and deemed Grade 2.

CTCAE Version 4.0 should be used to treat all other grades of anaemia and hypokalaemia.

118 Follow-up of adverse events

Follow-up will continue until all the necessary safety data for the event has been gathered and until the drug-related AE or SAE has either resolved, returned to baseline or stabilised. The DDU data manager (PV) will make requests for further information to the trial site at regular intervals. Requested follow-up information should be reported to the DDU Pharmacovigilance Officer in a timely manner and as soon as possible after receipt of the follow-up request. For fatal or life-threatening cases, follow-up information should be reported as soon as possible.

119 Urgent safety measures

The Sponsor or Investigator may take appropriate urgent safety measures (USMs) in order to protect the patient of a clinical trial against any immediate hazard to their health or safety. This includes procedures taken to protect patients from pandemics or infections that pose serious risk to human health.
USMs may be taken without prior authorisation from the competent authority.
The Medicines and Healthcare products Regulations Agency (MHRA) and the Main Research Ethics Committee (REC) must be notified within three days of such measures being taken.
Should the site initiate a USM, the Investigator must inform the Sponsor immediately either by:

- Email: ace@icr.ac.uk; or
- Telephone: 020 8661 3752; or
- Fax: 020 8915 6076.

The notification must include:

- The date of the USM;
- Who took the decision; and
- Why action was taken.

The Sponsor will then notify the MHRA and the Main REC within three days of USM initiation.

120 Pregnancy

The Investigator must make every effort to try and ensure that a partner of a clinical trial patient does not become pregnant during the trial or for six months afterwards. This should be done as part of the consent process by explaining clearly to the patient the potential dangers of becoming pregnant and also providing each patient with information about appropriate medically approved contraception. Two forms of medically approved contraception should be used, such as:

- oral contraceptives and condom;
- intra-uterine device (IUD) and condom;
- diaphragms with spermicidal gel and condom.

Contraceptives should be used from the time the patient joins the trial, throughout the trial and for six months after completing the trial.
It should be explained to the patient that if his partner is pregnant or breast-feeding when he enters the trial, the patient should use barrier method contraception (condom plus spermicidal gel) to prevent the unborn baby or the baby being exposed to the enzalutamide and AZD5069.
However, if a partner of a patient does become pregnant, the reporting procedures below must be followed.
Any pregnancy occurring in the patient's partner during treatment with an IMP or occurring within six months of last IMP administration must be reported to the DDU Pharmacovigilance Officer within 24 hours of the site staff becoming aware of it using a Pregnancy Notification Form. It is the Investigator's responsibility to obtain consent for follow-up from the patient's partner. The DDU Pharmacovigilance Officer will follow-up all pregnancies for the pregnancy outcome via the Investigator and document on the pregnancy form.
The Investigator must ensure that all patients are aware at the start of a clinical trial of the importance of reporting all pregnancies (in their partners) that occur whilst being treated with the IMP and occurring up to six months after the last IMP administration. The Investigator should offer counselling to the patient and/or the partner, and discuss the risks of continuing with the pregnancy and the possible effects on the foetus. Monitoring of the patient's partner and the baby should continue until the conclusion of the pregnancy, if the patient's partner has consented to this.

121 ASSESSMENT OF EFFICACY

122 Measurement of disease

Antitumour activity will be defined by response rate on the basis of the following outcomes; if any of these occur, patients will be considered to have responded:

- PSA decline ≥ 50% criteria confirmed 4 weeks or later and/or,
- Confirmed soft tissue objective response by RECIST (v1.1) in patients with measurable disease and/or,
- ONLY for patients with detectable circulating tumour cell count (CTC) of ≥ 5/7.5ml blood at baseline, conversion of CTC <5/7.5ml blood nadir.

123 Timing and type of tumour assessments

A thorough clinical and radiological evaluation of malignancy, as judged appropriate by the Investigator, and in line with the protocol, must be performed before starting the IMP. The same methods that detect evaluable lesions at baseline must be used to follow these lesions throughout the trial. To ensure compatibility, the radiological

assessments used to assess response must be performed using identical techniques. Imaging based evaluation is preferred to evaluation by clinical examination when both methods have been used to assess the anti-tumour effect of a treatment.
All radiological assessments must be performed within four weeks before starting treatment. The interval between the last anti-cancer therapy (excluding enzalutamide) and these measurements must be at least four weeks (28 days). All clinical measurements to assess response must be done within one week before the patient starting treatment. All complete (CR) and partial responses (PR) must be confirmed by two consecutive observations not less than four weeks apart (in addition to planned evaluations at 12 weekly intervals).

124 Baseline evaluations

These must include radiological measurements and as indicated chest, abdominal and pelvic CT scan; or MRI scan of abdomen and pelvis with chest CT scan, bone scan and PSA evaluation. In patients with no measurable disease by routine radiological assessment, whole body MRI scan is recommended to determine whether disease is measurable. All areas of disease present must be documented (even if specific lesions are not going to be followed for response) and the measurements of all measurable lesions must be recorded clearly on the scan reports. Any non-measurable lesions must be stated as being present.

125 Evaluations during and at 'off-study'

Tumour assessments must be repeated every 3 cycles (12 weeks) if continuous treatment or more frequently, when clinically indicated. All lesions measured at baseline must be measured at every subsequent disease assessment, and recorded clearly on the scan reports. All non-measurable lesions noted at baseline must be noted on the scan report as present or absent. All patients, who are removed from the trial for reasons other than progressive disease, must be re-evaluated at the time of treatment discontinuation, unless a tumour assessment was performed within the previous four weeks. It is the responsibility of the Principal Investigator to ensure that the radiologists are aware of the requirement to follow-up and measure every target lesion mentioned at baseline and comment on the non-target lesions in accordance with RECIST (v1.1) criteria (appendix 3).

126 Tumour response

All patients who meet the eligibility criteria, receive at least three cycles (12 weeks) of trial medication and have a baseline assessment of disease will be evaluable for response (section 10.1). **If any of these criteria are met, patients will be considered to have responded.** For disease progression; RECIST (v1.1) and prostate cancer working group 2 (PCWG2) criteria will be used (section 3.6). **If any of these criteria are met, patients will be considered to have progressed.**

127 Recording of response in the eCRF

The applicable overall response category for each visit that includes disease assessment must be recorded in the eCRF, even though the criteria for determination of CR or PR by the protocol must be confirmed after two consecutive observations, no less than four weeks apart.

128 Discontinuation of treatment/ withdrawal from trial

The Investigator must make every reasonable effort to keep each patient on trial for the whole duration of the trial (i.e. until 28 days after last combination therapy administration). However, if the Investigator removes a patient from the trial or if the patient declines further participation, final 'off-study' assessments should be performed before any therapeutic intervention. All the results of the evaluations and observations, together with a description of the reasons for withdrawal from the trial, must be recorded in the medical records and in the eCRF.

Patients who are removed from the trial due to adverse events (clinical or laboratory) will be treated and followed according to accepted medical practice. All pertinent information concerning the outcome of such treatment must be recorded in the eCRF and on the serious adverse event (SAE) report form where necessary.

The following are justifiable reasons for the Investigator to withdraw a patient from trial.

- Unacceptable toxicity
- AE/SAE
- Withdrawal of consent
- Serious violation of the trial protocol (including persistent patient attendance failure and persistent non-compliance)
- Sponsor's decision to terminate the trial
- Withdrawal by the Investigator for clinical reasons not related to the IMP
- Evidence of disease progression
- Symptomatic deterioration

129 DEFINING THE End of triaL

The 'end of trial' is defined as the date when the last patient has completed the 'off-study' visit or the final follow-up visit (whichever is the latter).

It is the responsibility of the DDU to inform the Medicines and Healthcare products Regulations Agency (MHRA) and the Main Research Ethics Committee (REC) within 90 days of the 'end of the trial' that the trial has closed.

In cases of early termination of the trial (for example, due to toxicity) or a temporary halt by the DDU, the DDU will notify the MHRA and the Main REC within 15 days of the decision and a detailed, written explanation for the termination/halt will be given.

The entire trial will be stopped when:

- The drug is considered too toxic to continue treatment before the required number of patients being recruited.

- The stated number of patients to be recruited is reached.
- The stated objectives of the trial are achieved.

Regardless of the reason for termination, all data available for the patient at the time of discontinuation of follow-up must be recorded in the eCRF. All reasons for discontinuation of treatment must be documented.
In terminating the trial, DDU and the Investigators must ensure that adequate consideration is given to the protection of the patient's interest.

130 DATA ANALYSIS and statistical considerations

131 Statistical design

Documenting anti-tumour activity is a primary objective for the Phase II reversal of enzalutamide resistance cohort(s). Two RP2Ds may be taken forward to the phase II study, with up to 25 patients in each of the Phase II reversal of enzalutamide resistance cohorts (up to 50 patients if two dose levels are taken forward to the phase II study). Each cohort will pursue a two-stage Simon design to test the null hypothesis that P ≤ 0.05 (undesirable response rate) versus the alternative that P ≥ 0.25 (desirable response rate). In each reversal of enzalutamide resistance cohort, stage 1 will recruit 14 patients. If at least 1 response is observed, recruitment will continue to a total of 25 patients. Data from all patients recruited to this cohort will be used to estimate the response rate to treatment. If the total number responding is less than or equal to 3, the RP2D will not be considered for further evaluation; if there are at least 4 responses in 25 patients, the RP2D is regarded an effective dose. This design has an expected sample size of 19.64 and a probability of early termination of 0.488. If the RP2D is actually not effective, there is a 0.033 probability of concluding that it is (type I error). If the RP2D is actually effective, there is a 0.101 probability of concluding that it is not (type II error). After the 14th patient has completed stage 1 in a reversal of enzalutamide resistance cohort, the IDMC will review the response data for the first 14 patients and advise on the continuation to stage 2. Recruitment may continue whilst the interim analysis of the primary outcome is being conducted in preparation for the IDMC. If two RP2Ds are taken forward to the phase II study, review of the response data and the decision for the cohort to progress from stage 1 to stage 2 will occur independent of the other cohort being evaluated. At the time of adding dose level 5 (+/- 4b) to the phase 1 study, dose level 4 was already been established as safe and tolerable and phase II expansion at this dose (160 mg BD) had begun Therefore, if another RP2D is established, there is likely to be no or little overlap in the recruitment phases of the two phase II expansions. If dose expansion is to occur at a second RP2D, and there are less than 8 remaining patients to be allocated to the expansion at dose level 4, patients will be systematically allocated to level 4 before expanding at a higher dose level; if 8 or more remaining patients are to be allocated to the expansion at dose level 4 when a second expansion cohort is to begin, then patients will be randomly allocated to the two arms.

If two dose levels are expanded in the phase II study, the dose level that will proceed to the next phase of evaluation will be determined based on antitumour activity, defined by response rate and duration of response, provided the safety profile is deemed acceptable by the IDMC. The group with a higher rate of response, with a minimum of 4% improvement in response rate compared to the other dose group (i.e., at least one response more in 25 patients), will be taken forward. If response rates are equivalent in both groups, the group with a longer average duration of response will be taken forward. The operating characteristics of the proposed selection design will be provided in the Statistical Analysis Plan.

132 Analysis Populations

Intention to treat (ITT): This population includes all patients enrolled into the study regardless of whether they are later found to be ineligible, a protocol violator, never treated or not evaluated. Patients for whom the primary endpoint cannot be evaluated will be treated as non-responders.
Evaluable-patient population: This population contains all enrolled patients for whom the primary endpoint can be evaluated. Where evaluability is difficult to define the final decision will rest with the IDMC.

- For phase I safety run in cohort: Patients who complete the DLT period or experience a DLT during the DLT period are considered evaluable.
- For the phase II reversal of enzalutamide resistance cohort: Patients who meet all of the relevant inclusion and exclusion criteria and who start trial treatment are considered evaluable, unless they discontinue prior to 12 weeks for reasons which aren’t drug or disease related.

Safety population: This population includes all enrolled patients who received at least 1 treatment dose of either of the treatment drugs.
Analysis of the primary outcome will use the evaluable-patient population with sensitivity analyses in the ITT population. All other analyses will use the ITT population. The safety population will be used to characterise the safety and tolerability profile of the treatment regimens in the phase I safety run-in and phase II reversal of enzalutamide resistance cohort.

133 Presentation of data

Data will be presented in a descriptive fashion. Variables will be analysed to determine whether the criteria for the trial conduct are met. This will include a description of patients who did not meet all the eligibility criteria, an assessment of protocol violations, IMP accountability and other data that impact on the general conduct of the trial. Baseline characteristics will be summarised for all enrolled patients. Patients who died or withdrew before treatment started or did not complete the required safety observations will be described and evaluated separately. Treatment administration will be described for all cycles. Dose administration, dose modifications or delays and the duration of therapy will be described.

134 Safety

Safety data will be collected from the date of written consent. Safety variables will be summarised by descriptive statistics. Laboratory variables will be described using the National Cancer Institute (NCI) Common Terminology Criteria for Adverse Events (CTCAE) Version 4.0. Please see section 9.3.1 for exceptions to this.
Adverse events (AEs) will be reported for each dose level and presented as tables of frequency of AEs by body system and by worse severity grade observed. Tables should indicate related and unrelated events. Laboratory data will be presented by dose level at each observation time. Values outside normal limits will be identified and summarised by frequency distribution. All enrolled patients that received at least 1 dose of AZD5069 will be included in the safety analysis

135 Pharmacokinetics

The plasma concentration/time data will be analysed using non-compartmental methods. The pharmacokinetic (PK) parameters to be determined for AZD5069 and enzalutamide include maximum plasma concentration (C_{max}), time to reach C_{max} (T_{max}), the area under the plasma concentration time curve (AUC), the terminal elimination half-life ($T_{1/2}$), mean residence time (MRT), **apparent** total body clearance (**CL/F**) and **apparent** steady/state volume of distribution (**$V_{SS/F}$**).

136 Pharmacodynamics

Blood and tumour samples from pre and post-treatment will be analysed for changes in NLR, circulating and intratumoral myeloid inflammatory cell (e.g. MDSC) count, transcriptome expression and circulating chemokine and cytokine levels; data will be presented primarily using descriptive statistics. Additional parameters may be evaluated based on emerging data.
Where a biopsy is not possible, permission for the biopsy(ies) to be omitted may be sought from the sponsor. A minimum of 10 of 14 patients enrolled in stage 1 of the Phase II reversal of enzalutamide resistance cohort and a minimum of 6 of 11 patients in stage 2 of the Phase II reversal of enzalutamide resistance cohort need to undergo serial biopsies as per the study schedule.

137 Anti-tumour activity

138 Phase I safety run in cohort

Documenting anti-tumour activity is a secondary objective for the safety run in phase and will be described by PSA, radiological criteria and CTC evaluation (section 10.1). Patients must receive 12 weeks of trial treatment to be evaluable for response. The number of patients achieving a response to treatment will be presented along with a 95% confidence interval. Analysis will be carried out when all patients have completed 12 weeks of combination treatment or discontinued prior to this.

139 Phase II reversal of enzalutamide resistance cohort

If two RP2Ds are taken forward to the phase II study in two separate reversal of enzalutamide resistance cohorts, the IDMC will review the response data in each cohort to make a decision for the cohort to progress from stage 1 to stage 2 independent of the other cohort being evaluated. Analysis of the first stage of a reversal of enzalutamide resistance cohort will be triggered once 14 patients have completed 12 weeks of combination treatment or have discontinued prior to this. Recruitment may continue whilst the interim analysis of the primary outcome is being conducted in preparation for the IDMC. If 1 or more response is observed recruitment will continue up to a total of 25 patients. This will be assessed by the IDMC. The primary analysis will be triggered when all patients have completed 12 weeks of combination treatment or discontinued prior to this. The last value of PSA, CTC count (if available), CT and bone scan up to 4 weeks before the C1D1 will be used as baseline value for the response assessment. The response rate will be presented along with the corresponding 95% confidence interval. The best overall response will also be presented.

Kaplan Meier curves will be used to summarize the time to event endpoints (OS and rPFS). The median rPFS and OS estimates will be reported along with 95% CIs. Response criteria will each be presented separately as waterfall plots for PSA (maximum PSA decline and PSA change), CTC (% fall) and RECIST measurement changes. Baseline and nadir CTC counts will be presented below the waterfall plot. The proportion of patients with at least a 30% fall in CTC will be reported along with its 95% CI. The number of skeletal related events will be presented along with the 95% CI.

If two dose levels are expanded in the phase II study, the dose level that will proceed to the next phase of evaluation will be determined based on antitumour activity, defined by response rate and duration of response, provided the safety profile is deemed acceptable by the IDMC.

140 ADMINISTRATION

This trial is conducted under a clinical trial authorisation (CTA) and approval from the Health Research Authority (HRA), Medicines and Healthcare products Regulations Agency (MHRA) and the relevant Research Ethics Committee(s) will be obtained before the start of this trial. This trial is sponsored by The Institute of Cancer Research (ICR). Applicable regulatory requirements are described in this section.

141 Protocol deviations and amendments

Do not deviate from the protocol unless approval has been obtained from the DDU. Eligibility waivers are strictly prohibited.

Amendments to the protocol may only be made with the approval of the DDU, CI and statistician. A protocol amendment will be subject to review by the sponsor. Ethics Committee favourable opinion (and MHRA approval if appropriate) will be obtained by the DDU on behalf of the CI before the amendment can be implemented and incorporated into the protocol if necessary.

142 Completion of the case report form (CRF)

Electronic CRFs (eCRFs) will be used to collect the data. The Investigator is responsible for ensuring the accuracy, completeness, clarity and timeliness of the data reported in the eCRFs.
Only the Investigator and those personnel who have signed the Study Team Responsibilities Signature Log/Delegation Log provided by the DDU and have been authorised by the Investigator should enter or change data in the eCRFs. All protocol required investigations must be reported in the eCRF. The Investigators must retain all original reports, traces and images from these investigations for future reference. Data will be entered directly into eCRFs by authorised site personnel.
Once data has been entered by the site personnel on the eCRF, the data will be reviewed and checked for error and inconsistencies by data management staff in the DDU.
Once the patient is 'off study' and the eCRF has been fully completed, the Investigator must provide a signature to authorise the complete subject casebook.

143 Trial performance and monitoring

144 Management of the study

The DDU Clinical Trials Manager will be responsible for the day to day coordination and management of the trial. The ICR Clinical Trials and Statistics Unit (ICR-CTSU) will act as custodian of the data on behalf of the sponsor. The DDU is responsible for all duties relating to pharmacovigilance in accordance with section 9.0.
Before the trial can be initiated, the prerequisites for conducting the trial must be clarified and the organisational preparations made with the trial centre. The DDU must be informed immediately of any change in the personnel involved in the conduct of the trial.
During the trial the DDU Clinical Trials Manager is responsible for monitoring data quality in accordance with DDU's standard operating procedures (SOPs). Before the trial start, the Investigator will be advised of the anticipated frequency of the monitoring visits. The Investigator will receive reasonable notification before each monitoring visit.
It is the responsibility of the Clinical Trials Manager to:

- review trial records and compare them with source documents;
- check pharmacodynamic samples and storage;
- discuss the conduct of the trial and the emerging problems with the Investigator;
- check that the drug storage, dispensing and retrieval are reliable and appropriate; and
- verify that the available facilities remain acceptable.

All unused drug supplied must be returned to the supplier, or if authorised by DDU properly destroyed at the Investigator site by an authorised person who will provide signed confirmation.
It is the responsibility of the DDU to inform the Main REC within 90 days of the 'end of the trial' (i.e. last patient study visit) that the trial has closed.

145 Source document verification

All data collected in the eCRF must be verifiable by the source data. Therefore, it is the Investigator's responsibility to ensure that both he/she and his/her study team records all relevant data in the medical records. The Investigator must allow the CTM direct access to relevant source documentation for verification of data entered into the eCRF, taking into account data protection regulations. Entries in the eCRF will be compared with patients' medical records and the verification will be documented on the source data verification (SDV) form and the monitoring report.
The patients' medical records, and other relevant data, may also be reviewed by appropriate qualified personnel independent from the DDU appointed to audit the trial, and by regulatory authorities. Details will remain confidential and patients' names will not be recorded outside the hospital.

146 Clinical study report

All clinical data will be presented at the end of the trial on final data listings. The CI, DDU and ICR-CTSU will prepare a clinical study report based on the final data listings. The report will be submitted to the Investigator(s) for review and confirmation it accurately represents the data collected during the course of the trial. A summary of the final clinical report will be provided by the DDU to the MHRA and to the Research Ethics Committee.

147 Record retention

During the clinical trial and after trial closure the Investigator must maintain adequate and accurate records to enable both the conduct of a clinical trial and the quality of the data produced to be evaluated and verified. These essential documents (as detailed in Chapter V of Volume 10 (Clinical Trials) of The Rules Governing Medicinal Products in the European Union based upon Section 8 of the ICH GCP Guidelines), including source documents such as scans, trial related documents and copies of the eCRFs, associated audit trail and serious adverse event (SAE) report forms, shall show whether the Investigator has complied with the principles and guidelines of Good Clinical Practice (GCP).
All essential documents required to be held by the Investigator must be stored in such a way that ensures that they are readily available, upon request, to the Regulatory Agency or Sponsor, for the minimum period required by national legislation. Records must not be destroyed without prior written approval from the DDU.
The medical files of trial subjects shall be retained in accordance with national legislation and in accordance with the maximum period of time permitted by the hospital, institution or private practice, Study documentation and medical records must be held for a minimum of 5 years after study conclusion.

148 Ethical considerations

Before starting the trial, the protocol, patient information sheet and consent form must be approved by the ICR/RM joint Committee for Clinical Research and the appropriate Ethics Committee.
It is the Chief/Principal Investigator's responsibility to update patients (or their authorised representatives, if applicable) whenever new information (in nature or severity) becomes available that might affect the patient's willingness to continue in the trial. The Chief/Principal Investigator must ensure this is documented in the patient's medical notes and the patient is re-consented.
The Sponsor and Chief/Principal Investigator must ensure that the trial is carried out in accordance with the GCP principles and requirements of the UK Clinical Trials regulations (SI 2004/1031 and SI 2006/1928 as amended).

149 Indemnity

This trial is being carried out under the auspices of The Institute of Cancer Research. The Institute of Cancer Research will provide patients with compensation for adverse side effects and has in place no-fault compensation insurance for any potential injury caused by participation in this clinical trial. Indemnity for participating hospitals is provided by the usual NHS indemnity arrangements for clinical negligence.

150 Publication policy and press releases

The trial results will be submitted for publication in a relevant medical journal with authorship according to the criteria defined by the ICMJE (http://www.icmje.org). These state that: Authorship credit should be based 1) substantial contributions to conception and design, acquisition of data, or analysis and interpretation of data; 2) drafting the article or revising it critically for important intellectual content; and 3) final approval of the version to be published. Authors should meet conditions 1, 2, and 3. Draft publications (manuscripts, abstracts, slides and posters) should be submitted to the DDU Clinical Trial Coordinator for circulation to the relevant parties and the ICR-CTSU to allow sufficient time for review prior to submission.
The DDU shall have for abstracts, slides and posters a twenty-one (21) day period and for manuscripts a thirty (30) day period to respond to the author with any revisions.

151 REFERENCES

1. Jemal A, Siegel R, Xu J, Ward E. Cancer statistics, 2010. CA: a cancer journal for clinicians. 2010;60(5):277-300.

2. Hellerstedt BA, Pienta KJ. The current state of hormonal therapy for prostate cancer. CA: a cancer journal for clinicians. 2002;52(3):154-79.

3. Tannock IF, Osoba D, Stockler MR, Ernst DS, Neville AJ, Moore MJ, et al. Chemotherapy with mitoxantrone plus prednisone or prednisone alone for symptomatic hormone-resistant prostate cancer:

a Canadian randomized trial with palliative end points. Journal of clinical oncology : official journal of the American Society of Clinical Oncology. 1996;14(6):1756-64.

4. Berthold DR, Pond GR, Soban F, de Wit R, Eisenberger M, Tannock IF. Docetaxel plus prednisone or mitoxantrone plus prednisone for advanced prostate cancer: updated survival in the TAX 327 study. Journal of clinical oncology : official journal of the American Society of Clinical Oncology. 2008;26(2):242-5.

5. Kantoff PW, Higano CS, Shore ND, Berger ER, Small EJ, Penson DF, et al. Sipuleucel-T immunotherapy for castration-resistant prostate cancer. The New England journal of medicine. 2010;363(5):411-22.

6. de Bono JS, Logothetis CJ, Molina A, Fizazi K, North S, Chu L, et al. Abiraterone and increased survival in metastatic prostate cancer. The New England journal of medicine. 2011;364(21):1995-2005.

7. de Bono JS, Oudard S, Ozguroglu M, Hansen S, Machiels JP, Kocak I, et al. Prednisone plus cabazitaxel or mitoxantrone for metastatic castration-resistant prostate cancer progressing after docetaxel treatment: a randomised open-label trial. Lancet. 2010;376(9747):1147-54.

8. Scher HI, Fizazi K, Saad F, Taplin ME, Sternberg CN, Miller K, et al. Increased survival with enzalutamide in prostate cancer after chemotherapy. The New England journal of medicine. 2012;367(13):1187-97.

9. Templeton AJ, McNamara MG, Seruga B, Vera-Badillo FE, Aneja P, Ocana A, et al. Prognostic role of neutrophil-to-lymphocyte ratio in solid tumors: a systematic review and meta-analysis. Journal of the National Cancer Institute. 2014;106(6):dju124.

10. Leibowitz-Amit R, Templeton AJ, Omlin A, Pezaro C, Atenafu EG, Keizman D, et al. Clinical variables associated with PSA response to abiraterone acetate in patients with metastatic castration-resistant prostate cancer. Annals of oncology : official journal of the European Society for Medical Oncology / ESMO. 2014;25(3):657-62.

11. Lorente D, Mateo J, Templeton AJ, Zafeiriou Z, Bianchini D, Ferraldeschi R, et al. Baseline neutrophil-lymphocyte ratio (NLR) is associated with survival and response to treatment with second-line chemotherapy for advanced prostate cancer independent of baseline steroid use. Annals of oncology : official journal of the European Society for Medical Oncology / ESMO. 2015;26(4):750-5.

12. Youn JI, Kumar V, Collazo M, Nefedova Y, Condamine T, Cheng P, et al. Epigenetic silencing of retinoblastoma gene regulates pathologic differentiation of myeloid cells in cancer. Nature immunology. 2013;14(3):211-20.

13. Idorn M, Kollgaard T, Kongsted P, Sengelov L, Thor Straten P. Correlation between frequencies of blood monocytic myeloid-derived suppressor cells, regulatory T cells and negative prognostic markers in patients with castration-resistant metastatic prostate cancer. Cancer immunology, immunotherapy : CII. 2014;63(11):1177-87.

14. Di Mitri D, Toso A, Chen JJ, Sarti M, Pinton S, Jost TR, et al. Tumour-infiltrating Gr-1+ myeloid cells antagonize senescence in cancer. Nature. 2014;515(7525):134-7.

15. Chi N, Tan Z, Ma K, Bao L, Yun Z. Increased circulating myeloid-derived suppressor cells correlate with cancer stages, interleukin-8 and -6 in prostate cancer. International journal of clinical and experimental medicine. 2014;7(10):3181-92.

16. Brusa D, Simone M, Gontero P, Spadi R, Racca P, Micari J, et al. Circulating immunosuppressive cells of prostate cancer patients before and after radical prostatectomy: profile comparison. International journal of urology : official journal of the Japanese Urological Association. 2013;20(10):971-8.

17. Beer TM, Armstrong AJ, Rathkopf DE, Loriot Y, Sternberg CN, Higano CS, et al. Enzalutamide in metastatic prostate cancer before chemotherapy. The New England journal of medicine. 2014;371(5):424-33.

18. Kirsten AM, Forster K, Radeczky E, Linnhoff A, Balint B, Watz H, et al. The safety and tolerability of oral AZD5069, a selective CXCR2 antagonist, in patients with moderate-to-severe COPD. Pulmonary pharmacology & therapeutics. 2015;31:36-41.

19. de Bono JS, Scher HI, Montgomery RB, Parker C, Miller MC, Tissing H, et al. Circulating tumor cells predict survival benefit from treatment in metastatic castration-resistant prostate cancer. Clinical cancer research : an official journal of the American Association for Cancer Research. 2008;14(19):6302-9.

20. Olmos D, Brewer D, Clark J, Danila DC, Parker C, Attard G, et al. Prognostic value of blood mRNA expression signatures in castration-resistant prostate cancer: a prospective, two-stage study. The Lancet Oncology. 2012;13(11):1114-24.

152 APPENDICES

153 APPENDIX 1: WHO PERFORMANCE SCALE

Activity Performance Description	Score
Fully active, able to carry out all normal activity without restriction.	0
Restricted in physically strenuous activity but ambulatory and able to carry out work of a light or sedentary nature, for example, light housework, office work.	1
Ambulatory and capable of all self-care, but unable to carry out any work activities. Up and about more than 50% of waking hours.	2
Capable of only limited self-care. Confined to bed or chair more than 50% of waking hours.	3
Completely disabled. Cannot carry out any self-care. Totally confined to bed or chair.	4

154 APPENDIX 2: DECLARATION OF HELSINKI

Recommendations guiding physicians in biomedical research involving human subjects

Adopted by the 18th World Medical Assembly,
Helsinki, Finland, June 1964
amended by the 29th World Medical Assembly
Tokyo, Japan, October 1975,
and
the 35th World Medical Assembly,
Venice, Italy, October 1983
and
the 41st World Medical Assembly,
Hong-Kong, September 1989
and
the 48th General Assembly,
Somerset West, Republic of South Africa, October 1996

INTRODUCTION

It is the mission of the physician to safeguard the health of the people. His or her knowledge and conscience are dedicated to the fulfilment of this mission.

The Declaration of Geneva of the World Medical Association binds the physician with the words, “The health of my patient will be my first consideration”, and the International Code of Medical Ethics declares that, “A physician shall act only in patient’s interest when providing medical care which might have the effect of weakening the physical and mental condition of the patient”.

The purpose of biomedical research involving human subjects must be to improve diagnostic, therapeutic and prophylactic procedures and the understanding of the aetiology and pathogenesis of disease.

In current medical practice most diagnostic, therapeutic or prophylactic procedures involve hazards. This applies especially to biomedical research.

Medical progress is based on research which ultimately must rest in part on experimentation involving human subjects.

In the field of biomedical research, a fundamental distinction must be recognised between medical research in which the aim is essentially diagnostic or therapeutic for a patient, and medical research, the essential object of which is purely scientific

and without implying direct diagnostic or therapeutic value to the person subjected to the research.

Special caution must be exercised in the conduct of research which may affect the environment, and the welfare of animals used for research must be respected.

Because it is essential that the results of laboratory experiments be applied to human beings to further scientific knowledge and to help suffering humanity, the World Medical Association has prepared the following recommendations as a guide to every physician in biomedical research involving human subjects. They should be kept under review in the future. It must be stressed that the standards as drafted are only a guide to physicians all over the world. Physicians are not relieved from criminal, civil and ethical responsibilities under the laws of their own countries.

I - BASIC PRINCIPLES

1. Biomedical research involving human subjects must conform to generally accepted scientific principles and should be based on adequately performed laboratory and animal experimentation and on a thorough knowledge of the scientific literature.

2. The design and performance of each experimental procedure involving human subjects should be clearly formulated in an experimental protocol which should be transmitted for consideration, comment and guidance to a specially appointed committee independent of the investigator and the sponsor provided that this independent committee is in conformity with the laws and regulations of the country in which the research experiment is performed.

3. Biomedical research involving human subjects should be conducted only by scientifically qualified persons and under the supervision of a clinically competent medical person. The responsibility for the human subject must always rest with a medically qualified person and never rest on the subject of the research, even though the subject has given his or her consent.

4. Biomedical research involving human subjects cannot legitimately be carried out unless the importance of the objective is in proportion to the inherent risk to the subject.

5. Every biomedical research project involving human subjects should be preceded by careful assessment of predictable risks in comparison with foreseeable benefits to the subject or to others. Concern for the interests of the subject must always prevail over the interests of science and society.

6. The right of the research subject to safeguard his or her integrity must always be respected. Every precaution should be taken to respect the privacy of the subject

to minimise the impact of the study on the subject's physical and mental integrity and on the personality of the subject.

7. Physicians should abstain from engaging in research projects involving human subjects unless they are satisfied that the hazards involved are believed to be predictable. Physicians should cease any investigation if the hazards are found to outweigh the potential benefits.

8. In publication of the results of his or her research, the physician is obliged to preserve the accuracy of the results. Reports of experimentation not in accordance with the principles laid down in this Declaration should not be accepted for publication.

9. In any research on human beings, each potential subject must be adequately informed of the aims, methods, anticipated benefits and potential hazards of the study and the discomfort it may entail. He or she should be informed that he or she is at liberty to abstain from participation in the study and that he or she is free to withdraw his or her consent to participation at any time. The physician should then obtain the subject's freely given informed consent, preferably in writing.

10. When obtaining informed consent for the research project the physician should be particularly cautious if the subject is in a dependent relationship to him or her or may consent under duress. In that case the informed consent should be obtained by a physician who is not engaged in the investigation and who is completely independent of this official relationship.

11. In case of legal incompetence, informed consent should be obtained from the legal guardian in accordance with national legislation. Where physical or mental incapacity makes it impossible to obtain informed consent, or when the subject is a minor, permission from the responsible relative replaces that of the subject in accordance with national legislation.

12. The research protocol should always contain a statement of the ethical considerations involved and should indicate that the principles enunciated in the present Declaration are complied with.

II - MEDICAL RESEARCH COMBINED WITH PROFESSIONAL CARE (Clinical research)

1. In the treatment of the sick person, the physician must be free to use a new diagnostic and therapeutic measure, if in his or her judgement it offers hope of saving life, re-establishing health or alleviating suffering.

2. The potential benefits, hazards and discomfort of a new method should be

weighed against the advantages of the best current diagnostic and therapeutic methods.

3. In any medical study, every patient - including those of a control group, if any - should be assured of the best proven diagnostic and therapeutic method. This does not exclude the use of inert placebo in studies where no proven diagnostic or therapeutic method exists.

4. The refusal of the patient to participate in a study must never interfere with the physician-patient relationship.

5. If the physician considers it essential not to obtain informed consent, the specific reasons for this proposal should be stated in the experimental protocol for transmission to the independent Committee (1,2).

6. The physician can combine medical research with professional care, the objective being the acquisition of new medical knowledge, only to the extent that medical research is justified by its potential diagnostic or therapeutic value for the patient.

III - <u>NON-THERAPEUTIC BIOMEDICAL RESEARCH INVOLVING HUMAN SUBJECTS</u>
(Non-clinical biomedical research)

1. In the purely scientific application of medical research carried out on a human being, it is the duty of the physician to remain the protector of the life and health of that person on whom biomedical research is being carried out.

2. The subjects should be volunteers - either healthy persons or patients for whom the experimental design is not related to the patient's illness.

3. The investigator or the investigating team should discontinue the research if in his/her or their judgement it may, if continued, be harmful to the individual.

4. In research on man, the interest of science and society should never take precedence over considerations related to the well-being of the subject.

155 APPENDIX 3: MEASUREMENT OF DISEASE

Response Evaluation Criteria In Solid Tumours: Revised RECIST Guideline (version 1.1) [Eisenhauer, 2009]

Baseline documentation of target and non-target lesions

- All baseline lesion assessments must be performed within 28 days of randomizations.
- Lymph nodes that have a short axis of <10mm are considered non-pathological and should not be recorded or followed.
- Pathological lymph nodes with <15mm and but 10mm short axis are considered non measurable.
- Pathological lymph nodes with 15mm short axis are considered measurable and can be selected as target lesions, however lymph nodes should not be selected as target lesions when other suitable target lesions are available.
- Measurable lesions up to a maximum of 2 lesions per organ and 5 lesions in total, representative of all involved organs, should be identified as target lesions, and recorded and measured at baseline. These lesions should be selected on the basis of their size (lesions with the longest diameter) and their suitability for accurate repeated measurements (either by imaging techniques or clinically).

Note: Cystic lesions thought to represent cystic metastases should not be selected as target lesions when other suitable target lesions are available.

Note: Measurable lesions that have been previously irradiated and have not been shown to be progressing following irradiation should not be considered as target lesions.

- All other lesions (or sites of disease) should be identified as non-target and should also be recorded at baseline. Non-target lesions will be group by organ. Measurements of these lesions are not required, but the presence or absence of each should be noted throughout follow-up.

Efficacy assessment

Disease progression and response evaluations will be determined according to the definitions established in the Response Evaluation Criteria in Solid Tumors (RECIST 1.1) [Eisenhauer, 2009].

See the Time and Events Table (Section 3.8) for the schedule of efficacy assessments. Assessments must be performed on a calendar schedule and should not be affected by dose interruptions/delays. For post baseline assessments, a window of 14 days is permitted to allow for flexible scheduling.

- The following are required at baseline: CT for Chest/Abdomen/Pelvis or MRI for Abdomen/Pelvis and clinical disease assessment for palpable lesions. At each post baseline assessment, evaluations of the sites of disease identified by these scans are required except for brain scan and bone scans. Brain and Bone scans should be performed as clinically indicated.

Confirmation of CR and PR are required per protocol. Confirmation assessments must be performed no less than 4 weeks after the criteria for response have initially been met and may be performed at the next protocol scheduled assessment. If a confirmation assessment is performed prior to the next protocol schedule assessment, the next protocol scheduled evaluation is still required (*e.g*. evaluations must occur at each protocol scheduled time point regardless of unscheduled assessments).

For subjects not known to have CNS disease at baseline brain scans should only be performed as clinically indicated (e.g. symptoms suggestive of CNS progression). For subjects with known CNS disease, a baseline brain scan is required and subsequent scans should be performed as clinically indicated.

Assessment guidelines

Please note the following:

- The same diagnostic method, including use of contrast when applicable, must be used throughout the study to evaluate a lesion.
- All measurements should be taken and recorded in millimetres (mm), using a ruler or calipers.
- Ultrasound is not a suitable modality of disease assessment. If new lesions are identified by ultrasound, confirmation by CT or MRI is required.

Clinical Examination: Clinically detected lesions will only be considered measurable when they are superficial (*e.g.*, skin nodules). In the case of skin lesions, documentation by colour photography, including a ruler/calipers to measure the size of the lesion, is required. [Eisenhauer, 2009].

CT and MRI: Contrast enhanced CT with 5mm contiguous slices is recommended.
Minimum size of a measurable baseline lesion should be twice the slice thickness, with a minimum lesion size of 10 mm when the slice thickness is 5 mm. MRI is acceptable, but when used, the technical specification of the scanning sequences should be optimised for the evaluation of the type and site of disease and lesions must be measured in the same anatomic plane by use of the same imaging examinations. Whenever possible the same scanner should be used. [Eisenhauer, 2009].

X-ray: In general, X-ray should not be used for target lesion measurements owing to poor lesion definition. Lesions on chest X-ray may be considered measurable if they are clearly defined and surrounded by aerated lung; however, chest CT is preferred over chest X-ray [Eisenhauer, 2009].

Brain Scan: Baseline brain scans are required, then contrast enhanced MRI is preferable to contrast enhanced CT.

Guidelines for Evaluation of Disease

Measurable and Non-measurable Definitions

Measurable lesion: A non-nodal lesion that can be accurately measured in at least one dimension (longest dimension) of

10 mm with MRI or CT when the scan slice thickness is no greater than 5mm. If the slice thickness is greater than 5mm, the minimum size of a measurable lesion must be at least double the slice thickness (*e.g.*, if the slice thickness is 10 mm, a measurable lesion must be 20 mm).

10 mm calliper/ruler measurement by clinical exam or medical photography.

20 mm by chest x-ray.

Additionally lymph nodes can be considered pathologically enlarged and measurable if 15mm in the short axis when assessed by CT or MRI (slice thickness recommended to be no more than 5mm). At baseline and follow-up, only the short axis will be measured [Eisenhauer, 2009].

Non-measurable lesion: All other lesions including lesions too small to be considered measurable (longest diameter <10 mm or pathological lymph nodes with 10 mm and <15 mm short axis) as well as truly non-measurable lesions, which include: leptomeningeal disease, ascites, pleural or pericardial effusions, inflammatory breast disease, lymphangitic involvement of the skin or lung, abdominal masses/abdominal organomegaly identified by physical exam that is not measurable by reproducible imaging techniques [Eisenhauer, 2009].

Measurable disease: The presence of at least one measurable lesion. Palpable lesions that are not measurable by radiologic or photographic evaluations may not be utilized as the only measurable lesion.

Non-Measurable only disease: The presence of only non-measurable lesions. Note: non-measurable only disease is not allowed per protocol.

Response Criteria

Evaluation of target lesions

Definitions for assessment of response for target lesion(s) are as follows:

- Complete Response (CR): Disappearance of all target lesions. Any pathological lymph nodes must be <10mm in the short axis.

- Partial Response (PR): At least a 30% decrease in the sum of the diameters of target lesions, taking as a reference, the baseline sum of the diameters (*e.g*. percent change from baseline).

- Stable Disease (SD): Neither sufficient shrinkage to qualify for PR nor sufficient increase to qualify for progressive disease.

- Progressive Disease (PD): At least a 20% increase in the sum of the diameters of target lesions, taking as a reference, the smallest sum of diameters recorded since the treatment started (*e.g*. percent change from nadir, where nadir is defined as the smallest sum of diameters recorded since treatment start). In addition, the sum must have an absolute increase from nadir of 5mm.

- Not Applicable (NA): No target lesions at baseline.

- Not Evaluable (NE): Cannot be classified by one of the five preceding definitions.

Note:

- If lymph nodes are documented as target lesions the short axis is added into the sum of the diameters (*e.g*. sum of diameters is the sum of the longest diameters for non-nodal lesions and the short axis for nodal lesions). When lymph nodes decrease to non-pathological size (short axis <10mm) they should still have a measurement reported in order not to overstate progression.

- If at a given assessment time point all target lesions identified at baseline are not assessed, sum of the diameters cannot be calculated for purposes of assessing CR, PR, or SD, or for use as the nadir for future assessments. However, the sum of the diameters of the assessed lesions and the percent change from nadir should be calculated to ensure that progression has not been documented. If an assessment of PD cannot be made, the response assessment should be NE.

- All lesions (nodal and non-nodal) should have their measurements recorded even when very small (*e.g.* 2 mm). If lesions are present but too small to measure, 5 mm should be recorded and should contribute to the sum of the diameters, unless it is likely that the lesion has disappeared in which case 0 mm should be reported.

- If a lesion disappears and reappears at a subsequent time point it should continue to be measured. The response at the time when the lesion reappears will depend upon the status of the other lesions. For example, if the disease had reached a CR status then PD would be documented at the time of reappearance. However, if the response status was PR or SD, the diameter of the reappearing lesion should be added to the remaining diameters and response determined based on percent change from baseline and percent change from nadir.

Evaluation of non-target lesions

Definitions for assessment of response for non-target lesions are as follows:

- Complete Response (CR): The disappearance of all non-target lesions. All lymph nodes identified as a site of disease at baseline must be non-pathological (*e.g*. <10 mm short axis).

- Non-CR/Non-PD: The persistence of 1 or more non-target lesion(s) or lymph nodes identified as a site of disease at baseline ≥ 10 mm short axis.

- Progressive Disease (PD): Unequivocal progression of existing non-target lesions.

- Not Applicable (NA): No non-target lesions at baseline.

- Not Evaluable (NE): Cannot be classified by one of the four preceding definitions.

Note:

- In the presence of measurable disease, progression on the basis of solely non-target disease requires substantial worsening such that even in the presence of SD or PR in target disease, the overall tumour burden has increased sufficiently to merit discontinuation of therapy.

- Sites of non-target lesions, which are not assessed at a particular timepoint based on the assessment schedule, should be excluded from the response determination (*e.g*. non-target response does not have to be "Not Evaluable").

New lesions

New malignancies denoting disease progression must be unequivocal. Lesions identified in follow-up in an anatomical location not scanned at baseline are considered new lesions.
Any equivocal new lesions should continue to be followed. Treatment can continue at the discretion of the investigator until the next scheduled assessment. If at the next assessment the new lesion is considered to be unequivocal, progression should be documented.

Evaluation of overall response

Table 22 presents the overall response at an individual time point for all possible combinations of tumour responses in target and non-target lesions with or without the appearance of new lesions for subjects with measurable disease at baseline.

Table 22 Evaluation of Overall Response for Subjects with Measurable Disease at Baseline

Target Lesions	Non-Target Lesions	New Lesions	Overall Response
CR	CR or NA	No	CR
CR	Non-CR/Non-PD or NE	No	PR
PR	Non-PD or NA or NE	No	PR
SD	Non-PD or NA or NE	No	SD
NE	Non-PD or NA or NE	No	NE
PD	Any	Yes or No	PD
Any	PD	Yes or No	PD
Any	Any	Yes	PD

CR=complete response, PR = partial response, SD=stable disease, PD=progressive disease, NA= Not applicable, and NE=Not Evaluable

Note:

Subjects with a global deterioration of health status requiring discontinuation of treatment without objective evidence of disease progression at that time should be classified as having "symptomatic deterioration." Objective response status is

determined by evaluations of disease burden. Every effort should be made to document the objective progression even after discontinuation of treatment.

In some circumstances, it may be difficult to distinguish residual disease from normal tissue. When the evaluation of CR depends on this determination, it is recommended that the residual lesion be investigated (fine needle aspirate/biopsy) to confirm the CR.

Evaluation of best overall response

The best overall response is the best response recorded from the start of the treatment until disease progression/recurrence and will be determined programmatically by GSK based on the investigator's assessment of response at each time point.

To be assigned a status of SD, follow-up disease assessment must have met the SD criteria at least once after first dose at a minimum interval of 4 weeks.

If the minimum time for SD is not met, best response will depend on the subsequent assessments. For example, if an assessment of PD follows the assessment of SD and SD does not meet the minimum time requirement the best response will be PD. Alternative subjects lost to follow-up after an SD assessment not meeting the minimum time criteria will be considered not evaluable.

Confirmation Criteria (recommended):

To be assigned a status of PR or CR, a confirmatory disease assessment should be performed no less than 4 weeks (28 days) after the criteria for response are first met.

156 APPENDIX 4: NEW YORK HEART ASSOCIATION (NYHA) SCALE

Class I – patients with cardiac disease but without resulting limitation of physical activity; ordinary physical activity does not cause undue dyspnoea (or fatigue, palpitation or anginal pain)
Class II – patients with cardiac disease resulting in slight limitation of physical activity; they are comfortable at rest; ordinary physical activity results in dyspnoea (or fatigue, palpitation or anginal pain)
Class III – patients with cardiac disease resulting in marked limitations of physical activity; they are comfortable at rest; less than ordinary physical activity causes dyspnoea (or fatigue, palpitation or anginal pain)
Class IV – patients with cardiac disease resulting in inability to carry out physical activity without discomfort; symptoms of dyspnoea (or of angina) may be present even at rest; if any physical activity is undertaken, discomfort is increased.

157 APPENDIX 5: POTENT/MODERATE CYP 3A4 INHIBITORS FOR PATIENTS ON AZD5069

Class	Examples (including, but not limited to)	
**Potent/moderate CYP 3A4 inhibitors**	amprenavir	imatinib
	aprepitant	indinavir
	atazanavir	indinavir/ritonavir
	atazanavir/ritonavir	itraconazole
	boceprevir	ketoconazole
	casopitant	ledipasvir
	cimetidine	lomitapide
	ciprofloxacin	lopinavir/ritonavir

	clarithromycin	mibefradil
	cobicistat (GS-9350)	nefazodone
	conivaptan	nelfinavir
	crizotinib	netupitant
	cyclosporine	posaconazole
	danoprevir/ritonavir	ritonavir
	darunavir	saquinavir
	darunavir/ritonavir	saquinavir/ritonavir
	diltiazem	schisandra sphenanthera
	dronedarone	telaprevir
	elvitegravir/ritonavir	telithromycin
	erythromycin	tipranavir/ritonavir
	fluconazole	tofisopam
	fosamprenavir	troleandomycin
	grapefruit juice	verapamil
	idelalisib	voriconazole
**Potent/moderate CYP 3A4 inducers**	avasimibe	modafinil
	bosentan	nafcillin
	carbamazepine	phenytoin
	efavirenz	rifampin
	etravirine	St. John's wort
**Sensitive CYP 2B6 substrates**	bupropion	efavirenz
**P-gp substrates with narrow therapeutic index**	digoxin	dabigatran
**Coumarin derivatives**	acenocoumarol	warfarin
	phenprocoumon	
**Breast cancer resistance protein (BCRP) substrates that reduce blood neutrophils**	topotecan	
**Any herbal medications**		
**Seville orange or grapefruit products**	Seville orange marmalade	grapefruit
	Seville orange juice	grapefruit juice
		grapefruit marmalade

For an exhaustive list of CYP inhibitors and inducers please refer to;

http://medicine.iupui.edu/clinpharm/ddis/table.aspx
