# Supplementary material for: Targeting myeloid chemotaxis to reverse prostate cancer therapy resistance
Source: Nature. 2023 Oct 16;623(7989):1053–61. doi: 10.1038/s41586-023-06696-z (PMC10686834; doi:10.1038/s41586-023-06696-z)
Supplement: Supplementary file 2 — Reporting Summary [file 41586_2023_6696_MOESM2_ESM.pdf]

nature portfolio

Corresponding author(s): Johann de Bono

Last updated by author(s): 08/31/2023

Reporting Summary

Nature Portfolio wishes to improve the reproducibility of the work that we publish. This form provides structure for consistency and transparency in reporting. For further information on Nature Portfolio policies, see our Editorial Policies and the Editorial Policy Checklist.

Statistics

For all statistical analyses, confirm that the following items are present in the figure legend, table legend, main text, or Methods section.

n/a	Confirmed	
☐	☒	The exact sample size (*n*) for each experimental group/condition, given as a discrete number and unit of measurement
☒	☐	A statement on whether measurements were taken from distinct samples or whether the same sample was measured repeatedly
☐	☒	The statistical test(s) used AND whether they are one- or two-sided<br>*Only common tests should be described solely by name; describe more complex techniques in the Methods section.*
☐	☒	A description of all covariates tested
☐	☒	A description of any assumptions or corrections, such as tests of normality and adjustment for multiple comparisons
☐	☒	A full description of the statistical parameters including central tendency (e.g. means) or other basic estimates (e.g. regression coefficient) AND variation (e.g. standard deviation) or associated estimates of uncertainty (e.g. confidence intervals)
☐	☒	For null hypothesis testing, the test statistic (e.g. *F*, *t*, *r*) with confidence intervals, effect sizes, degrees of freedom and *P* value noted<br>*Give P values as exact values whenever suitable.*
☒	☐	For Bayesian analysis, information on the choice of priors and Markov chain Monte Carlo settings
☒	☐	For hierarchical and complex designs, identification of the appropriate level for tests and full reporting of outcomes
☐	☒	Estimates of effect sizes (e.g. Cohen's *d*, Pearson's *r*), indicating how they were calculated

Our web collection on statistics for biologists contains articles on many of the points above.

Software and code

Policy information about availability of computer code

Data collection

Clinical trials data was collected in MACRO (v4.11.0.459).
SAE data was recorded in Safire (v3.0).
Translational singleplex immunohistochemical was collected using Progeny (v10).
CTC count data was collected in Microsoft Excel (v16.75).
PK and ELISA data were collected and stored using Microsoft Excel (v16.73).
Circulating cytokine data was collected using Luminex xPONENT (v3.1) and EnVision (v1.14.3049.528) and then output into Microsoft Excel v16.75).
Multiplex IF data was collected in Halo (v3.6.4134.137)
Hyperplex IF data was collected in the COMET Control Software (v 0.70.0.1) then imported and stored in Halo (v3.6.4134.137).

Data analysis

Statistical analysis for the clinical trial was performed using R software (v4.2.2) and according to the Statistical Analyses Plan for trial-related analyses.

Somatic variant calls from the Next-generation sequencing data were manually inspected in the Integrative Genomics Viewer (v2.16.1).

HTG data were analysed using the HTG EdgeSeq Parser Software (V5.3, HTG Molecular Diagnostics). Differential gene expression between pre and post treatment samples was performed using the HTG EdgeSeq Reveal DESeq2 analysis pipeline and R Software (v4.2.3).

The SU2C/PCF transcriptomes were aligned to the human reference genome (GRCh37/hg19) using TopHat2 (v2.0.7). Gene expression as

fragments per kilobase of transcript per million mapped reads (FPKM) was calculated using Cufflinks (v2.2.1).

For the single-cell transcriptomic data analyses, publicly available data was analysed in R Software (v4.1.3). The raw counts from the localized prostate cancer data were log normalized. Both datasets were processed with Seurat (v4.3.0) and underwent scaling, clustering, dimensional reduction, and cell type assignment with SingleR (v1.8.1) using the Blueprint ENCODE reference dataset from the celldex (v1.4.0) library.

Pharmacokinetic parameters were calculated using non-compartmental analysis (Phoenix v8.1, Certara).
IF data were analysed in Halo v3.6.4134.137) and R software (v4.2.2).
All correlative translational analyses and survival analyses were performed using R software (v4.2.2).
Correlative analyses of PK-PD-dose relationships were performed using R software (v4.2.2).

For manuscripts utilizing custom algorithms or software that are central to the research but not yet described in published literature, software must be made available to editors and reviewers. We strongly encourage code deposition in a community repository (e.g. GitHub). See the Nature Portfolio guidelines for submitting code & software for further information.

Data

Policy information about availability of data

All manuscripts must include a data availability statement. This statement should provide the following information, where applicable:

- Accession codes, unique identifiers, or web links for publicly available datasets
- A description of any restrictions on data availability
- For clinical datasets or third party data, please ensure that the statement adheres to our policy

Source data for the non-clinical trial related translational analyses are provided in the source data file associated with each figure (except for when the data was downloaded from the publicly available datasets listed below). The full study protocol is provided with this paper as part of the supplementary information.

Bulk RNASeq data from the SU2C/PCF cohort (https://www.pnas.org/doi/10.1073/pnas.1902651116) was downloaded from https://www.cbioportal.org/study/summary?id=prad_su2c_2019. Single-cell transcriptomic data from 15 mCRPC samples (https://www.nature.com/articles/s41591-021-01244-634) were downloaded from https://singlecell.broadinstitute.org/single_cell/study/SCP1244/transcriptional-mediators-of-treatment-resistance-in-lethal-prostate-cancer (study number SCP1244).

Single-cell transcriptomic data from 11 patients with localized prostate cancer (https://www.nature.com/articles/s41467-021-27322-435) were downloaded from https://www.ncbi.nlm.nih.gov/geo/query/acc.cgi?acc=GSE176031 (GEO accession GSE176031).

De-identified, bulk RNASeq data from the RMH cohort may be requested from the corresponding author (J.S.dB.). Request for data must provide clinically relevant rationale and will be reviewed by the corresponding author (J.S.dB.) to determine if the request is subject to any ethical and/or confidentiality considerations. Subject to patient privacy and confidentiality obligations, access to data from the clinical trial may be available upon request to the study sponsor (The ICR, ACE@icr.ac.uk) and corresponding author (J.S.dB). Request data must provide clinically relevant rationale in adherence with the intent of the study and patients' consent, will be reviewed by the study sponsor and the corresponding author (J.S.dB.) to determine if the request is subject to any intellectual property, ethical and/or confidentiality considerations, and will respond promptly. Patient identifiers or information that may reveal the patient's identity will not be shared owing to patient confidentiality. Any data or material that can be shared will be done via a material transfer agreement with The ICR.

Research involving human participants, their data, or biological material

Policy information about studies with human participants or human data. See also policy information about sex, gender (identity/presentation), and sexual orientation and race, ethnicity and racism.

Reporting on sex and gender	Since our study only included patients with histologically proven prostate cancer, all participants were of male sex. Gender data was not collected from any of the participants.
Reporting on race, ethnicity, or other socially relevant groupings	No socially constructed or socially relevant categorization variables were collected or used.
Population characteristics	For the clinical trial, 23 participants with histologically confirmed metastatic castration-resistant prostate cancer were enrolled. Participants were recruited from the following participating study sites: The Royal Marsden Hospital (UK), Oncology Institute of Southern Switzerland (Switzerland), and Belfast City Hospital Cancer Centre (Northern Ireland). Description of this cohort is presented in Extended Data Table 1. For the translational analyses of myeloid cell infiltration, we studied two cohorts of patients with metastatic prostate cancer were included. The first cohort (cohort 1) consisted of 48 mCRPC biopsies from patients treated at The ICR/RMH, IOSI, and Belfast City Hospital. The validation cohort consisted of another 57 mCRPC biopsies from patients treated at the RMH. Descriptions of these 2 cohorts are presented in Supplementary Table S1.
Recruitment	Participants were recruited from 3 oncology centres in Europe (The Royal Marsden Hospital, Belfast City Hospital, and Oncology Institute of Southern Switzerland). Patients with metastatic prostate cancer were either referred to the study site for consideration of participation in an oncology clinical trials by their oncologist or the patients were already under the care their treating oncologist located at the study site. The study investigator determined, in consultation with the patient, whether they wished to participate in an early phase clinical trial, and if there was no clear contraindication to participating in the trial, and the patient was interested in considering this option, the patient was provided with written and verbal information about the study. This information includes, but was not limited to: information about the study drugs and treatment schedule; the scientific and clinical rationale for the study; potential risks and benefits; assessments and schedule during screening, on trial, and after trial drug cessation; and how data and samples were to be stored, used, and disseminated. Patients were aware that participation in the study is entirely voluntary and that they could withdraw their consent at any point. Patients were made aware of potential alternatives to participating in the study, including opting for no

further active intervention. Patients were given subsequent opportunities for additional questions to be addressed with the study physician and ample time to make the decision.
Potential biases: only patients who were treated at or referred to one of the study sites, which were all cancer centres in Europe that conducted early phase trials, were included. All participants had to meet the specific inclusion/exclusion criteria of the trial which excluded patients with worse performance status, major organ dysfunction, history of other malignancies, and comorbidities (see details in the Methods section). A part of the study was conducted during the Covid-19 pandemic, which may impact some patients' willingness to participate in clinical trials. All of these factors may have impacted the generalizability of these findings to the broader, global, advanced prostate cancer population, and therefore the findings of our study warrant further evaluation in other contexts, and with larger and more diverse cohorts.

Ethics oversight

Clinical trial oversight: The study was conducted in accordance with the provisions of the Declaration of Helsinki and Good Clinical Practice guidelines. Regulatory approvals were obtained from the Medicines Healthcare products Regulatory Agency (MHRA), Swiss Swissmedic, and local Research Ethics Committees (London-Surrey Borders REC for the Royal Marsden Hospital and Belfast City Hospital; Comitato Etico Cantonale Bellinzona for the Oncology Institute of Southern Switzerland in Switzerland).

Written informed consent was obtained from all participants. No participant compensation was provided. A safety review committee evaluated the safety and tolerability at regular intervals after recruitment of three patients to a schedule. All protocol amendments were approved by the trial sponsor, MHRA, Swissmedic, and UK and Swiss RECs (London-Surrey Borders REC for the Royal Marsden Hospital and Belfast City Hospital; Comitato Etico Cantonale Bellinzona for the Oncology Institute of Southern Switzerland in Switzerland).

The study was sponsored and monitored by The Institute of Cancer Research (The ICR), UK. The study was registered on ClinicalTrials.gov before commencement (ClinicalTrials.gov identifier: NCT03177187, EudraCT: 2016-003141-28).

All patients included in the translational analyses provided informed consent, and enrolled onto institutional protocols approved by the local RECs at The Royal Marsden Hospital, Belfast City Hospital, and Institute of Oncology of Southern Switzerland.

Note that full information on the approval of the study protocol must also be provided in the manuscript.

Field-specific reporting

Please select the one below that is the best fit for your research. If you are not sure, read the appropriate sections before making your selection.

☒ Life sciences ☐ Behavioural & social sciences ☐ Ecological, evolutionary & environmental sciences

For a reference copy of the document with all sections, see nature.com/documents/nr-reporting-summary-flat.pdf

Life sciences study design

All studies must disclose on these points even when the disclosure is negative.

Sample size

For the phase 1 trial, sample size was chosen pragmatically. According to the rule-based 3+3 design, which guided dose-escalation decisions, the cohort size was three patients and skipping of dose levels in dose-escalation was not allowed. For the translational studies, sample size was also determined pragmatically based on available prostate cancer biopsy cohorts as well as the size of the existing RNASeq datasets.

Data exclusions

No data was excluded. Analyses were performed as per study protocol. The evaluable populations were defined as per protocol.

Replication

For the translational analyses pertaining to associations between NLR and neutrophils and myeloid cell count as well as NLR and neutrophils and myeloid gene signatures, and survival analyses, a second independent cohort was analysed to validate the initial findings.

Randomization

The study utilised the 3+3 design where participants were enrolled sequentially to escalating dose levels, and does not allow for randomisation.

Blinding

Blinding from patients: It would not have been clinically acceptable to the patient or their physician to administer placebos (inactive treatments) in the context of a phase 1 oncology clinical trial where the trial is logistically intensive, carry uncertainties, and the patients have limited life expectancies, and therapeutic options, therefore all patients were assigned to receive the active study drug. Patients were not blinded to the dose-level they were receiving. Given this is a phase 1 study testing a drug combination that has not previously been assessed, we also wanted to be transparent with patients about the dose level they were to receive, and the emerging clinical experience, as this could factor into patients' risk-benefit considerations for whether to participate in an early phase clinical trial.

Blinding from investigators: It would not be feasible or safe to blind the dose level from the investigators in a 3+3 design phase 1 trial given investigators participate in safety review meetings where dose-escalation decisions are made and patients are enrolled sequentially to escalating dose levels.

Reporting for specific materials, systems and methods

We require information from authors about some types of materials, experimental systems and methods used in many studies. Here, indicate whether each material, system or method listed is relevant to your study. If you are not sure if a list item applies to your research, read the appropriate section before selecting a response.

Materials & experimental systems

n/a	Involved in the study
☐	☒ Antibodies
☐	☒ Eukaryotic cell lines
☒	☐ Palaeontology and archaeology
☒	☐ Animals and other organisms
☐	☒ Clinical data
☒	☐ Dual use research of concern
☒	☐ Plants

Methods

n/a	Involved in the study
☒	☐ ChIP-seq
☒	☐ Flow cytometry
☒	☐ MRI-based neuroimaging

Antibodies

Antibodies used

HYPERPLEX IF
--- Primary antibodies (see Supplementary information for further details)
Listed in order of marker, vendor, cat number, clone, lot number
NCAM1 (CD56), Abcam, ab270248, 123C3.D5, 1020624-1
CXCR2, Abcam, ab245982, EPR22301-103, GR3378654-6
FOXP3, eBioscience™ (Thermo Fisher), 14-4777-82, 236A/E7, 2378013
CD15, Dako, M3631, Carb-3, 11397463
Granzyme B, CST, 46890, D6E9W, 6
CD14, Abcam, ab133503, EPR3652, GR211954-7
CD138, Dako, M7228, MI15, 41415171
CD11b, Abcam, ab52478, EP1345Y, GR3219233-5
MUM1, Dako, M7259, MUM1p, 41455977
CD8, Dako, M7103, C8/144B, 41389238
CD163, Abcam, ab182422, EPR19518, GR3339055-17
CD68, Dako, M0876, PG-M1, 41337737
Chromogranin A, Dako, M0869, DAK-A3, 41449632
HLA-DR, Abcam, ab20181, TAL 1B5, GR3378141-4
CD4, Abcam, ab133616, EPR6855, GR3276764-30
Pan-cytokeratin, Dako, M3515, AE1/AE3, 11445606
CD20, Dako, M0755, L26, 41367309
CD38, Abcam, ab226034, EPR4106, GR3402044-1
Synaptophysin, Leica Biosystems, SYNAP-299-L-CE, 27G12, 6081141
CD206/MRC1, CST, 91992, E2L9N, 1
--- Secondary antibodies
Listed in order of marker, vendor, cat number, clone, lot number
Goat anti-Mouse IgG (H+L) Highly Cross-Adsorbed Secondary Antibody, Alexa Fluor™ Plus 555, Invitrogen (Thermo Fisher Scientific), A32727, Polyclonal, WL333735
Goat anti-Rabbit IgG (H+L) Highly Cross-Adsorbed Secondary Antibody, Alexa Fluor™ Plus 647, Invitrogen (Thermo Fisher Scientific), A32733, Polyclonal, WL333739

MULTICOLOR IF
--- Primary antibodies
Listed in order of marker, vendor, cat number, clone, lot number
CXCR2, Abcam, ab245982, EPR22301-103, GR3378654-5
CD11b, Abcam, ab52478, EP1345Y, GR3219233-10
CD15, Dako, M3631, Carb-3, 11397463
CD14, Abcam, ab133503, EPR3652, GR211954-8
HLA-DR, Abcam, ab20181, TAL.1B5, GR3456090-1

-- Detection System:
Novocastra Post Primary, Leica Biosystems, Novolink Max Polymer Detection System, RE7280-K, 6098453
Novolink Polymer, Leica Biosystems, Novolink Max Polymer Detection System, RE7280-K, 6098453
TSA Coumarin, Akoya Biosciences, 1:50, NEL703001KT, 20220408
OPAL 520, Akoya Biosciences, 1:300, NEL820001KT, 20212719
OPAL 570 Akoya Biosciences, 1:300, NEL820001KT, 20213431
OPAL 650, Akoya Biosciences, 1:300, FP1496001KT, 20211913
TSA-DIG, Akoya Biosciences, 1:200, FP1501001KT, 20212315
OPAL 780, Akoya Biosciences, 1:50, FP1501001KT, 20213604

SINGLEPLEX IHC
Listed in order of marker, vendor, cat number, clone, lot number
PTEN, CST, Rabbit, 9559, 138G6, 17 and lot 19
AR-V7, Revmab, Rabbit, 31-1109-00, RM7, S-08-02447 and V-06-05221

Validation

https://www.abcam.com/products/primary-antibodies/ncam1-antibody-123c3d5-ab270248.html
https://www.abcam.com/products/primary-antibodies/cxcr2-antibody-epr22301-103-bsa-and-azide-free-ab245982.html
https://www.thermofisher.com/antibody/product/FOXP3-Antibody-clone-236A-E7-Monoclonal/14-4777-82
https://www.agilent.com/en/product/immunohistochemistry/antibodies-controls/primary-antibodies/cd15-%28concentrate%

29-76600
https://www.cellsignal.com/products/primary-antibodies/granzyme-b-d6e9w-rabbit-mab/46890
https://www.abcam.com/products/primary-antibodies/cd14-antibody-epr3652-ab133503.html
https://www.agilent.com/en/product/immunohistochemistry/antibodies-controls/primary-antibodies/cd138-%28concentrate%29-76642
https://www.abcam.com/products/primary-antibodies/cd11b-antibody-ep1345y-c-terminal-ab52478.html
https://www.agilent.com/en/product/immunohistochemistry/antibodies-controls/primary-antibodies/mum1-protein-%28concentrate%29-76652
https://www.agilent.com/en/product/immunohistochemistry/antibodies-controls/primary-antibodies/cd8-%28concentrate%29-76631
https://www.abcam.com/products/primary-antibodies/cd163-antibody-epr19518-ab182422.html
https://www.agilent.com/en/product/immunohistochemistry/antibodies-controls/primary-antibodies/cd68-%28concentrate%29-76550
https://www.agilent.com/en/product/immunohistochemistry/antibodies-controls/primary-antibodies/chromogranin-a-%28concentrate%29-76546
https://www.abcam.com/products/primary-antibodies/hla-dr-antibody-tal-1b5-ab20181.html
https://www.abcam.com/products/primary-antibodies/cd4-antibody-epr6855-ab133616.html
https://www.agilent.com/en/product/immunohistochemistry/antibodies-controls/primary-antibodies/cytokeratin-%28concentrate%29-76562
https://www.agilent.com/en/product/immunohistochemistry/antibodies-controls/primary-antibodies/cd20cy-%28concentrate%29-76520
https://www.abcam.com/products/primary-antibodies/cd38-antibody-epr4106-bsa-and-azide-free-ab226034.html
https://shop.leicabiosystems.com/en-gb/ihc-ish/ihc-primary-antibodies/pid-synaptophysin
https://www.cellsignal.com/products/primary-antibodies/cd206-mrc1-e2l9n-rabbit-mab/91992

https://www.cellsignal.com/products/primary-antibodies/pten-138g6-rabbit-mab/9559?_requestid=2919710
https://www.revmab.com/index.php/product/anti-androgen-receptor-ar-v7-specific-rabbit-monoclonal-antibody-clone-rm7-arv7-splice-variant/?gclid=EAIaIQobChMIhK_L98TzgAMVkM7CBB13HQf1EAAYASAAEgK8bfD_BwE

Antibodies against CXCR2, FOXP3, CD15, CD14, CD138, CD11b, MUM1, CD163, CD68, HLA-DR, CD4, CD38, CD206, CD8, and granzyme-B were validated by Western blot comparing detection of protein expression in whole cell lysates treated with either nontargeting control siRNA or ON-TARGETplus pooled siRNA against the target protein (Dharmacon) or using positive and negative control cell lines. All markers were validated for appropriate tissue localisation on immunohistochemical staining of relevant positive and negative tissue controls and reviewed by a certified pathologist (B.G.). Validation for PTEN, CD4, CD8, FOXP3, CD11b, CD15, CD138, CD20, synaptophysin, chromogranin and AR-V7 were as previously described. IHC was performed on FFPE tissue sections using an automated staining platform (Bond RX, Leica Biosystems). Bone biopsies were decalcified using pH 7 EDTA for 48 hours at 37oC. Once validated for target sensitivity and specificity, the antibodies were further optimised for IHC, multi-/hyperplex IF using methods described below. The full list of antibodies, working dilutions, and incubation times are in Supplementary Tables S7 and S8.

Eukaryotic cell lines

Policy information about cell lines and Sex and Gender in Research

Cell line source(s)	All cell lines were purchased from ATCC (https://www.atcc.org). Cell lines used for antibody validation include: LNCaP, HL-60, HeLa, Daudi, Thp1, Jurkat, HDML2, A-431, A549, TIME
Authentication	Cell lines were authenticated by STR profiling prior to use
Mycoplasma contamination	All cells were regularly tested for mycoplasma contamination and were negative (every 3 months).
Commonly misidentified lines (See ICLAC register)	No commonly misidentified cell lines were use.

Clinical data

Policy information about clinical studies
All manuscripts should comply with the ICMJE guidelines for publication of clinical research and a completed CONSORT checklist must be included with all submissions.

Clinical trial registration	NCT03177187
Study protocol	Submitted with the manuscript
Data collection	The trial was conducted at three centres in Europe (Royal Marsden Hospital (UK), Belfast City Hospital (UK), The Oncology Institute of Southern Switzerland (Switzerland). The trial was conducted between November 2017 and November 2022.
Outcomes	Primary endpoint:<br>1. To identify the dose-limiting toxicities (DLTs), estimate the maximum tolerated dose (MTD) and identify the recommended phase II dose (RP2D) or the recommended phase II doses (RP2Ds) of AZD5069 administered in combination with enzalutamide at 160mg OD.<br>-Adverse event (toxicity) evaluation (history and examination) was performed by the study investigator (medical oncologist) on a weekly basis, and more frequently if required, during the DLT period (cycle 1) as well as during cycle 2 of the study, and then at least once every 4 weeks thereafter.<br>-Adverse events were graded using the National Cancer Institute (NCI) CTCAE (v4.0) criteria.<br>-The DLT and MTD were adverse events defined using the NCI CTCAE v4.0 criteria (full list of definitions are listed in the protocol,

section 3.2) that were deemed highly probably or probably related to either study drug by the study investigator.

Secondary endpoints:
1. Antitumor activity was defined by the rate of objective response. If any of these occur, patients will be considered to have responded:
---- PSA decline ≥ 50% criteria confirmed 4 weeks or later and/or,
---- confirmed soft tissue objective response by RECIST (v1.1) in patients with measurable disease and/or,
---- ONLY for patients with detectable circulating tumour cell count (CTC) of ≥5/7.5ml blood at baseline, conversion of CTC <5/7.5ml blood nadir.
----Patients must receive 12 weeks of trial treatment to be considered to have responded. .
--Disease progression is defined as:
----Progression of soft tissue/visceral disease by RECIST (v1.1) and/or,
----Progression of bone disease by PCWG2 bone scan criteria and/or,
----Progression of PSA by PCWG2 PSA criteria and/or
----Unequivocal clinical progression.
2 rPFS was be measured from the date of AZD5069 addition to enzalutamide until:
-- Progression of soft tissue/visceral disease by RESIST and/or,
-- progression of bone disease by PCWG2 bone scan criteria and/or,
-- death from any cause
3. OS was be measured from the date of AZD5069 addition to enzalutamide to the date of death (whatever cause).
4. Determination of the plasma levels of enzalutamide and AZD5069 using validated assays
5. Identify those patients with a NLR ≥ 3 (at baseline) that convert to an NLR < 3 (blood nadir) with AZD5069 and enzalutamide in combination.
6. Identify those patients whose circulating myeloid derived suppressor cells (MDSCs) and intratumoral MDSCs reduce by 50%

Key exploratory endpoints:
1. Evaluating the effect of AZD5069 treatment on circulating cytokine levels in whole blood using an enzyme-linked immunosorbent assay (ELISA).
2. Evaluation of the impact of CXCR2i depending on tumor molecular profile, such as PTEN loss.

Radiologic tumor responses were measured by CT of the thorax, abdomen, and pelvis bone scan and bone scan, and where indicated, whole body magnetic resonance imaging, at baseline, once every 3 cycles, then at the end of treatment if this was more than eight weeks since the last scan. PSA and CTC count were measured at baseline, then on day 1 of every cycle and at the end of treatment visit. PSA responses were determined by PCWG2 criteria. CTC counts were analysed using previously described methods (Mateo et al., NEJM, 2015, Goodall et al., Cancer Discovery, 2017). Patients need to be on trial for at least 12 weeks to be considered to have responded. NLR was a quotient of the neutrophil and lymphocyte count taken on study (from routine hematology bloods at baseline and serially on day 1 of every cycle). Immune cell densities were determined by IF of pre and on-treatment biopsies taken before and after starting trial treatment. All slides were QC'd and reviewed by the pathologist (BG) and histopathology scientist (MC). PK data were obtained from PK bloods taken at prespecified time points (baseline, start of cycle 1 and 2) evaluating for plasma levels of enzalutamide and AZD5069 for patients in dose levels 1-4 . Bloods for serum cytokine analyses were collected at prespecified timepoints per the trial protocol (baseline, day 1 of every cycle, and day 15 of the first cycle).
